# Identification of novel genome-wide associations for suicidality in UK Biobank, genetic correlation with psychiatric disorders and polygenic association with completed suicide

**DOI:** 10.1016/j.ebiom.2019.02.005

**Published:** 2019-02-08

**Authors:** Rona J. Strawbridge, Joey Ward, Amy Ferguson, Nicholas Graham, Richard J. Shaw, Breda Cullen, Robert Pearsall, Laura M. Lyall, Keira J.A. Johnston, Claire L. Niedzwiedz, Jill P. Pell, Daniel Mackay, Julie Langan Martin, Donald M. Lyall, Mark E.S. Bailey, Daniel J. Smith

**Affiliations:** a Institute of Health and Wellbeing, University of Glasgow, Glasgow, UK; b Department of Medicine Solna, Karolinska Institute, Stockholm, Sweden; c Division of Psychiatry, College of Medicine, University of Edinburgh, UK; d School of Life Sciences, College of Medical, Veterinary and Life Sciences, University of Glasgow, Glasgow, UK

## Abstract

**Background:**

Suicide is a major issue for global public health. Suicidality describes a broad spectrum of thoughts and behaviours, some of which are common in the general population. Although suicide results from a complex interaction of multiple social and psychological factors, predisposition to suicidality is at least partly genetic.

**Methods:**

Ordinal genome-wide association study of suicidality in the UK Biobank cohort comparing: ‘no suicidality’ controls (*N* = 83,557); ‘thoughts that life was not worth living’ (*N* = 21,063); ‘ever contemplated self-harm’ (*N* = 13,038); ‘act of deliberate self-harm in the past’ (*N* = 2498); and ‘previous suicide attempt’ (*N* = 2666).

**Outcomes:**

We identified three novel genome-wide significant loci for suicidality (on chromosomes nine, 11 and 13) and moderate-to-strong genetic correlations between suicidality and a range of psychiatric disorders, most notably depression (r_g_ 0·81).

**Interpretation:**

These findings provide new information about genetic variants relating to increased risk of suicidal thoughts and behaviours. Future work should assess the extent to which polygenic risk scores for suicidality, in combination with non-genetic risk factors, may be useful for stratified approaches to suicide prevention at a population level.

**Fund:**

UKRI Innovation-HDR-UK Fellowship (MR/S003061/1). MRC Mental Health Data Pathfinder Award (MC_PC_17217). MRC Doctoral Training Programme Studentship at the University of Glasgow (MR/K501335/1). MRC Doctoral Training Programme Studentship at the Universities of Glasgow and Edinburgh. UKRI Innovation Fellowship (MR/R024774/1).

**Research in context**

**Evidence before this study**

To date genetic studies of suicidal behaviour (ideation, attempt, completion) have mainly been conducted in individuals with severe mental illness and the few findings reported have failed to replicate in subsequent studies.

**Added value of this study**

This is the first study to explore the genetics of a broad suicidality phenotype within a large population-based cohort (*N* = 122 k individuals). Mutually exclusive categories of ‘no suicidal behaviour’, ‘contemplated self-harm’, ‘actual self-harm’, ‘suicidal ideation’ and ‘suicide attempt’ were assessed in an ordinal genome-wide association study (GWAS). A risk score of suicidality demonstrated associations with an independent subset of completed suicide. Moderate-to-strong genetic correlations were observed with all major psychiatric traits. Separate GWAS analyses of deliberate self-harm or suicide ideation and attempts suggested that the genetic contributions to these traits have distinct components. In a recent independent study of suicide attempts, the lead genetic variant identified in our study demonstrated consistent replication of effect size and direction.

**Implications**

This is the first report of robust genetic associations with suicidality and has the potential to lead to improved understanding of biological mechanisms underlying suicidal thoughts and behaviour. Further exploration of the impact of genetic risk scores in combination with clinical, social and environmental factors is now warranted.

Alt-text: Unlabelled Box

## 1 Introduction

Suicide is a major and growing issue for global public health. Annually, approximately 800,000 people die by suicide and 20 times this number will attempt suicide during their lifetime [1]. ‘Suicidality’ encompasses a broad range of experiences and behaviours, from suicidal ideas/thoughts, to acts of deliberate self-harm and suicide attempts, occurring along a spectrum towards completed suicide [2]. Some components of suicidal thoughts and behaviours, such as feeling that life is not worth living or contemplating self-harm, are relatively common in the general population, as well as in patients affected by a variety of separate clinical diagnoses. Suicidality can therefore be considered a complex dimensional trait that fits within a Research Domain Criteria (RDoC) approach because it cuts across traditional psychiatric diagnostic classifications.

Pathways to completed suicide are complex and multifactorial [3]. Suicidal thoughts and actions are a consequence of a dynamic interplay between genetic, other biological, psychiatric, psychological, and a wide range of important social, economic and cultural factors [4]. Clinically, deliberate self-harm (DSH) is a major risk factor for subsequent suicidal behaviour. It is also recognised that substance abuse-related disorders and mood disorders are particularly associated with suicide risk [5]. Similarly, early adversities such as childhood sexual abuse [6], maladaptive parenting [7] and parental loss [8] all contribute to suicidal thoughts and behaviours, either directly or by increasing the risk of psychiatric disorders [9,10]. Personality-level traits such as neuroticism, impaired decision-making and sensitivity to negative social stimuli also contribute to suicidality [11].

Family, adoption and twin studies suggest a heritability estimate for suicidal behaviour of approximately 38–55% [12], and thus suicidality as a behavioural trait is amenable to genetic investigation. Heritability estimates for less clearly defined phenotypes such as suicidal thoughts are difficult to establish [13]. It is clear, however, that genetic predisposition plays a role in suicide alongside individual and social factors. Genetic studies may offer some insight into the biological basis of suicidality but genome-wide association studies (GWAS) [[14], [15], [16], [17], [18], [19], [20], [21], [22], [23], [24]] or family-based [25] findings to date have been limited, perhaps as a result of subsequent studies being under-powered or because of diagnostic heterogeneity. Recent advances in this area include a GWAS of suicide attempts in approximately 50,000 individuals with and without psychiatric disorders which identified some suggestive loci [26], replication of a single GWAS-significant finding on Chromosome 2 [17], nominal evidence for replication of a SNP on Chromosome 6 [22] and a locus on Chromosome 3 [15] and demonstration that suicide attempt and clinically predicted suicide share significant heritability [27].

Our goals in this study were: to identify genetic variants associated with broadly-defined suicidality in 122,935 participants of the UK Biobank cohort; to assess for genetic correlations between suicidality and a range of psychiatric disorders; and to determine whether increased genetic burden for suicidality was associated with both psychiatric disorders and completed suicide in a non-overlapping sample. Broadly-defined suicidality included thoughts and actions of both suicide and deliberate self-harm, despite current debate over the extent to which deliberate self-harm and suicidal intent overlap. Our primary analyses used dimensions of suicidality ordered to reflect clinical severity: from no suicidal thoughts or behaviours, thoughts that life is not worth living, considered self-harm, actual self-harm and attempted suicide. In secondary analyses, mindful that not all DSH behaviours necessarily carry active suicidal intent, we also conducted additional separate GWAS analyses of DSH and suicidal ideation/attempts (SIA).

## 2 Materials and methods

### 2.1 Sample description

UK Biobank is a large general population cohort. Between 2006 and 2010, approximately 502,000 participants (age range 37–73 years) were recruited and attended one of 22 assessment centres across mainland UK [28,29]. Comprehensive baseline assessments included sociodemographic characteristics, cognitive abilities, lifestyle and measures of mental and physical health status (Supplementary Methods and Supplementary Fig. 1). To maximise sample homogeneity, only white British participants were included in the current analysis. Informed consent was obtained by UK Biobank from all participants. This study was carried out under the generic approval from the NHS National Research Ethics Service (approval letter dated 13 May 2016, Ref 16/NW/0274) and under UK Biobank approval for application #6553 “Genome-wide association studies of mental health” (PI Daniel Smith).

### 2.2 Suicidality phenotypes

Suicidality groups were based on four questions from the self-harm behaviours section of the online mental health (*‘Thoughts and Feelings’)* questionnaire administered in 2016/2017: (http://biobank.ctsu.ox.ac.uk/crystal/label.cgi?id=136 and Supplementary Methods [30]). Non-overlapping categories of increasing severity of suicidality were derived: ‘no suicidality’ controls; ‘thoughts that life was not worth living’; ‘ever contemplated self-harm or suicide’; ‘acts of deliberate self-harm not including attempted suicide’; ‘attempted suicide’. If participants met criteria for more than one category they were assigned to the most severe category. Those in the lowest category were required to have answered “no” to all the questions. Linkage to death certification (until February 2016) identified a separate sub-group of participants classified as ‘completed suicide’ (defined as primary cause of death by intentional self-harm, ICD codes X60-X84; *N* = 137). The latter group was not used in the ordinal GWAS but rather was used in a separate analysis to test for association with genetic loading for suicidality. Participants were classified based on the most extreme form of suicidality that they reported and placed within the ‘no suicidality’ group if they responded negatively to all self-harm and suicidality questions. Those who preferred not to answer any of the questions (0·7%) were excluded from analysis.

### 2.3 Genotyping, imputation and quality control

In July 2017 UK Biobank released genetic data for 487,409 individuals, genotyped using the Affymetrix UK BiLEVE Axiom or the Affymetrix UK Biobank Axiom arrays (Santa Clara, CA, USA) [29]. These arrays have over 95% content in common. Pre-imputation quality control, imputation and post-imputation cleaning were conducted centrally by UK Biobank (described in the UK Biobank release documentation [28,29]. Fully imputed genetic data released in March 2018 were used for this study, therefore a total of 8,930,390 SNPs were available for analysis.

### 2.4 Ordinal GWAS of suicidality, DSH and SIA

For each GWAS, we excluded at random one person from each related pair of individuals with a kinship coefficient > 0·042 (second cousins) that have valid phenotypes, therefore the number of controls and participants within each suicidality level is different for each analysis (Supplementary Methods and Supplementary Fig. 1, Supplementary Fig. 2).

The primary GWAS included 122,935 individuals. Of these, 83,557 were classified as controls (category 0), 21,063 were classified in the ‘thoughts that life is not worth living’ group (category 1), 13,038 in the ‘thoughts of self-harm’ group (category 2), 2498 in the ‘actual self-harm’ group (category 3), and 2666 in the ‘attempted suicide’ group (category 4).

For the secondary analyses, two further ordinal GWAS were conducted. For DSH, the categories of controls, (*N* = 84,499), ‘thoughts of self-harm’ (*N* = 13,203) and ‘actual self-harm’ (*N* = 2532) were assessed. For SIA, the categories of controls (N = 84,167), ‘thoughts that life is not worth living’ group (*N* = 21,234) and ‘attempted suicide’ (*N* = 2689) were assessed.

Analyses were performed in R (Version 3.1) using the clm function of the ordinal package [31] treating the multilevel suicidality, DSH or SIA outcome variable as an ordinal variable. Models were adjusted for age, sex, genotyping chip and UK Biobank-derived genetic principal components (GPCs) 1–8. For sensitivity analyses, a variable for psychiatric diagnosis was included as a covariate (where psychiatric diagnosis was defined as likely or self-reported bipolar disorder (BD), Generalized Anxiety Disorder (GAD) and Major Depressive Disorder (MDD) and schizophrenia (SZ)) [30,32]. Further sensitivity analyses also included self-reported childhood sexual abuse as a covariate [33]. Genome-wide significance was set at *P* < 5 × 10^−8^ and plots were generated using FUMA [34].

### 2.5 Polygenic Risk score (PRS) variables for suicidality, mood disorders and related traits

Polygenic risk scores (PRS) were calculated from the primary ordinal suicidality GWAS summary statistics after pruning based on linkage disequilibrium (Supplementary Methods). SNPs were included in the PRS if they met *p*-value thresholds of *p* < 5 × 10^−8^, *p* < 5 × 10^−5^, *p* < 0·01 *p* < 0·05, *p* < 0·1 or *p* ≤ 0·5 (Supplementary Methods). PRS deciles were computed using STATA (version 12, STATACorp) and modelling of associations between the PRS and completed suicide was analysed with logistic regression, adjusting for age, sex, chip and GPCs 1–8. In this analysis, cases were individuals classified as ‘completed suicide’ (*n* = 127), and controls were those recorded as category 0 in the ordinal variable but who had been excluded from the GWAS due to relatedness (*n* = 5330). It should be noted that the category 0 individuals here were related to individuals across the spectrum of suicidality in the GWAS, thus are more representative of the general population distribution of PRS than the true “no suicidality” distribution. Therefore this analysis is a conservative approach, being biased towards the null. Associations between the PRS and risk of mood disorders and related traits were also assessed (Supplementary Methods). The traits tested (BD, MDD, mood instability, and risk-taking behaviour) were selected based on prior evidence of relevance to suicidality, therefore the threshold for significance was set at *p* < 0·05.

### 2.6 SNP heritability and genetic correlation analyses

Linkage Disequilibrium Score Regression (LDSR) [35] was used to estimate the SNP heritability (h^2^_SNP_) of ordinal suicidality, DSH and SIA. LDSR was also used to calculate genetic correlations with suicide attempt, psychiatric disorders and related traits (Supplementary Methods). The resulting genetic correlation *P*-values were false discovery rate (FDR)-corrected to compensate for multiple testing.

### 2.7 Gene-based analysis

The ordinal GWAS results were also considered under a gene-based approach, using MAGMA [36], as implemented in FUMA [34].

### 2.8 Exploration of known biology

The Variant Effect Predictor web-based tool [37], GTEx database [38] and BRAINEAC dataset (http://braineac.org/) were interrogated to try to identify genes (based on expression quantitative trait loci, eQTLs) or mechanisms through which associated SNPs might be acting (Supplementary Methods). The GWAS catalogue (https://www.ebi.ac.uk/gwas/) and NCBI Gene https://www.ncbi.nlm.nih.gov/gene/) were queried for each of the suicidality-associated SNPs and genes reported here.

## 3 Results

### 3.1 Sociodemographic characteristics

Sociodemographic, clinical and health-related behaviour measures for each of the suicidality categories are shown in Supplementary Table 1. As expected, a gradient of increasing suicidality was found for increasing levels of social deprivation, living alone, current or previous smoking, parental depression and chronic pain. There were also substantial differences by sex: females accounted for 68·3% of those who reported attempted suicide but only 27·6% of completed suicides. A large proportion of those who had attempted suicide (85·1%) had a history of MDD, compared to only 14·9% of controls. Similarly, 75·8% of those with a suicide attempt also reported childhood trauma, compared to 39·0% of controls.

### 3.2 Primary ordinal GWAS of suicidality

**Fig. 1:**
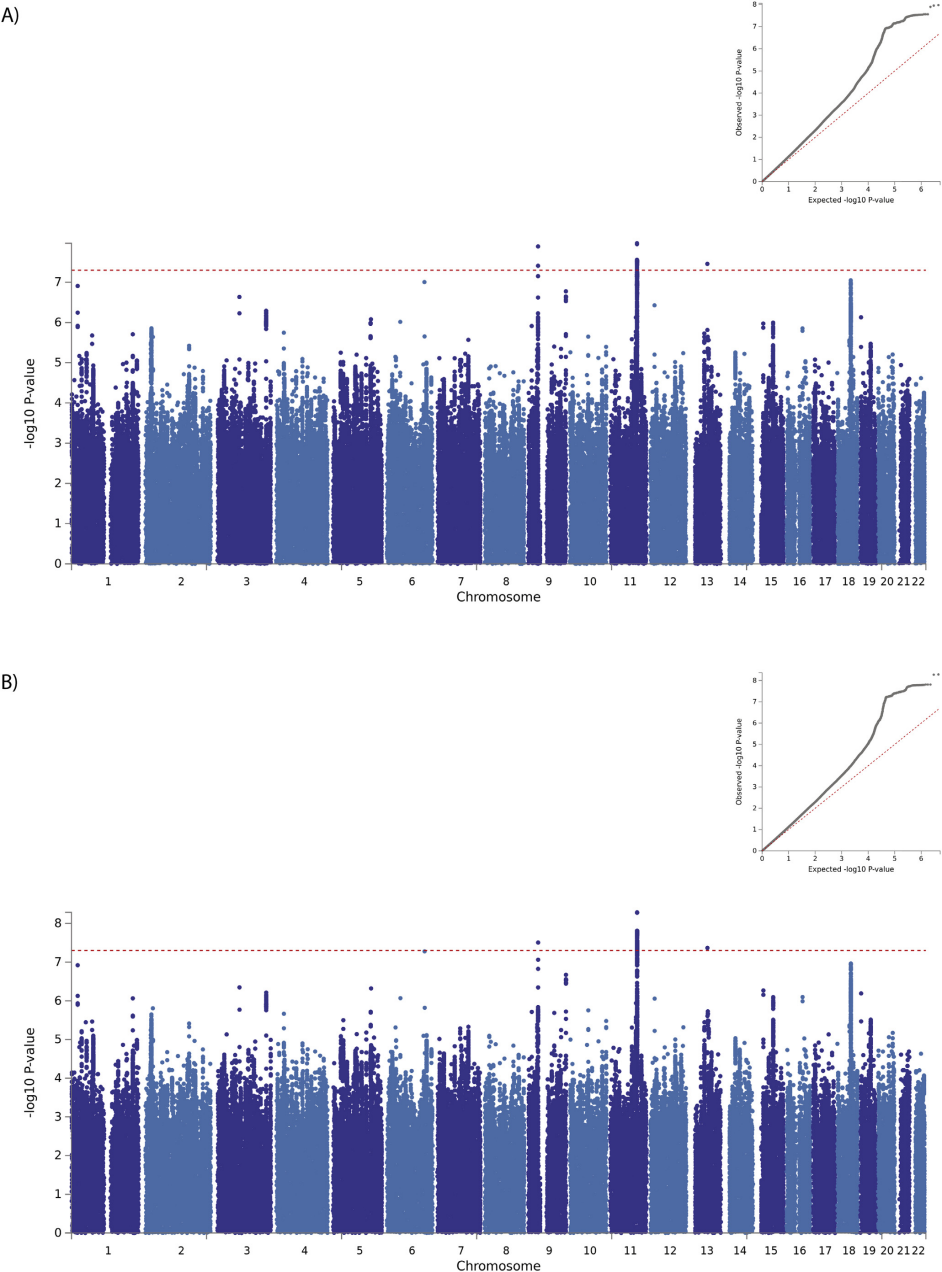
Manhattan plot of GWAS of ordinal suicidality in UK Biobank (N = 122,935): A) adjusted for age, sex, genotyping chip and population structure, B) adjusted for age, sex, genotyping chip, population structure and psychiatric disorders. Dashed red line = genome wide significance threshold. Inset: QQ plot for genome-wide association with ordinal suicidality. Red line = theoretical distribution under the null hypothesis of no association. (For interpretation of the references to colour in this figure legend, the reader is referred to the web version of this article.)

The results of the ordinal GWAS of suicidality are presented in Table 1, Supplementary Table 2 and Fig. 1A. The GWAS results showed some inflation of the test statistics from the null (λ_GC_ = 1·16, Fig. 1A, inset) but this was not significant given the sample size used (λ_GC_ 1000 = 1·004). LDSR demonstrates that polygenic architecture, rather than unconstrained population structure, is the likely reason for this (LDSR intercept = 1·02, SE = 0·0075). SNP heritability was estimated by LDSR as being 7·6% (observed scale h^2^_SNP_ = 0·076; SE = 0·006).

**Fig. 2:**
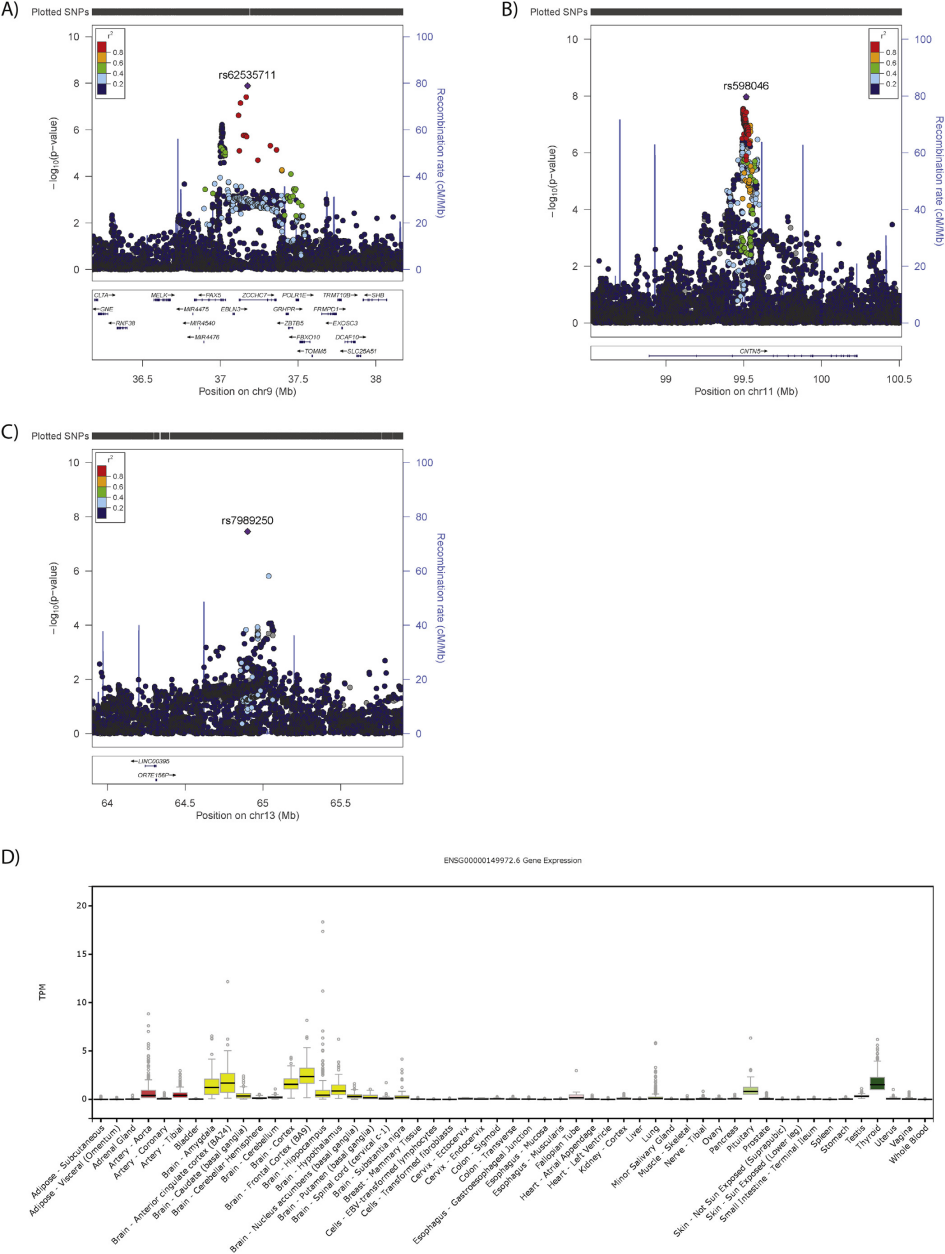
Regional plots for GWAS significant loci and *CNTN5* tissue expression: A) *ZCCHC7* locus on Chr9, B) *CNTN5* locus on Chr11, C) Chr13 locus, where: SNPs (each point) are aligned according to position (X axis) and strength of association (Y Axis, left); Purple colouring indicates the index SNP, with other colours representing linkage disequilibrium (r^2^) with the index SNP, as per the colour key; Recombination rate is presented as a pale blue line graph in the background (Y axis, right); Genes are presented below the association plot by location (X axis) and direction of transcription (arrows). D) Tissue expression profile of *CNTN5*, where tissues are arranged alphabetically along the X-axis and expression level is (TPM; standardised transcripts per million reads) provided on the Y-axis. Box plots represent median and interquartile range, with error bars demonstrating 1.5× the interquartile range and dots representing outliers. (For interpretation of the references to colour in this figure legend, the reader is referred to the web version of this article.)

We identified three independent loci associated with suicidality (Table 1, Supplementary Table 2 and Fig. 1A): one on chromosome 9 (index SNP rs62535711, Fig. 2A) within the gene *ZCCHC7*; a second on chromosome 11 (index SNP rs598046, Fig. 2B) located within *CNTN5;* and a third on chromosome 13 (index SNP rs7989250, Fig. 2C). Conditional analyses (Supplementary Methods) in which the lead SNP was included as a covariate demonstrated no significant secondary association signals at these loci (the most significant additional SNP on chromosome 9 was rs999510, *p* = 0·0008; that on chromosome 11 was rs608820, *p* = 0·0005; and that on chromosome 13 was rs9564176, *p* = 0·003). Effect allele frequencies by suicidality category are presented in Supplementary Table 3. Adjustment of the GWAS for psychiatric disorders had little or no effect on the observed associations (Fig. 1B and Supplementary Table 2), whilst adjustment for childhood sexual abuse rendered all associations null (Supplementary Fig. 3).

It is notable that within the recently-reported GWAS of suicide attempt in a Danish sample [26], rs62535711 and rs7989250 were not significant (*p* = 0·278 and *p* = 0·152 respectively) but rs598046 was reported as borderline significant (G allele, Beta 0·041, SE 0·021 *p* = 0·051).

**Table 1:** Lead SNPs at loci associated with ordinal suicidality at GWAS significance.

Analysis	SNP	CHR	POS	A1	A2	BETA	SE	*P*	A1F^a^	SNPs_gwas^b^	SNPs_sugg^b^
Ordinal suicidality	rs62535711	9	37,174,829	T	C	0.105	0.018	1.29E-08	0.056	15	57
	rs598046	11	99,516,468	T	G	0.053	0.009	1.07E-08	0.319	34	370
	rs7989250	13	64,900,801	A	C	−0.052	0.009	3.49E-08	0.322	1	5

Where: A1, effect allele; A2, other allele; A1F, effect allele frequency; Aligned to Human Genome assembly GRCh37, Chr9 locus, 9:36999369–37,360,767; Chr11 locus, 11:99392678–99,588,751; Chr13 locus, 13:64900801–65,036; Chr6 locus, 6:140442326–140,895,470; SIA, suicidal ideation or attempt.

a Calculated in whole cohort.

b Within the region defined by suggestive significance (*P* < 1 × 10^–^^5^).

### 3.3 Association between genetic loading for suicidality and ‘completed suicide’

Demographic characteristics of the controls in this analysis were comparable to those included in the GWAS (Supplementary Table 1). Individuals within the completed suicide group followed a similar pattern of increasing deprivation, more childhood trauma and higher prevalence of mood disorders observed across the categories of increasing suicidality (Supplementary Table 1). We investigated whether greater genetic loading for suicidality, indexed by PRS for suicidality, was associated with completed suicide. Overall, higher values of suicidality PRS were associated with an increased risk of completed suicide at all but one of the PRS significance thresholds assessed (for example, *p-*threshold 0·05: OR 1.23, 95%CI 1·06–1·41, FDR-adjusted *p* = 0·04, Table 2).

### 3.4 Genetic loading for suicidality and mood disorders

PRS for suicidality also demonstrated consistent significant associations with mood disorders (BD and MDD) and related traits (mood instability, neuroticism, and risk-taking propensity), across most of the significance thresholds assessed (Supplementary Table 4).

### 3.5 Secondary GWAS analyses

The ordinal GWAS of DSH (comprising controls, contemplated self-harm and actual self-harm) identified no SNPs at genome-wide significance (Supplementary Fig. 4A) and adjustment for psychiatric diagnosis had little impact on these results (Supplementary Fig. 4B): the most significant association was with rs4521702-T, Beta −0·01162, SE 0·0218, *p* = 9·56 × 10^−8^; and Beta −0·01159, SE 0·0218, *p* = 1·10 × 10^−7^, without and with adjustment for psychiatric diagnosis, respectively.

The ordinal GWAS of SIA (comprising controls, ‘suicidal ideation’, and ‘suicide attempts’) also identified no SNPs at genome-wide significance (Supplementary Fig. 5A), however adjustment for psychiatric disorders did identify a genome-wide significant singleton SNP rs116955121 (Supplementary Fig. 5B–C and Supplementary Table 2). In the recent Danish GWAS of suicide attempt [26], rs116955121 was not significant (*p* = 0·509).

### 3.6 Genetic correlation analyses

When considering the whole genome (rather than SNPs selected for association with suicidality, as is the case for the PRS), we observed significant genetic correlations between suicidality (primary analysis) and attempted suicide, and between suicidality and all of the major psychiatric disorders and traits we assessed (Table 3). The strongest genetic correlations were observed for MDD (r_g_ 0·81), Anxiety disorder (r_g_ 0.75), neuroticism (r_g_ 0·63) and mood instability (r_g_ 0·50). DSH demonstrated similar genetic correlations with attempted suicide and with psychiatric disorders and related traits as those observed for suicidality (Table 3). In contrast, for SIA, significant genetic correlations were observed only for MDD, schizophrenia, neuroticism and mood instability (Table 3).

**Table 2:** Increasing burden of suicidal behaviour-associated variants significantly associated with completed suicide.

Threshold	OR	L95	U95	*P*	FDR-adj *P*
5 × 10^−8^	1.07	−0.10	0.24	0.410	0.410
5 × 10^−5^	1.20	0.01	0.35	0.041	0.049
0.01	1.22	0.02	0.38	0.026	0.390
0.05	1.26	0.05	0.41	0.011	0.034
0.1	1.27	0.06	0.42	0.008	0.034
0.5	1.25	0.03	0.41	0.021	0.039

Where: Threshold, GWAS *P* threshold of the SNPs included in the suicidality PRS; OR, odds ratio; L95, lower 95% confidence interval; U95, upper 95% confidence interval; Z, test statistic, *P*, *P* value for analysis; FDR-adj *P*, false discovery rate adjusted *P*. Signficance was set at *P* *<* 0.05.

**Table 3:** Genetic correlations of suicidality with psychiatric disorders and related traits.

Trait	Suicidality					DSH					SIA				
	r_g_	se	z	p	FDR-P	r_g_	se	z	p	FDR-P	r_g_	se	z	p	FDR-P
Attempted suicide	0.57	0.096	5.98	2.23E-09	6.24E-09	0.624	0.106	5.8897	3.87E-09	9.03E-09	−0.012	0.182	−0.068	9.46E-01	9.83E-01
MDD	0.81	0.04	18.66	1.01E-77	1.41E-76	0.79	0.05	14.48	1.68E-47	2.35E-46	0.46	0.11	4.07	4.76E-05	2.22E-04
Neuroticism	0.63	0.04	16.48	5.51E-61	3.86E-60	0.57	0.05	12.09	1.26E-33	8.82E-33	0.56	0.12	4.77	1.80E-06	1.26E-05
Mood Instability	0.50	0.03	16.06	4.53E-58	2.11E-57	0.43	0.04	11.73	8.50E-32	3.97E-31	0.53	0.11	5.01	5.42E-07	7.59E-06
Schizophrenia	0.32	0.04	8.70	3.19E-18	1.12E-17	0.31	0.04	7.02	2.25E-12	7.88E-12	0.28	0.09	2.96	3.06E-03	1.07E-02
Bipolar disorder	0.27	0.05	5.28	1.26E-07	2.94E-07	0.27	0.06	4.34	1.45E-05	2.26E-05	0.19	0.12	1.55	1.21E-01	3.39E-01
Risk-taking behaviour	0.20	0.04	5.05	4.44E-07	7.77E-07	0.25	0.04	6.00	1.93E-09	5.40E-09	0.00	0.08	0.02	9.83E-01	9.83E-01
Anxiety disorder	0.75	0.17	4.35	1.38E-05	2.15E-05	0.87	0.20	4.41	1.03E-05	1.91E-05	0.31	0.30	1.03	3.05E-01	5.34E-01
ADHD	0.21	0.05	4.29	1.79E-05	2.51E-05	0.23	0.05	4.40	1.09E-05	1.91E-05	−0.12	0.11	−1.17	2.44E-01	5.20E-01
PTSD	0.42	0.16	2.70	6.97E-03	8.87E-03	0.52	0.18	2.82	4.82E-03	6.75E-03	0.16	0.28	0.57	5.70E-01	7.98E-01

Where: DSH, deliberate self-harm; SIA, suicidal ideation or attempt; rg, genetic correlation; se, standard error of genetic correlation; z, test statistic, P, P vlaue for analysis; FDR adj P, false discovery rate-adjusted P; Signficicance was set at P ≤ 0.05; MDD, major depressive disorder; PTSD, post-traumatic stress disorder; Relative amplitude, quantitative measure of circadian rythmicity; ADHD, attention deficit hyperactivity disorder.

### 3.7 Gene-based analysis

Gene-based analysis was used to identify genes containing potential composite association signals that were not identified by the individual SNP analysis, but which might nevertheless contribute to biological mechanisms underlying suicidality. Gene-based analysis highlighted *CNTN5, ADCK3/COQ8A*, *CEP57* and *FAM76B* and *DCC* for suicidality (primary analysis, Supplementary Fig. 6), *EIF4A1* and *SENP3* and *DCC* for DSH (Supplementary Fig. 7) and *CDKAL1, CNTN5,* and *ADCK3/COQ8A* for SIA (Supplementary Fig. 8). Regional plots for these genes (except for *CNTN5,* which was also identified in the SNP-based analysis) are presented in (Supplementary Fig. 9).

### 3.8 Biology of the suicidality-associated loci

Suggested functions of genes within the suicidality-associated loci are presented in Supplementary Table 5 and the Supplementary Results. Notable findings were the chromosome 11 locus located within *CNTN5*, a very large gene expressed predominantly in brain in adults (Fig. 2D); eQTL analysis supported the possible involvement of several candidate genes on chromosome 9 (Supplementary Fig. 10 and Supplementary Results), and, in combination with the gene-based analysis, additional candidate genes on chromosome 11 (*CEP57*; Supplementary Table 6) and on chromosome 18 (*DCC*). These genes and nearby variants have previously been associated with a variety of relevant traits (Supplementary Table 7). Of the SNPs with suggestive evidence for association (GWAS *p* < 1 × 10^−5^) with suicidal behaviour in other studies, 29 were available in our analysis and five of these demonstrated nominal (*p* < 0·05) association with suicidality in this study (Supplementary Table 8), although only one of these, rs72940689, had a direction of effect consistent with that given in the previous report [26].

## 4 Discussion

### 4.1 Main findings

Using a very large population-based cohort, we identified multiple genetic loci associated with suicidality. We also found that increased genetic burden for suicidality was associated with increased risk of completed suicide within a non-overlapping sub-sample and there were consistent genetic correlations with a wide range of psychiatric disorders and psychopathological traits, particularly MDD and anxiety disorders. Separate GWAS analyses of DSH and SIA identified one additional signal (for SIA) and suggested that the genetic architecture of DSH is likely to be distinct from that of SIA. Genetic correlations between mental illness and DSH or SIA also differed. More generally, the consistent effect size and direction of the association of rs598046-G (*CNTN5*) with suicide attempts in an independent cohort [26], and our nominal replication of a SNP reported by Erlangsen (rs7862648) is of interest [26].

In line with our evidence that suicidality is polygenic, inclusion of more SNPs (using more relaxed *p*-value thresholds) within a PRS typically demonstrates more significant effects in contrast to the stricter thresholds used because more information and power is provided by including a greater number of SNPs. Currently there is no agreed threshold that should be considered in these analyses, therefore we reported several PRS analyses. Future work on PRSs for suicidality should seek to identify thresholds that optimally facilitate stratification of clinical and non-clinical populations.

### 4.2 Comparisons with previous studies

Direct comparison with previous studies is hindered by the radically different study design reported here. Nonetheless, we have tried to align our findings with those previously reported.

This is the largest GWAS of suicidality to date and the first to consider a broad spectrum of suicidal behaviours. The loci previously reported for suicidal behaviours do not overlap with those identified here [[14], [15], [16], [17], [18], [19], [20], [21], [22], [23], [24], [25], [26]]. However, consistent (albeit borderline or nominal) associations of one of our lead SNPs in the recent large Danish study of suicide attempts [26] and one of their lead SNPs in this study is compelling.

Most previous genetic studies of suicidal behaviour and completed suicide (Supplementary Table 8) have been conducted in cohorts with known diagnoses of major mental illness, thereby controlling for mental illness. In this study, sensitivity analysis controlling (by adjustment) for mental illness had negligible effects on the primary suicidality results, however an additional signal was identified for SIA in secondary analyses. It is likely that the various aspects of suicidality have complex relationships with different mental illnesses. Therefore, we cannot exclude the possibility that the results might have been driven by the mixed and different genetic loadings for psychiatric disorders between suicidal and non-suicidal participants. A further sensitivity analysis adjusting for mental illness and childhood sexual abuse rendered all associations null, however as childhood sexual abuse is strongly associated with mental illness, this model could be considered overly conservative.

In addition, the limited overlap between suicidality-associated loci identified across these studies also likely reflects substantial differences in recruitment protocols, participant characteristics (including the mix of psychiatric diagnoses) and variation in the methods of assessment of suicidal behaviour. Recently, a general population study in Denmark of suicide attempts identified genetic loci at genome-wide significance [26]. Our study extends this approach by investigating a broader phenotype within our primary analysis, as well as the specific impact of DSH versus SIA in secondary analyses. In line with a Research Domain Criteria (RDoC) approach, we used the full spectrum of suicidal thoughts and behaviours assessed within a predominantly non-clinical population. The fact that increased genetic burden for suicidality was associated with increased risk of completed suicide in a separate sub-sample represents an important validation of our suicidality phenotype. The genetic correlation with MDD was strong, but the incomplete overlap, and the fact that the GWAS results were largely unchanged by adjustment for mental health disorder status, supports the hypothesis that at least some of the genetic predisposition to suicidal ideation and behaviours may be distinct from that for MDD [18].

The SNP-based heritability reported here for suicidality (7.6%) is more than that reported for suicide attempts (4.6%) [26], however both of these are lower than heritability estimates from family studies [12], which is consistent with findings from most other complex traits studied to date.

### 4.3 Biology

The known biology of the suicidality-associated loci (Supplementary Results) highlights three interesting candidate genes: *CNTN5*, *CEP57* and *DCC*. *CNTN5* encodes contactin 5 (also known as NB-2), which is a good functional candidate. CNTN5 is a glycosylphosphatidylinositol (GPI)-anchored extracellular cell adhesion protein of the immunoglobulin superfamily, thought to have a role in the formation and maintenance of brain circuitry [39]. Centrosomal protein of 57 kDa (CEP57, encoded by *CEP57*) is important for cell division, with loss of function variants causing a mosaic variegated aneuploidy syndrome, which can include brain abnormalities and mental retardation (OMIM #607951 and #614114). The netrin 1 receptor (encoded by *DCC*) has been robustly associated with depression [40], schizophrenia [41] and related traits [42]. Speculation as to how variation in these genes act to influence these related traits is difficult because of incomplete understanding of the functions of these genes in the brain during development and aging.

### 4.4 Strengths and limitations

This is the largest genetic study of suicidality in a population sample reported to date but we acknowledge some limitations to this work. The nature of the data collected did not allow us to distinguish between putative subtypes of suicide, such as stress-responsive and non-stress-responsive suicidality [43]. In addition, both recruitment bias and recall bias are possible within the UK Biobank dataset. Survivor bias might also influence our findings, due to the relatively older age at recruitment, however this would likely lead to more conservative effect estimates. We also recognise that the questions used to create the ordinal suicidality phenotype mean that some individuals with either self-harm ideation/behaviour or suicidal ideation might be included at levels higher or lower than their suicidality predisposition merits. Our SNP heritability estimates were similar to those reported for other complex psychiatric phenotypes such as MDD [44] and the overlap with clinically relevant phenotypes (at the levels of loci, PRS and whole-genome genetic correlations) all suggest that our findings are robust.

### 4.5 Implications for future work

This study highlights a component of suicidal predisposition that is distinct from MDD predisposition and the potential relevance of *CNTN5, CEP57* and *DCC* to suicidality, further study of which may provide valuable insight into the underlying biology of suicide. Genetic vulnerability to suicide is of course likely to be only a small part of the overall pathophysiology of what is clearly a highly complex and clinically and psychologically heterogeneous phenotype. A major current challenge for the field of suicide research is to integrate new discoveries on the genetics of suicide with known psychiatric, social, psychological and environmental risk factors (such as poverty, substance misuse and childhood sexual abuse), to develop more sophisticated models of risk, and ultimately to develop genetically-informed social, psychological and public health interventions.

## 5 Conclusions

In the largest GWAS to date of suicidality to date we identified several new candidate genes that may be relevant to the biology of completed suicide. We also found substantial genetic correlation between suicidality and a range of psychiatric disorders and, by finding an association between genetic loading for suicidality and completed suicide, we provide preliminary evidence for the potential utility of PRSs for patient and population stratification. We hope these discoveries will facilitate new avenues of research on this complex but clinically important phenotype.

The following are the supplementary data related to this article.

Supplementary Fig. 1 Flow chart of UK Biobank participants available for primary analyses (Ordinal GWAS and PRS analysis) Supplementary Fig. 1

Supplementary Fig. 2 Flow chart of UK Biobank participants available for secondary analyses. The flow chart of participants is the same as Supplementary Fig. 1 up to the highlighted box. Relatedness exclusions were applied for A) the DSH GWAS considering the categories Controls, Contemplated self-harm and Actual self-ham and B) the SIA GWAS considering the categories Controls, Suicidal ideation and attempted suicide. Supplementary Fig. 2

Supplementary Fig. 3 Manhattan plot of GWAS of ordinal suicidality in UK Biobank (*N* = 100,234), adjusted for age, sex, genotyping chip, population structure, psychiatric disorders and childhood sexual abuse. Dashed red line = genome wide significance threshold (*p* < 5 × 10^−5^). Inset: QQ plot for genome-wide association with DSH. Red line = theoretical distribution under the null hypothesis of no association. Supplementary Fig. 3

Supplementary Fig. 4 Manhattan plot of GWAS of ordinal DSH in UK Biobank (N = 100,234). Dashed red line = genome wide significance threshold (p < 5 × 10^−5^). Inset: QQ plot for genome-wide association with DSH. Red line = theoretical distribution under the null hypothesis of no association. Supplementary Fig. 4

Supplementary Fig. 5 Manhattan plot of GWAS of ordinal SIA in UK Biobank (*N* = 108,090). Dashed red line = genome wide significance threshold (p < 5 × 10^−5^). Inset: QQ plot for genome-wide association with SIA. Red line = theoretical distribution under the null hypothesis of no association. Supplementary Fig. 5

Supplementary Fig. 6 Manhattan plot of gene-based GWAS of ordinal suicide in UK Biobank (*N* = 122,935). Dashed red line = genome wide significance threshold (p < 5 × 10^−5^). Inset: QQ plot for genome-wide association with suicidality in UK Biobank. Red line = theoretical distribution under the null hypothesis of no association. Supplementary Fig. 6

Supplementary Fig. 7 Manhattan plot of gene-based GWAS of ordinal DSH in UK Biobank (N = 100,234). Dashed red line = genome wide significance threshold (p < 5 × 10^−5^). Inset: QQ plot for genome-wide association with suicidality in UK Biobank. Red line = theoretical distribution under the null hypothesis of no association. Supplementary Fig. 7

Supplementary Fig. 8 Manhattan plot of gene-based GWAS of ordinal SIA in UK Biobank (N = 108,090). Dashed red line = genome wide significance threshold (p < 5 × 10^−5^). Inset: QQ plot for genome-wide association with suicidality in UK Biobank. Red line = theoretical distribution under the null hypothesis of no association. Supplementary Fig. 8

Supplementary Fig. 9 Regional plots for GWAS significant loci identified in the gene-based analyses. Highlighted genes for suicidality A) *ADCK3/COQ8A* on Chromosome 1, B) *CEP57-FAM76B-MTMR2* on Chromosome 11, C) *DCC* on Chromosome 18, For DSH D) *SENP3* on Chromosome 17 and for SIA E) *CDKAL1* on Chromosome 6. SNPs (each point) are aligned according to position (X axis) and strength of association (Y Axis, left); Purple colouring indicates the index SNP, with r^2^ linkage disequilibrium with the index SNP being presented by colour as per the colour key; Rates of DNA recombination are presented as a pale blue line graph in the background (Y axis, right); Genes are presented by location (X axis) and direction of transcription (arrows). Supplementary Fig. 9

Supplementary Fig. 10 Genotype-specific gene expression of the Chromosome 9 lead SNP, rs62535711 on transcripts of *FRMPD1, MELK, TRMT10B, ZCCHC7* and *GRHPR*, (Supplementary Fig. 3 E–H) in cerebellar cortex (CRBL), frontal cortex (FCTX), hippocampus (HIPP), medulla (specifically inferior olivary nucleus, MEDU), occipital cortex (specifically primary visual cortex, OCTX), putamen (PUTM), substantia nigra (SNIG), thalamus (THAL), temporal cortex (TCTX) and intralobular white matter (WHMT). Supplementary Fig. 10

Supplementary Table1 Cohort demographics of individuals included in the Ordinal suicidality GWAS and the completed suicide PRS analysis Supplementary Table1

Supplementary Table 2 All SNPs associated with ordinal suicidality at GWAS significance Supplementary Table 2

Supplementary Table 3 Allele frequencies of lead SNPs by suicidality category Supplementary Table 3

Supplementary Table 4 Effect of genetic loading for suicidal behaviour on psychiatric disorders and related traits. Supplementary Table 4

Supplementary Table 5 Effect of genetic loading for suicidal behaviour on traits of relevance to psychiatric disorders Supplementary Table 5

Supplementary Table 6 Functions of genes in novel suicidality loci eQTLs in brain of predicted functional SNPs in the *CEP57-FAM76B* locus Supplementary Table 6

Supplementary Table 7 Novel Suicidal behaviour loci and previous associations in the GWAS catalogue Supplementary Table 7

Supplementary Table 8 Previously reported suicidal behaviour-associated SNPs Supplementary Table 8

Supplementary material Image 1
