# Supplementary figures and images for: Identification of novel genome-wide associations for suicidality in UK Biobank, genetic correlation with psychiatric disorders and polygenic association with completed suicide
Source: eBioMedicine. 2019 Feb 8;41:517–25. doi: 10.1016/j.ebiom.2019.02.005 (PMC6442001; doi:10.1016/j.ebiom.2019.02.005)

Supplemental Figure 1

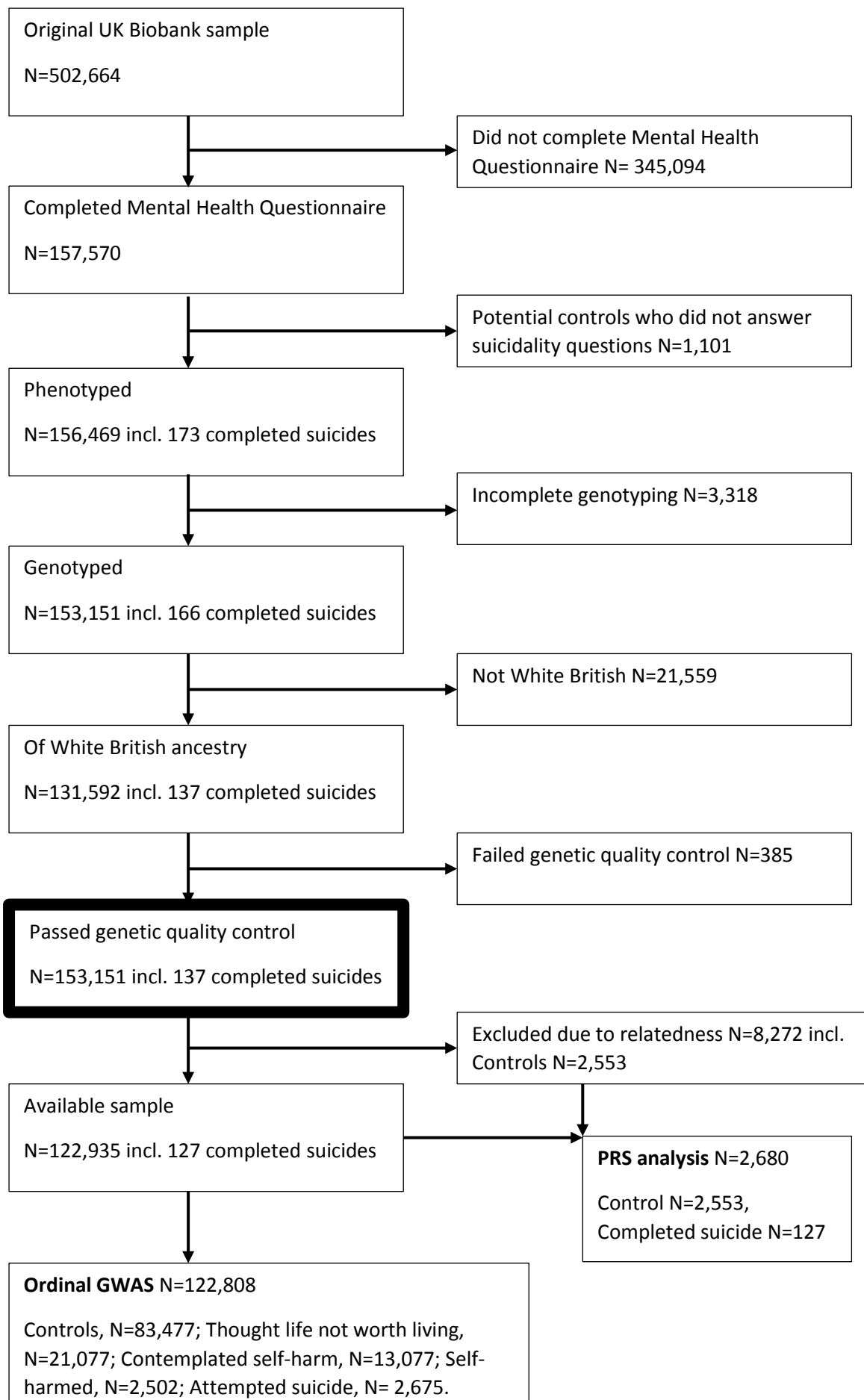

Supplement: Supplementary Fig. 1 — Flow chart of UK Biobank participants available for primary analyses (Ordinal GWAS and PRS analysis) [file mmc1.pdf]

Supplementary Figure 2

A)

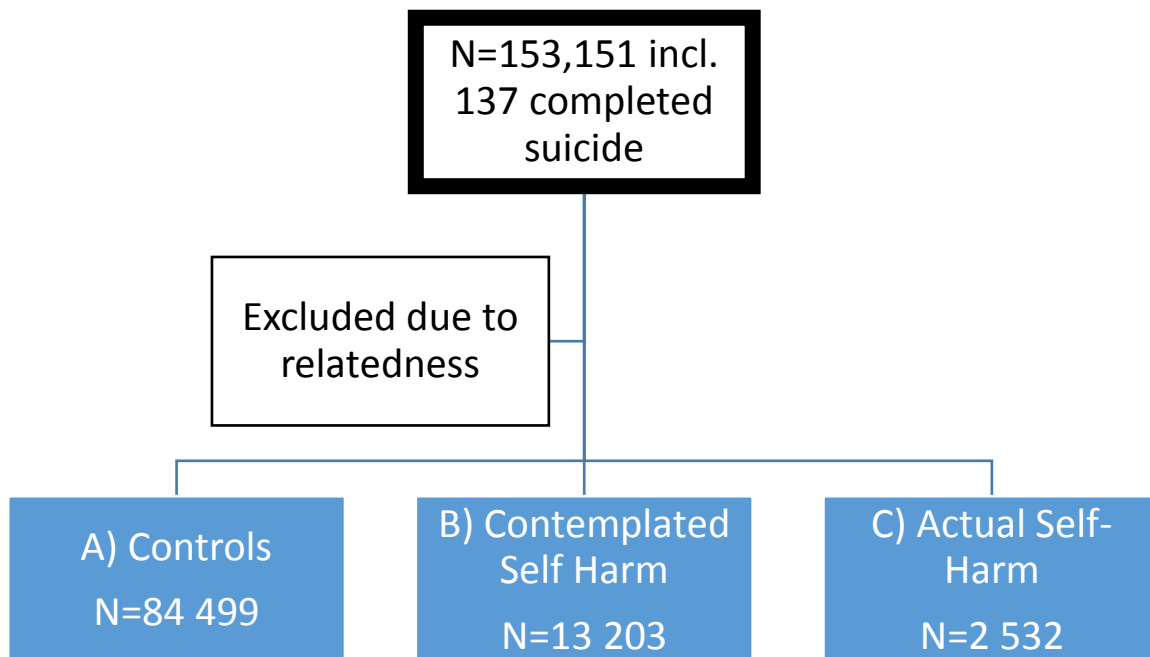

B)

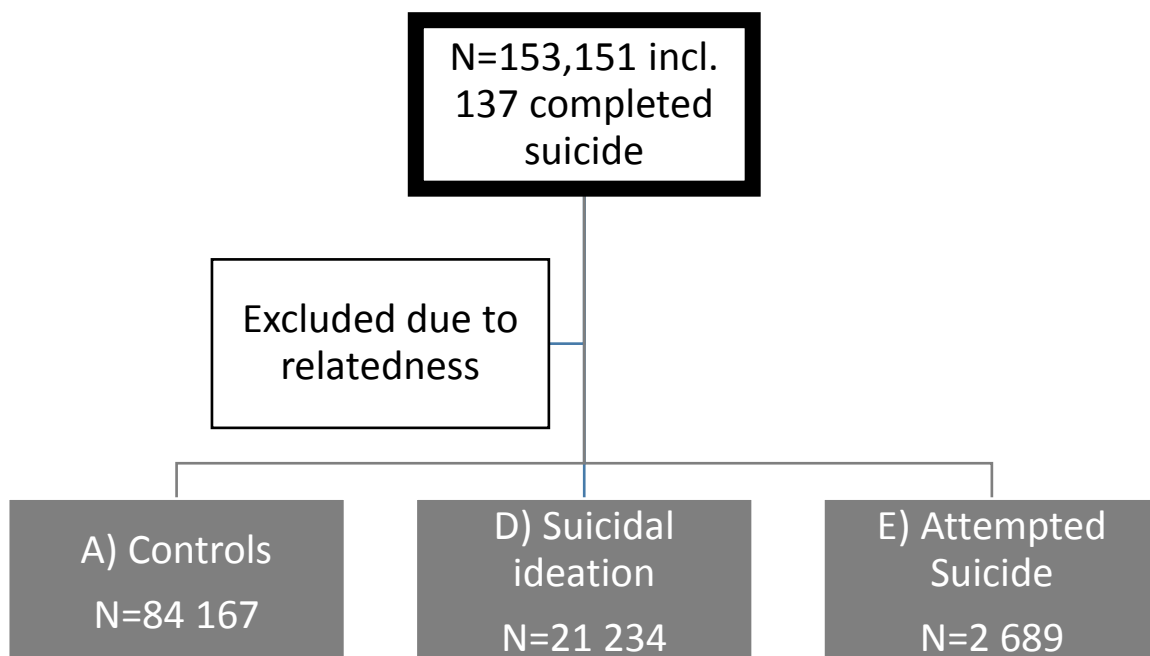

Supplement: Supplementary Fig. 2 — Flow chart of UK Biobank participants available for secondary analyses. The flow chart of participants is the same as Supplementary Fig. 1 up to the highlighted box. Relatedness exclusions were applied for A) the DSH GWAS considering the categories Controls, Contemplated self-harm and Actual self-ham and B) the SIA GWAS considering the categories Controls, Suicidal ideation and attempted suicide. [file mmc2.pdf]

Supplementary Figure 3

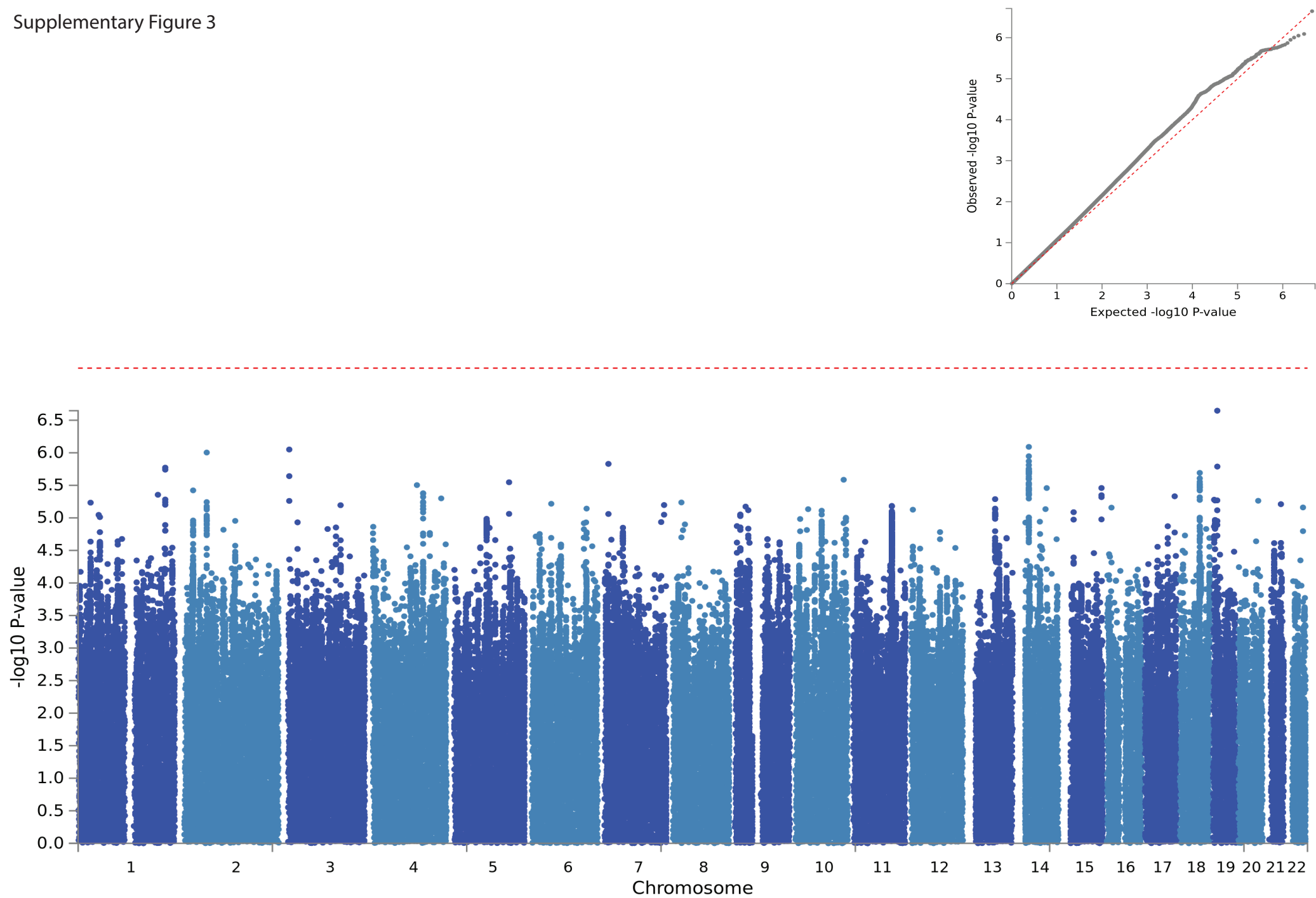

Supplement: Supplementary Fig. 3 — Manhattan plot of GWAS of ordinal suicidality in UK Biobank (N = 100,234), adjusted for age, sex, genotyping chip, population structure, psychiatric disorders and childhood sexual abuse. Dashed red line = genome wide significance threshold (p < 5 × 10^−5^). Inset: QQ plot for genome-wide association with DSH. Red line = theoretical distribution under the null hypothesis of no association. [file mmc3.pdf]

Supplementary Figure 4

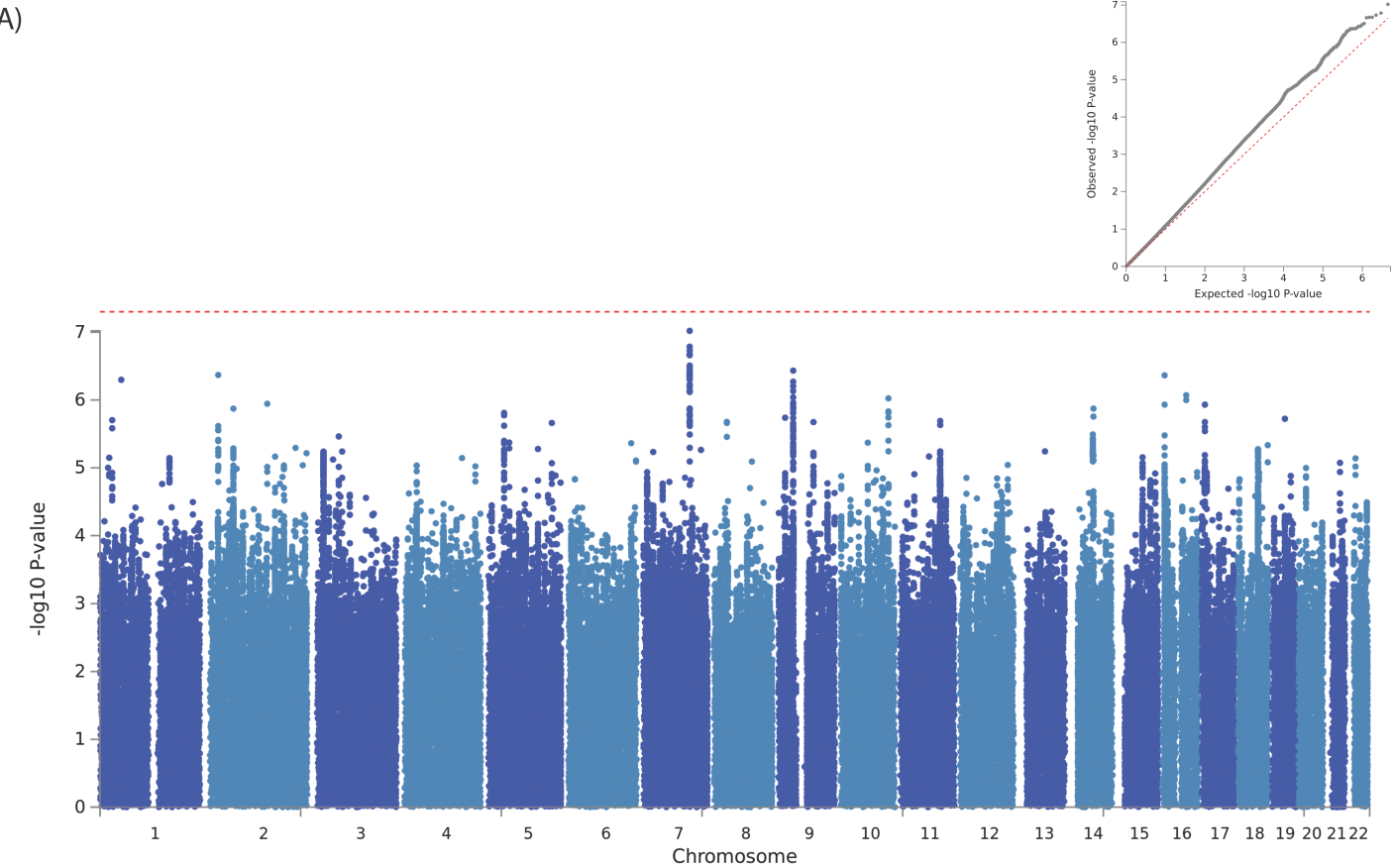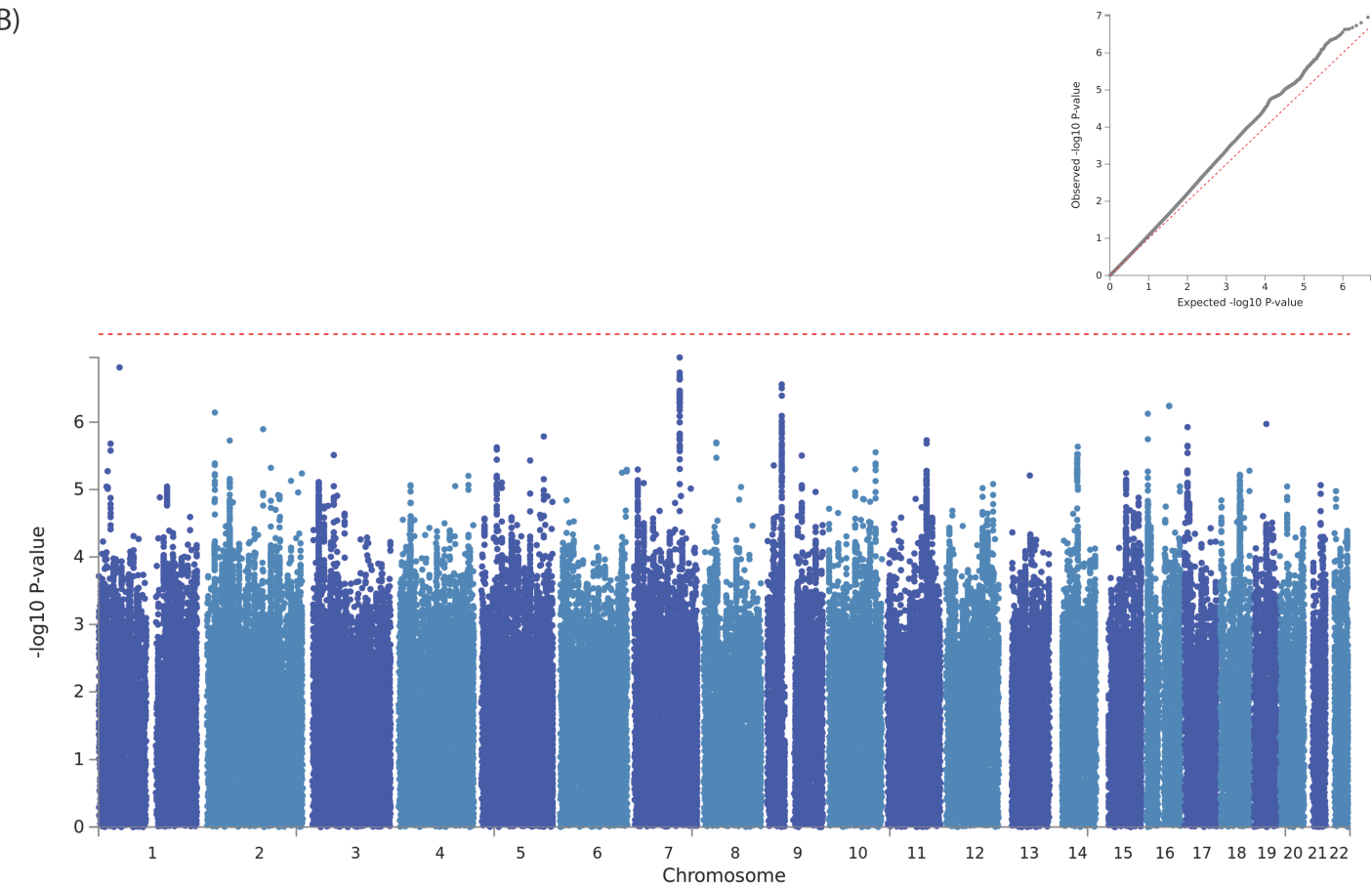

Supplement: Supplementary Fig. 4 — Manhattan plot of GWAS of ordinal DSH in UK Biobank (N = 100,234). Dashed red line = genome wide significance threshold (p < 5 × 10^−5^). Inset: QQ plot for genome-wide association with DSH. Red line = theoretical distribution under the null hypothesis of no association. [file mmc4.pdf]

Supplementary Figure 5

A)

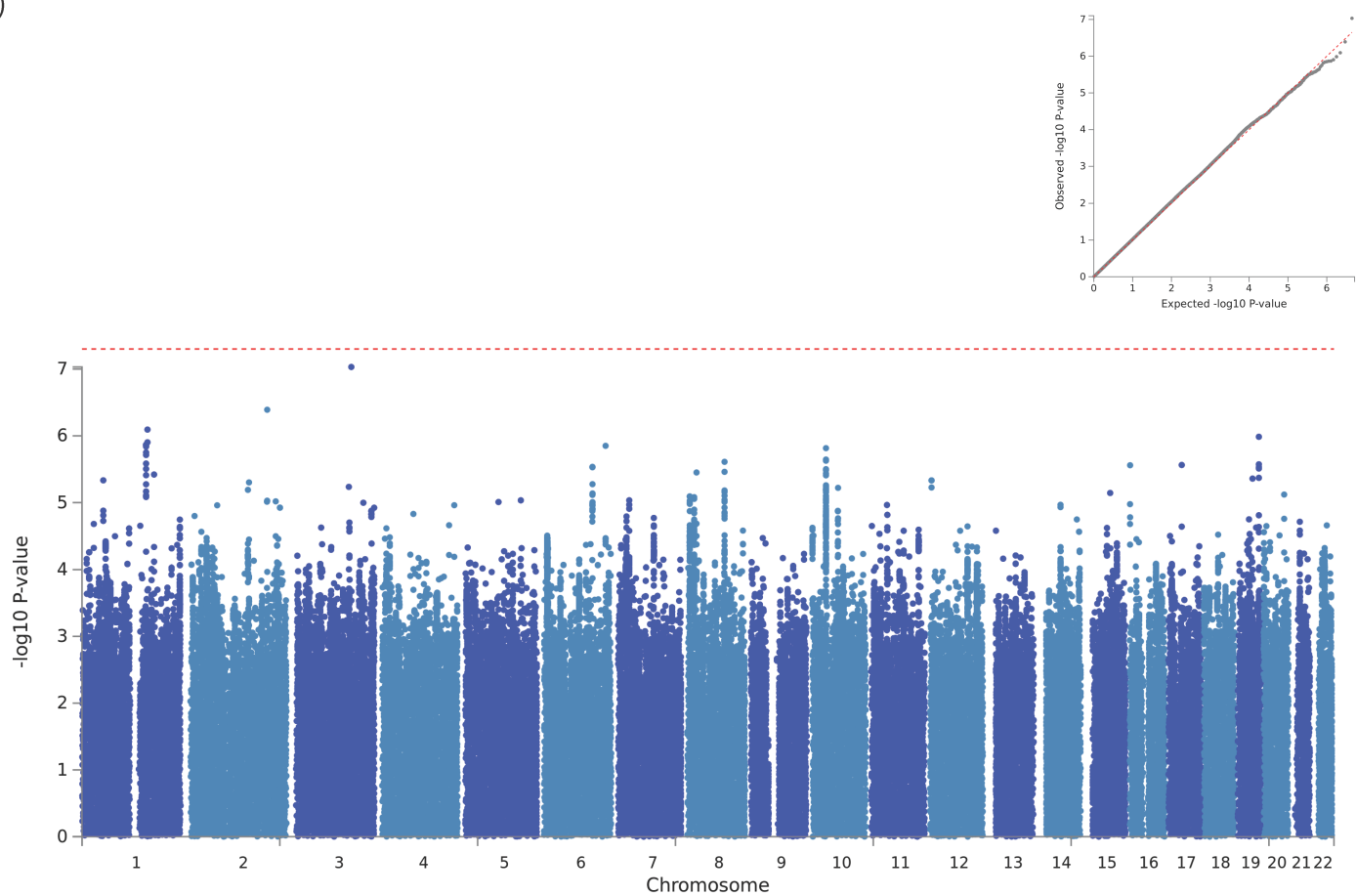

B)

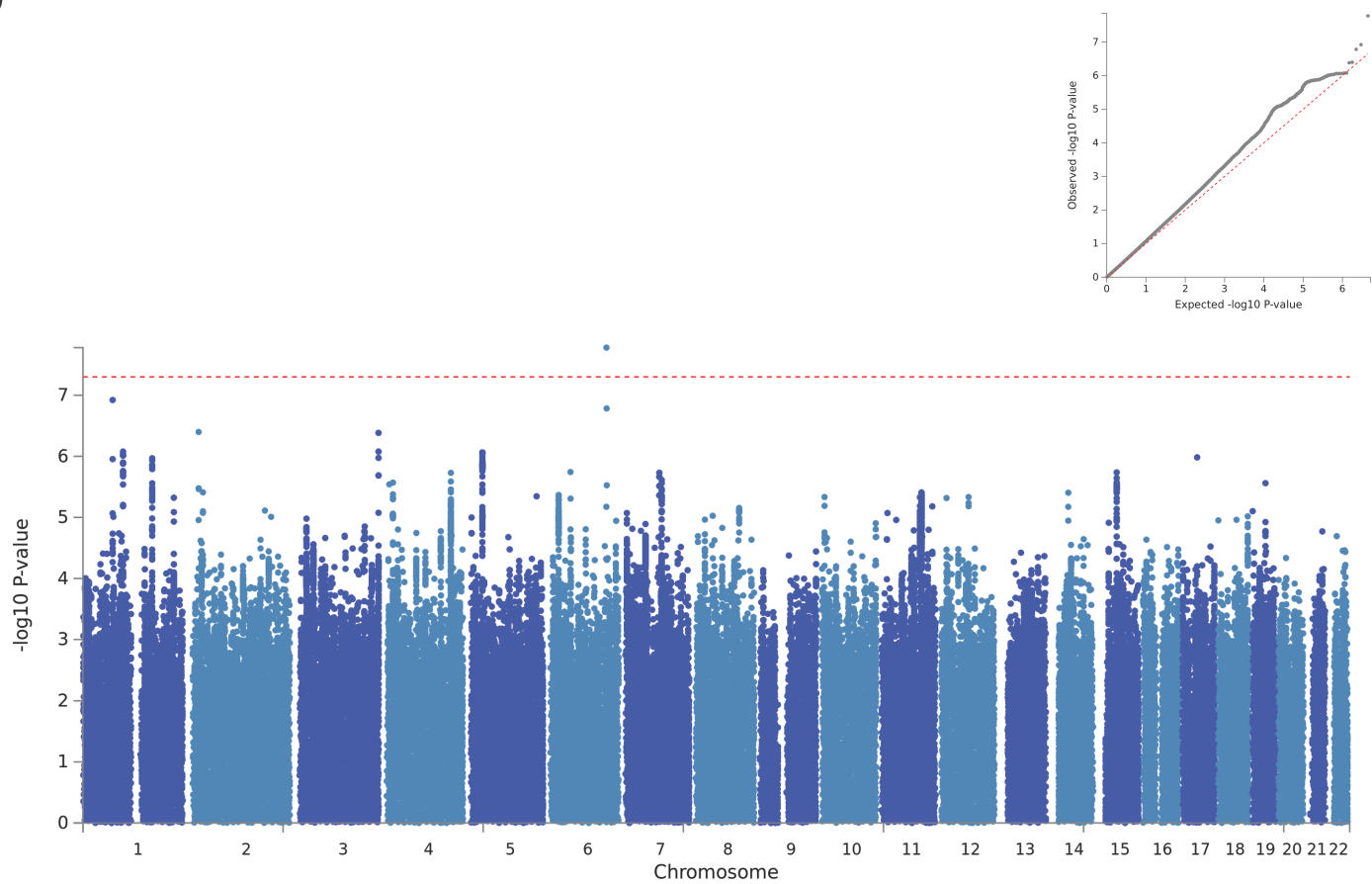

C)

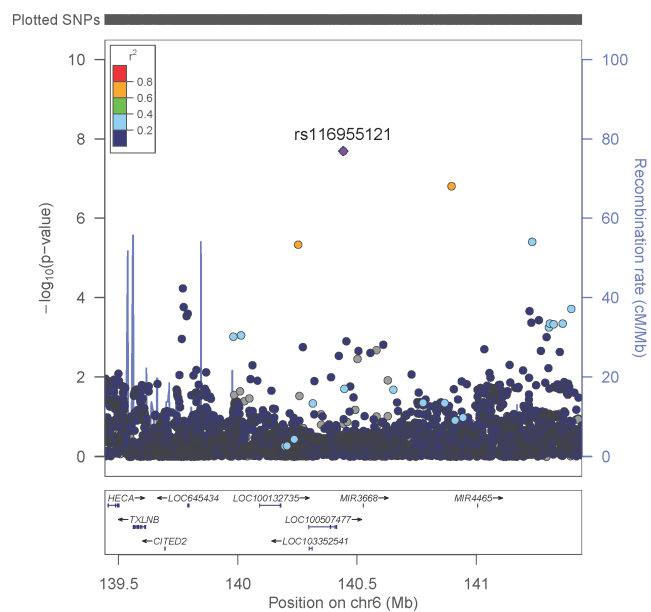

Supplement: Supplementary Fig. 5 — Manhattan plot of GWAS of ordinal SIA in UK Biobank (N = 108,090). Dashed red line = genome wide significance threshold (p < 5 × 10^−5^). Inset: QQ plot for genome-wide association with SIA. Red line = theoretical distribution under the null hypothesis of no association. [file mmc5.pdf]

Supplementary Figure 6

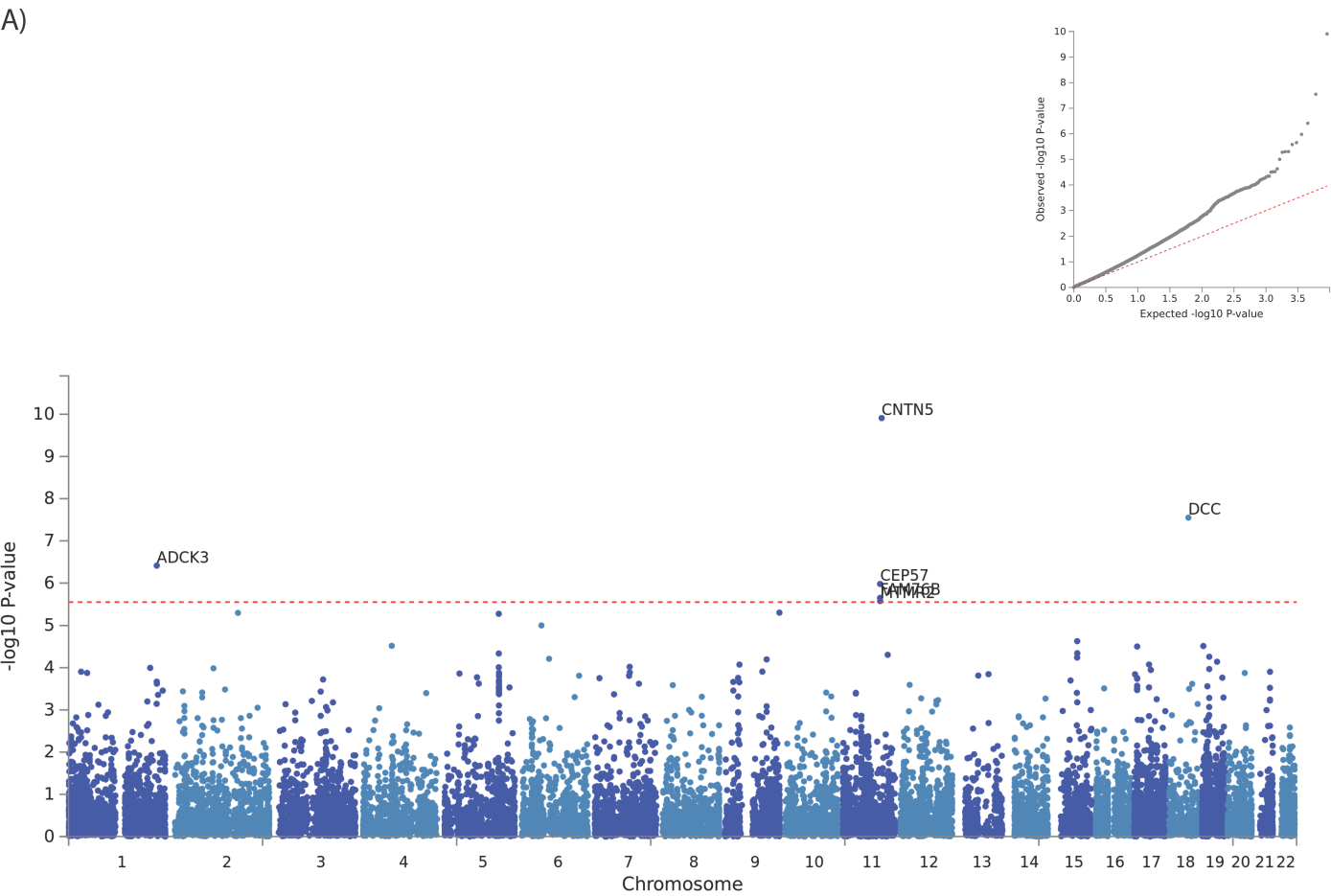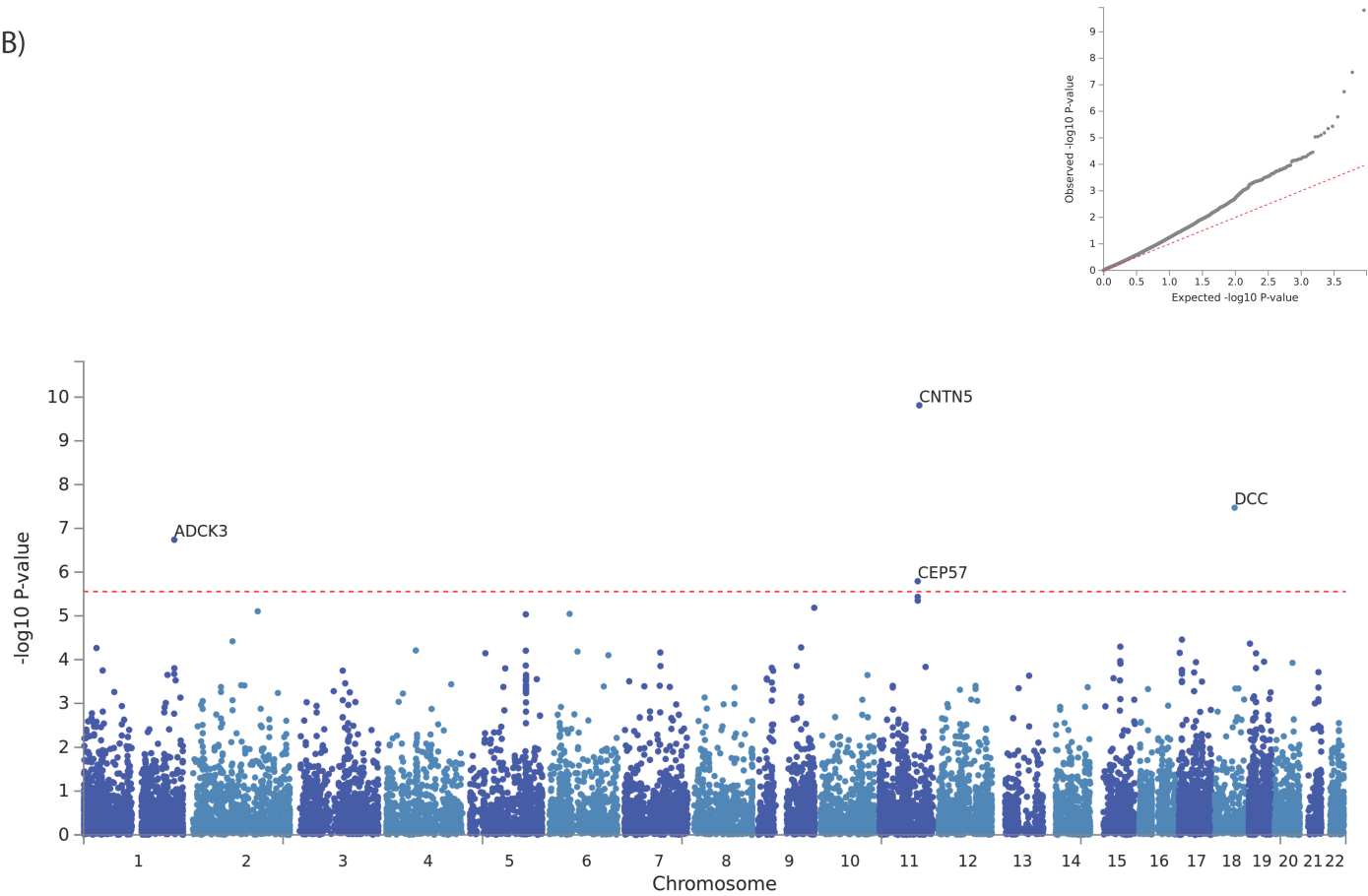

Supplement: Supplementary Fig. 6 — Manhattan plot of gene-based GWAS of ordinal suicide in UK Biobank (N = 122,935). Dashed red line = genome wide significance threshold (p < 5 × 10^−5^). Inset: QQ plot for genome-wide association with suicidality in UK Biobank. Red line = theoretical distribution under the null hypothesis of no association. [file mmc6.pdf]

Supplementary Figure 7

A)

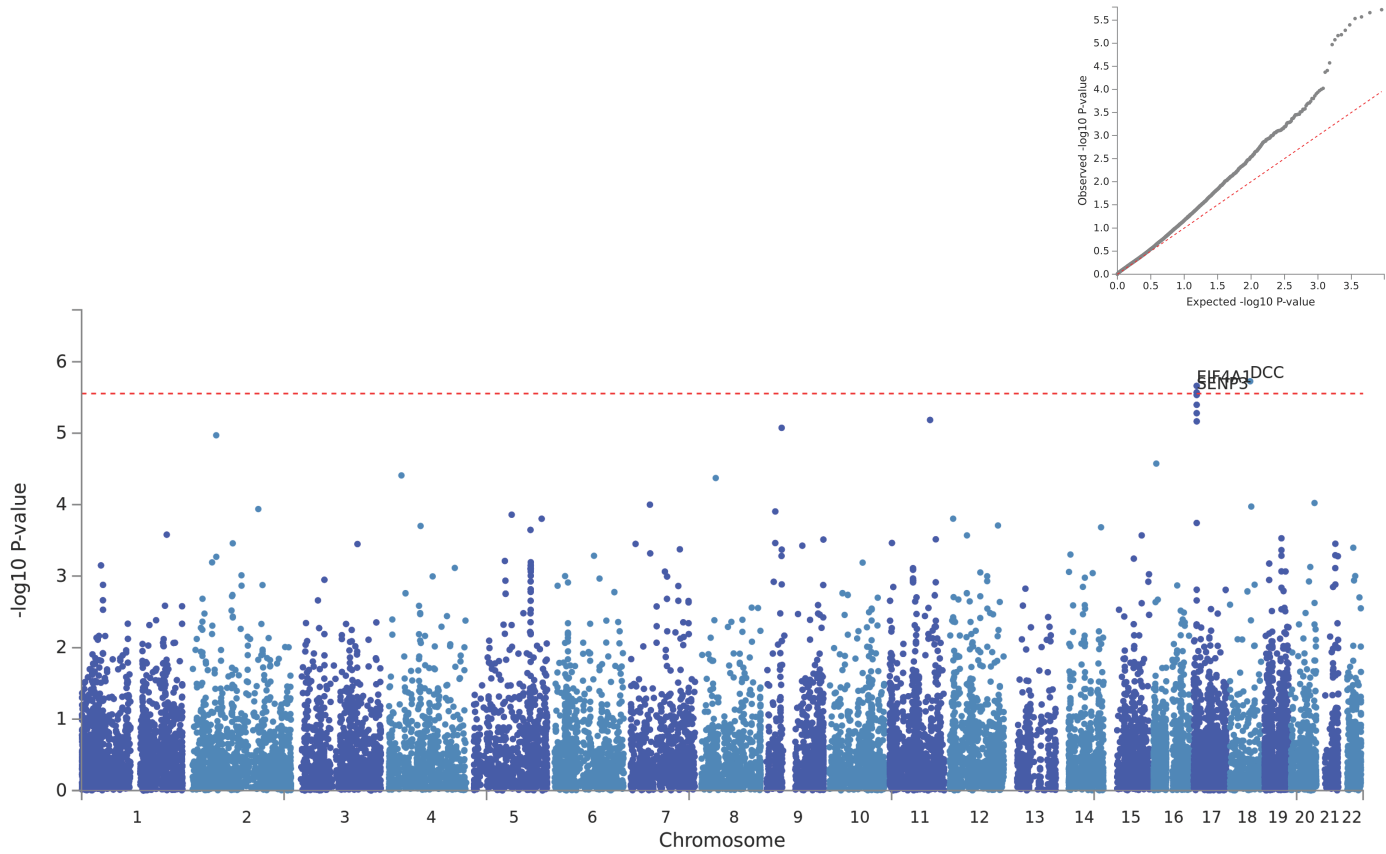

B)

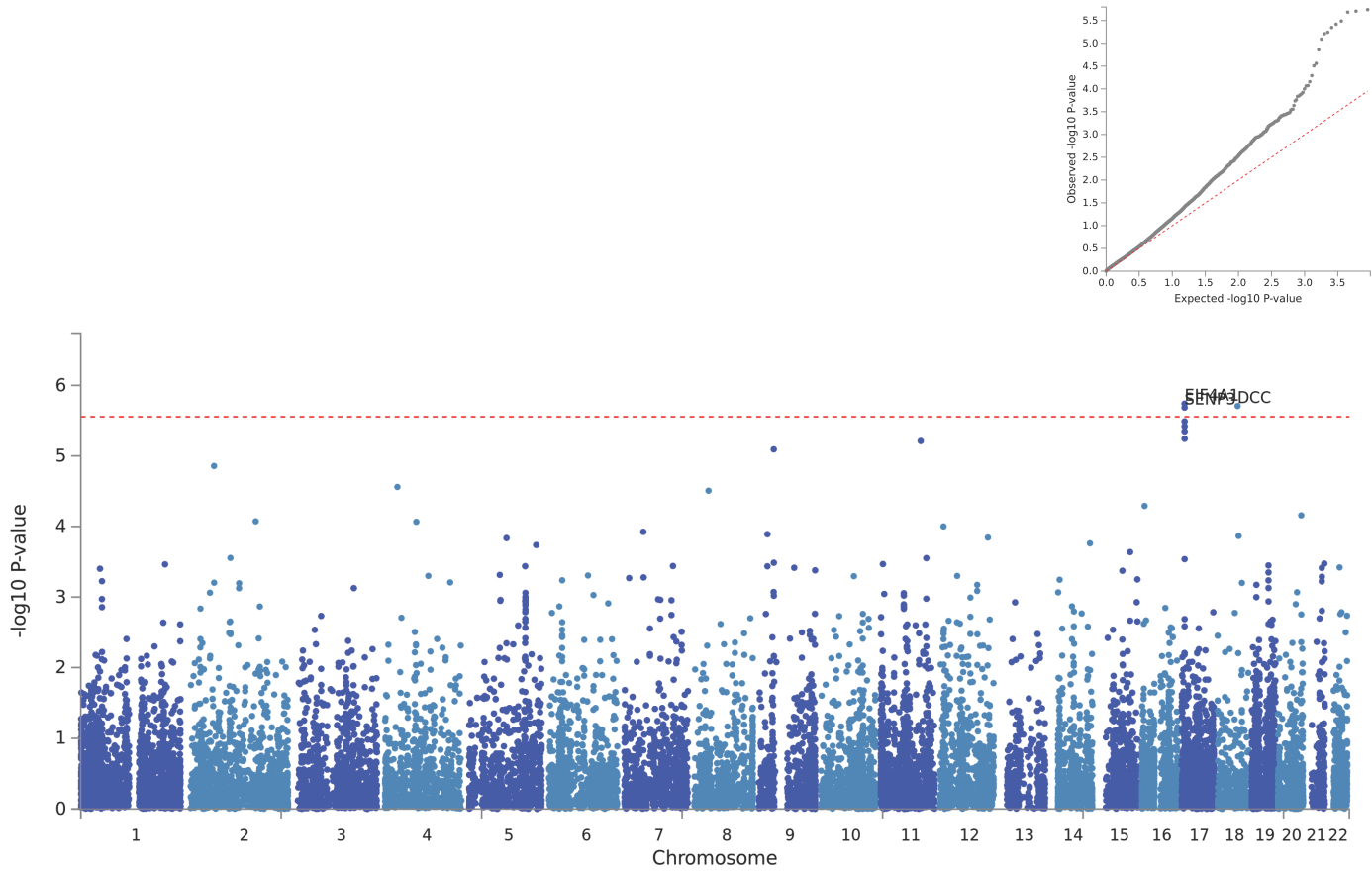

Supplement: Supplementary Fig. 7 — Manhattan plot of gene-based GWAS of ordinal DSH in UK Biobank (N = 100,234). Dashed red line = genome wide significance threshold (p < 5 × 10^−5^). Inset: QQ plot for genome-wide association with suicidality in UK Biobank. Red line = theoretical distribution under the null hypothesis of no association. [file mmc7.pdf]

Supplementary Figure 8

A)

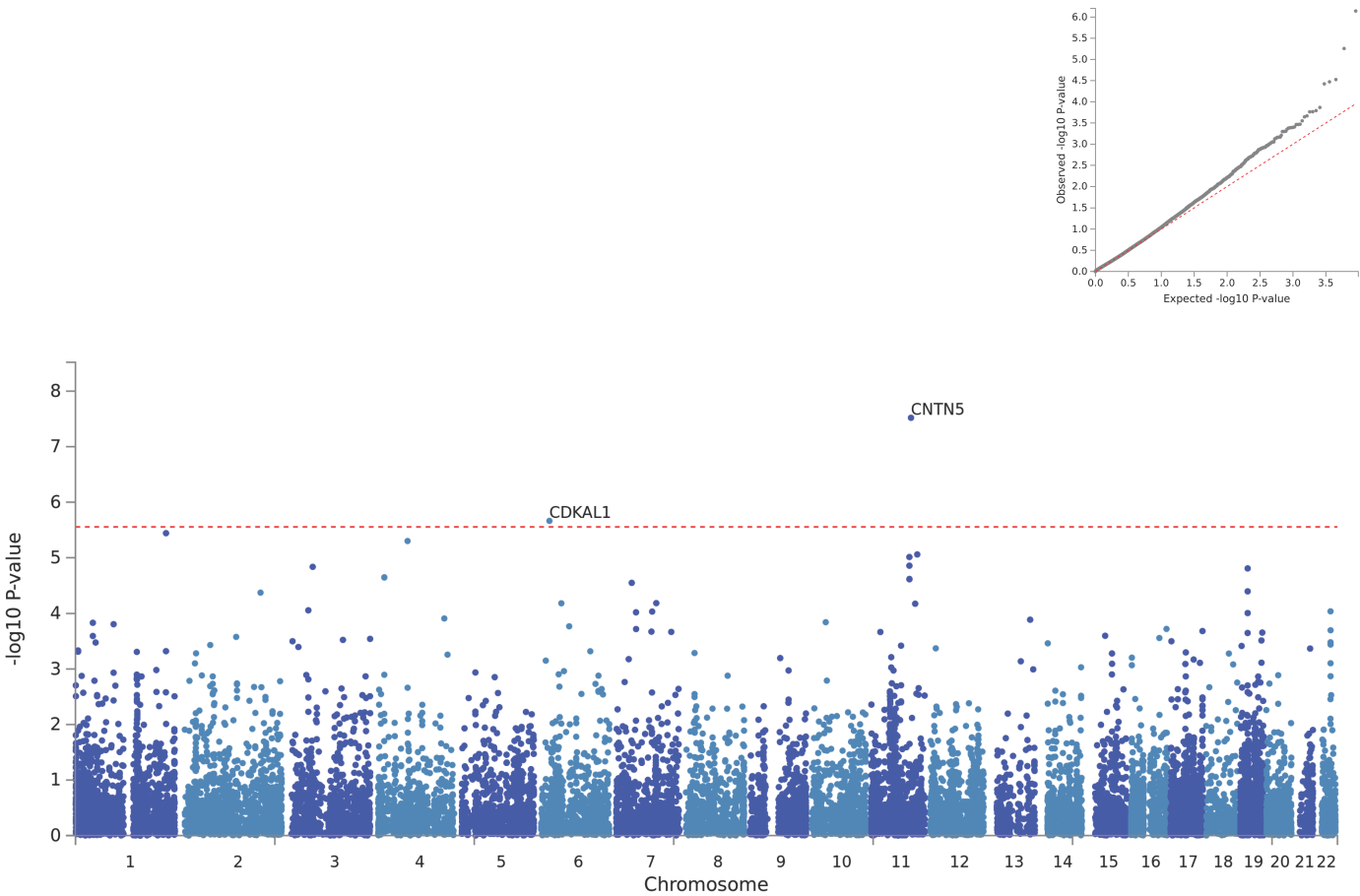

B)

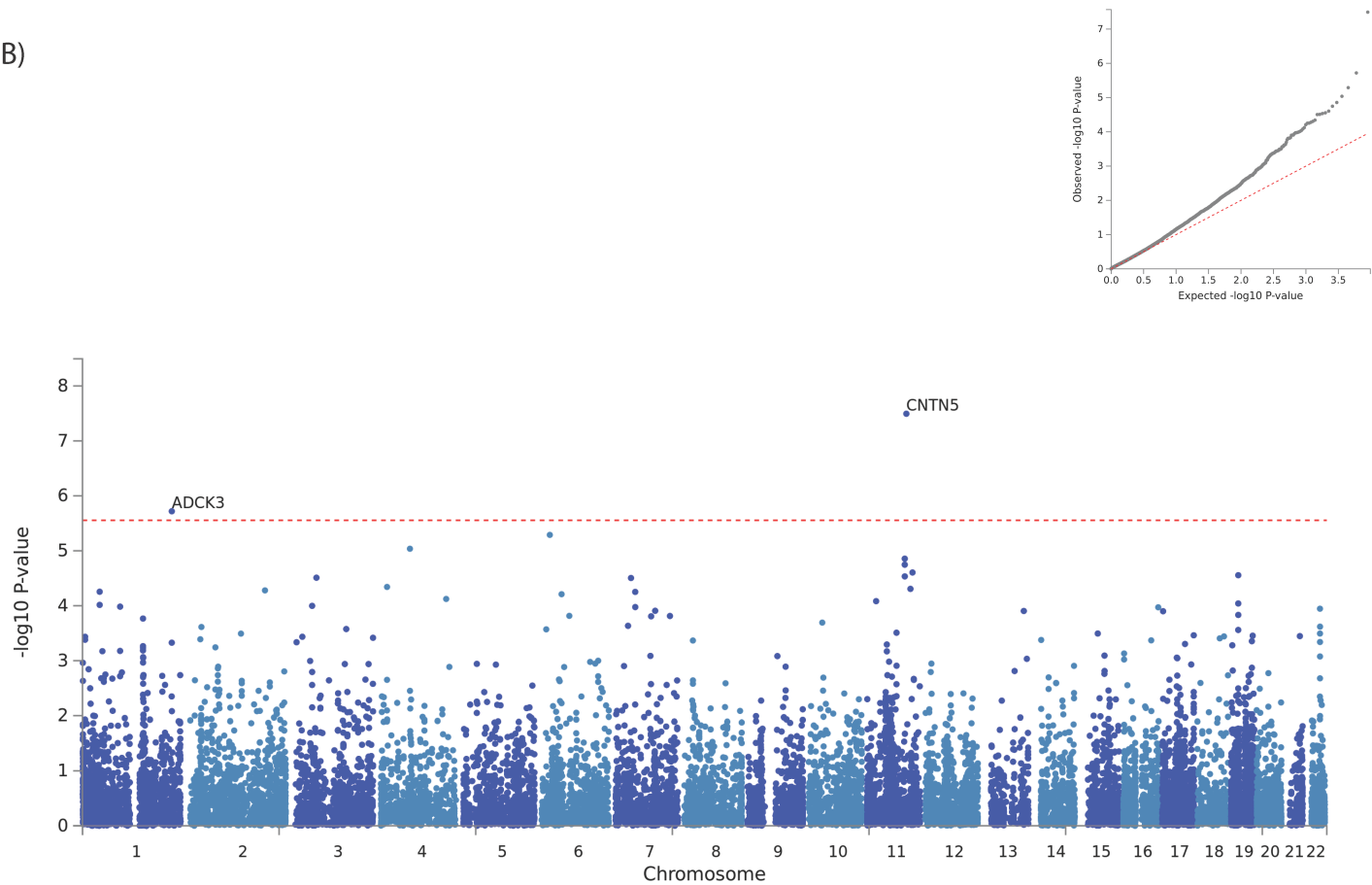

Supplement: Supplementary Fig. 8 — Manhattan plot of gene-based GWAS of ordinal SIA in UK Biobank (N = 108,090). Dashed red line = genome wide significance threshold (p < 5 × 10^−5^). Inset: QQ plot for genome-wide association with suicidality in UK Biobank. Red line = theoretical distribution under the null hypothesis of no association. [file mmc8.pdf]

Supplementary Figure 9

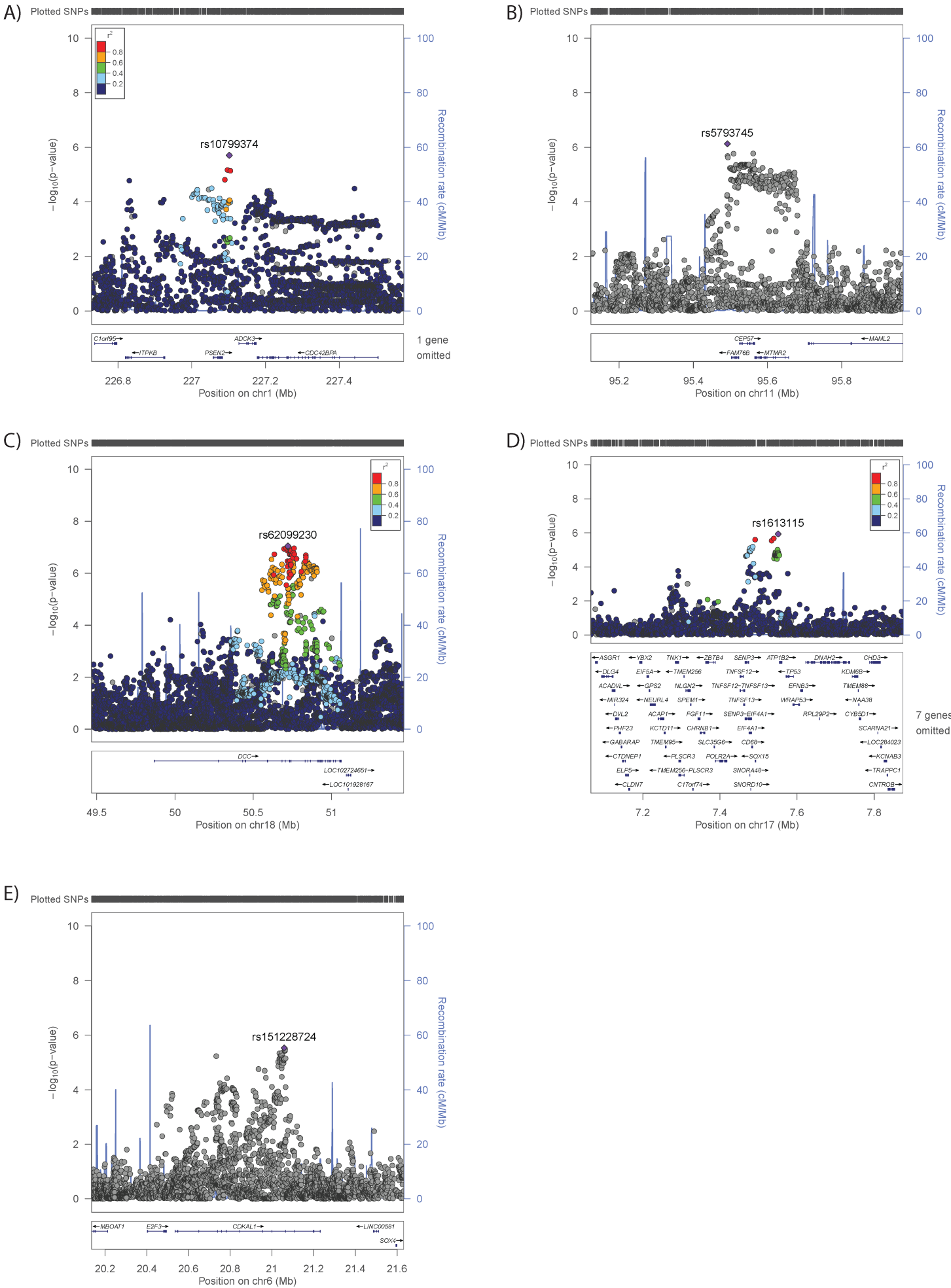

Supplement: Supplementary Fig. 9 — Regional plots for GWAS significant loci identified in the gene-based analyses. Highlighted genes for suicidality A) ADCK3/COQ8A on Chromosome 1, B) CEP57-FAM76B-MTMR2 on Chromosome 11, C) DCC on Chromosome 18, For DSH D) SENP3 on Chromosome 17 and for SIA E) CDKAL1 on Chromosome 6. SNPs (each point) are aligned according to position (X axis) and strength of association (Y Axis, left); Purple colouring indicates the index SNP, with r2 linkage disequilibrium with the index SNP being presented by colour as per the colour key; Rates of DNA recombination are presented as a pale blue line graph in the background (Y axis, right); Genes are presented by location (X axis) and direction of transcription (arrows). [file mmc9.pdf]
