# Supplementary material for: Identification of novel genome-wide associations for suicidality in UK Biobank, genetic correlation with psychiatric disorders and polygenic association with completed suicide
Source: eBioMedicine. 2019 Feb 8;41:517–25. doi: 10.1016/j.ebiom.2019.02.005 (PMC6442001; doi:10.1016/j.ebiom.2019.02.005)
Supplement: Supplementary Fig. 10 — Genotype-specific gene expression of the Chromosome 9 lead SNP, rs62535711 on transcripts of FRMPD1, MELK, TRMT10B, ZCCHC7 and GRHPR, (Supplementary Fig. 3 E–H) in cerebellar cortex (CRBL), frontal cortex (FCTX), hippocampus (HIPP), medulla (specifically inferior olivary nucleus, MEDU), occipital cortex (specifically primary visual cortex, OCTX), putamen (PUTM), substantia nigra (SNIG), thalamus (THAL), temporal cortex (TCTX) and intralobular white matter (WHMT). [file mmc10.pdf]

Supplementary Figure 10

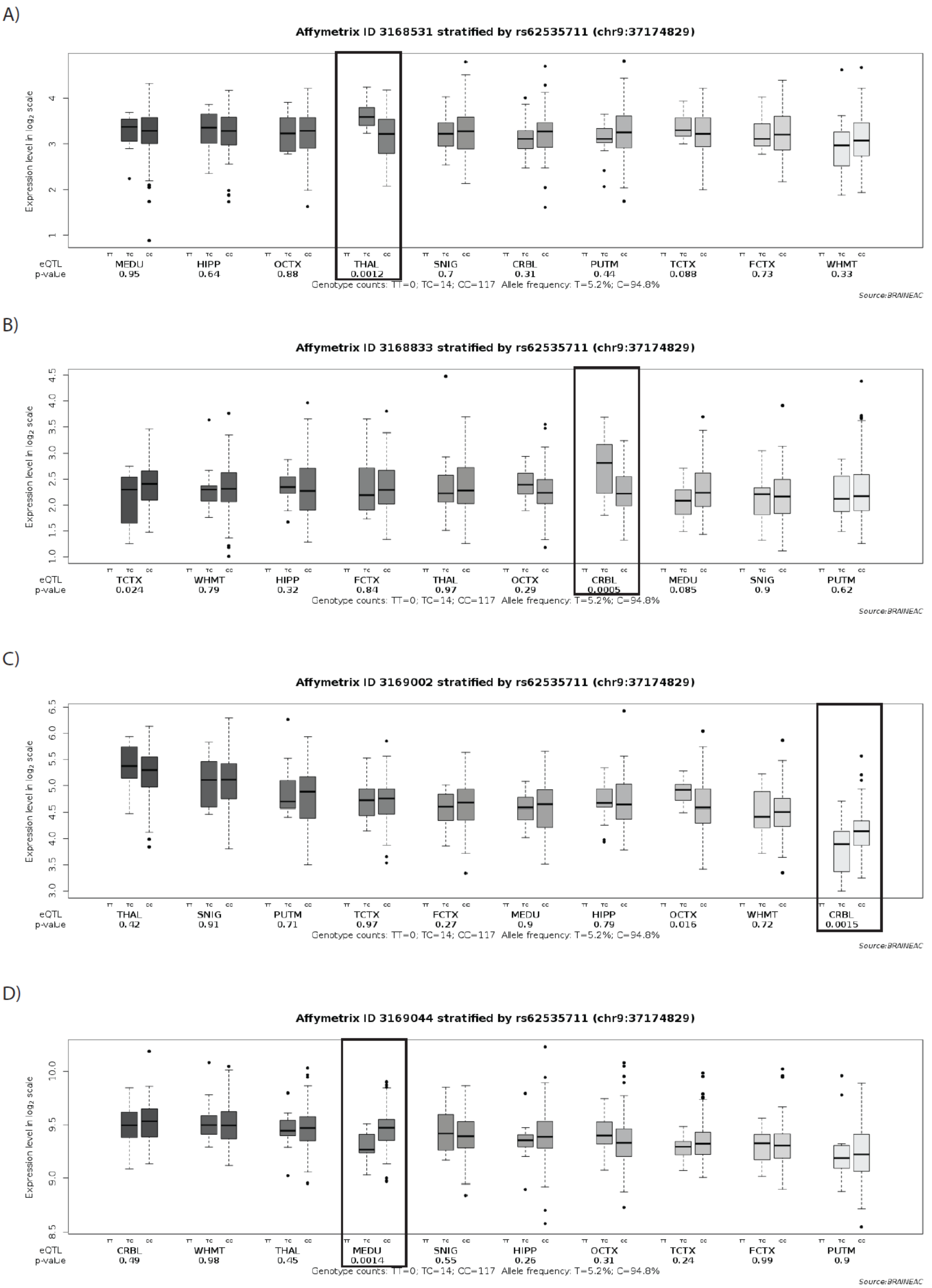

E)

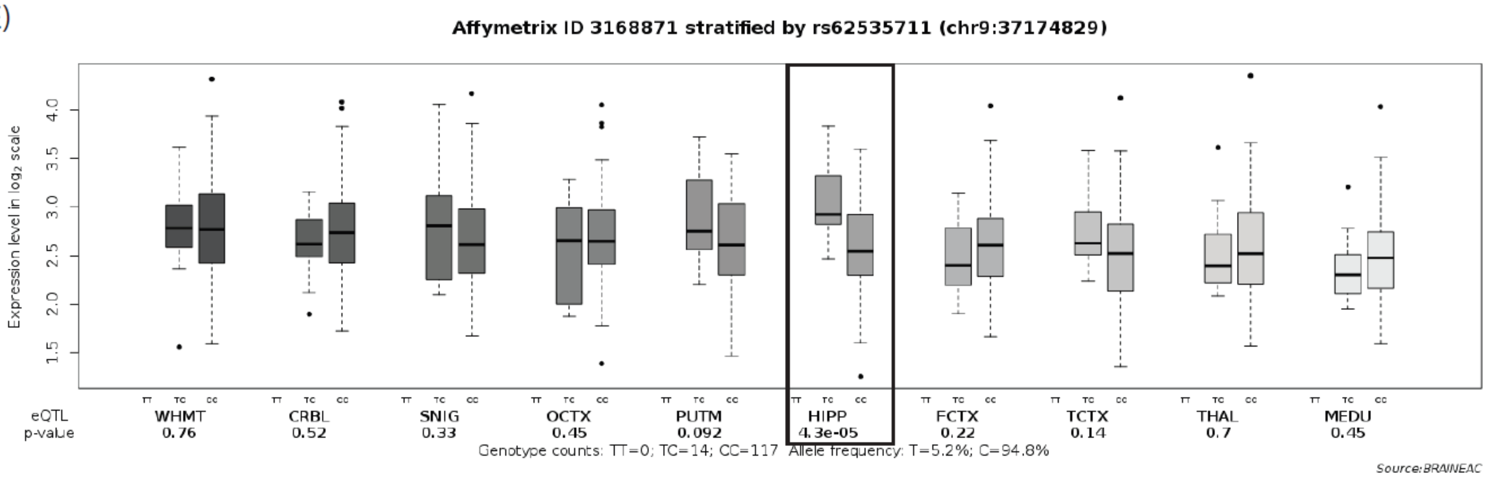
Affymetrix ID 3168871 stratified by rs62535711 (chr9:37174829)
Expression level in log₂ scale
eQTL
p-value
WHMT 0.76
CRBL 0.52
SNIG 0.33
OCTX 0.45
PUTM 0.092
HIPP 4.3e-05
FCTX 0.22
TCTX 0.14
THAL 0.7
MEDU 0.45
Genotype counts: TT=0; TC=14; CC=117 Allele frequency: T=5.2%; C=94.8%
Source:BRAINEAC

F)

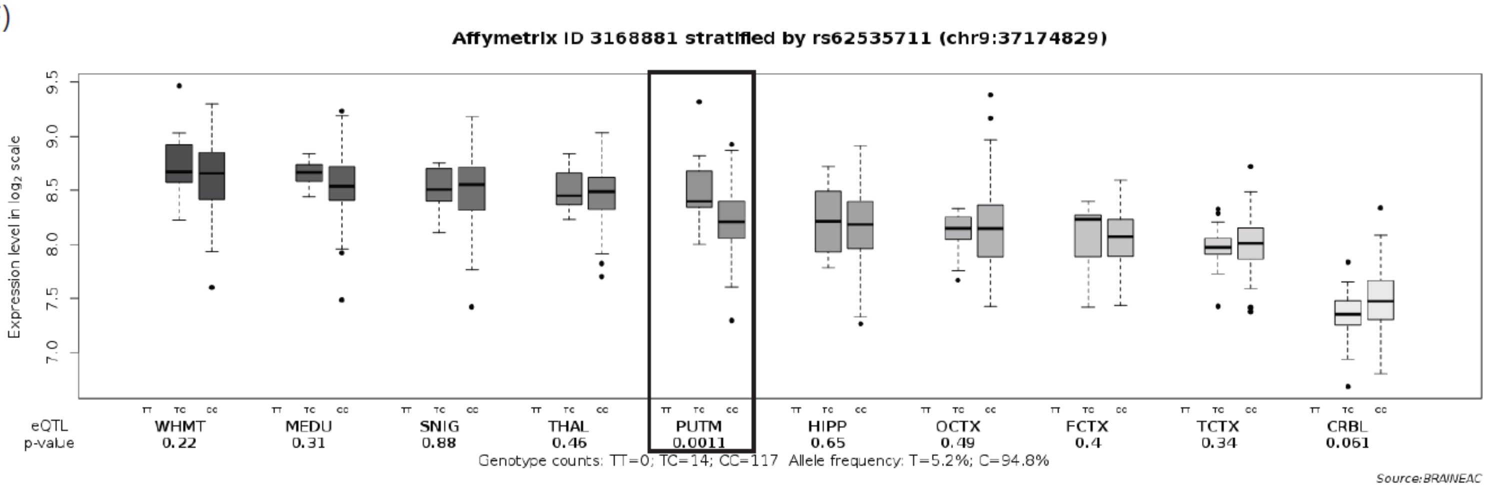
Affymetrix ID 3168881 stratified by rs62535711 (chr9:37174829)
Expression level in log₂ scale
eQTL
p-value
WHMT 0.22
MEDU 0.31
SNIG 0.88
THAL 0.46
PUTM 0.0011
HIPP 0.65
OCTX 0.49
FCTX 0.4
TCTX 0.34
CRBL 0.061
Genotype counts: TT=0; TC=14; CC=117 Allele frequency: T=5.2%; C=94.8%
Source:BRAINEAC

G)

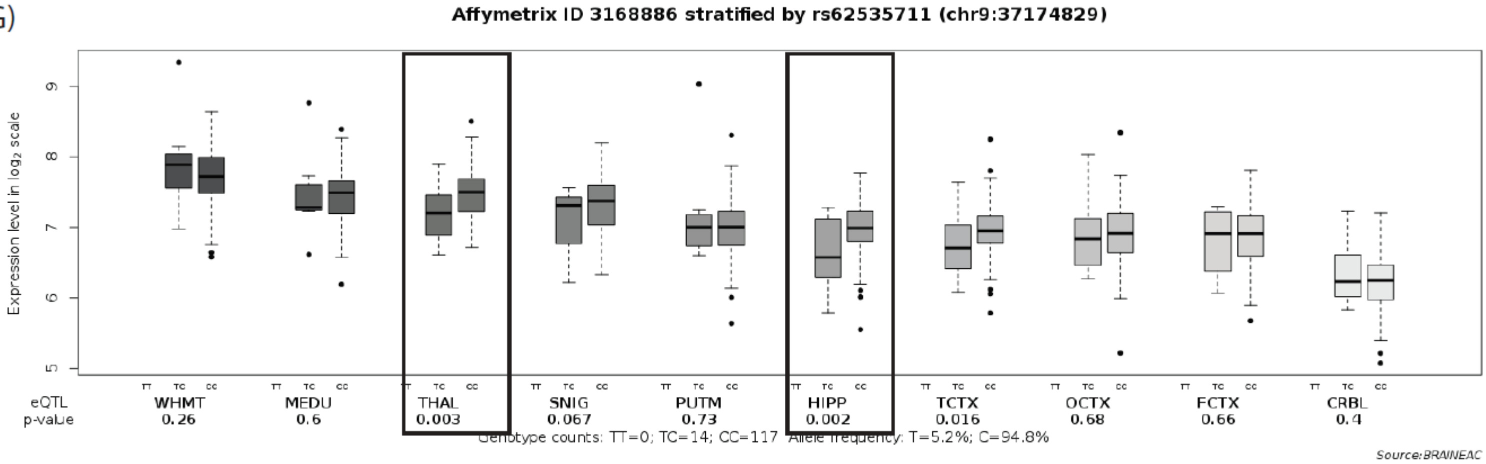
Affymetrix ID 3168886 stratified by rs62535711 (chr9:37174829)
Expression level in log₂ scale
eQTL
p-value
WHMT 0.26
MEDU 0.6
THAL 0.003
SNIG 0.067
PUTM 0.73
HIPP 0.002
TCTX 0.016
OCTX 0.68
FCTX 0.66
CRBL 0.4
Genotype counts: TT=0; TC=14; CC=117 Allele frequency: T=5.2%; C=94.8%
Source:BRAINEAC

H)

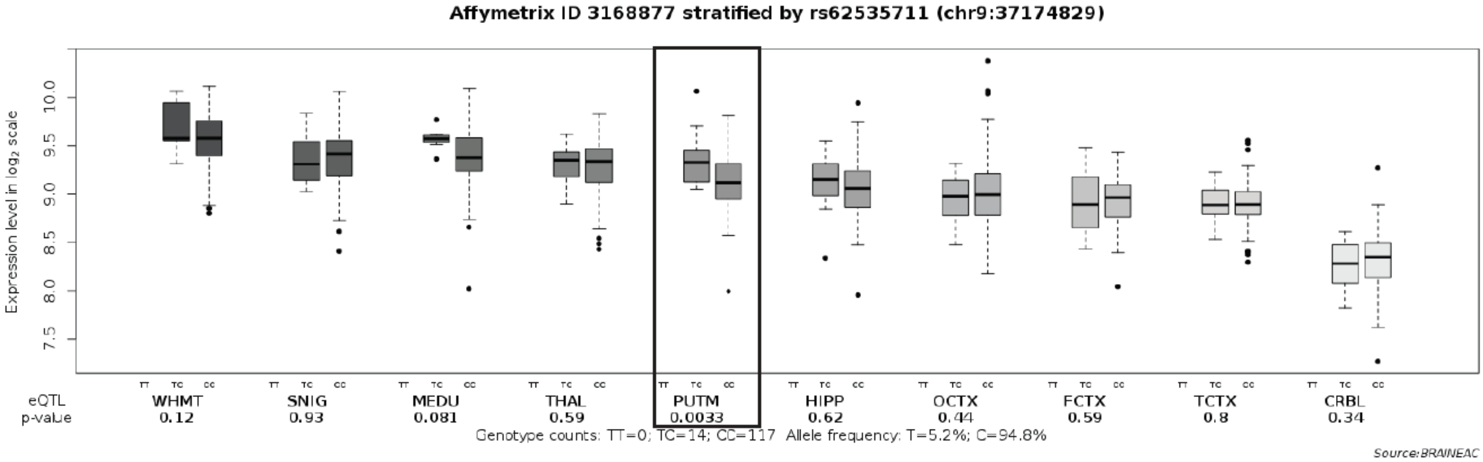
Affymetrix ID 3168877 stratified by rs62535711 (chr9:37174829)
Expression level in log₂ scale
eQTL
p-value
WHMT 0.12
SNIG 0.93
MEDU 0.081
THAL 0.59
PUTM 0.0033
HIPP 0.62
OCTX 0.44
FCTX 0.59
TCTX 0.8
CRBL 0.34
Genotype counts: TT=0; TC=14; CC=117 Allele frequency: T=5.2%; C=94.8%
Source:BRAINEAC
