# Supplementary material for: Identification of novel genome-wide associations for suicidality in UK Biobank, genetic correlation with psychiatric disorders and polygenic association with completed suicide
Source: eBioMedicine. 2019 Feb 8;41:517–25. doi: 10.1016/j.ebiom.2019.02.005 (PMC6442001; doi:10.1016/j.ebiom.2019.02.005)
Supplement: Supplementary Table1 — Cohort demographics of individuals included in the Ordinal suicidality GWAS and the completed suicide PRS analysis [file mmc11.docx]

| **Supplemental Table1: Cohort demographics of individuals included in the Ordinal suicidality GWAS and the completed suicide PRS analysis** | | | | | | | | | | | | | | |
| --- | --- | --- | --- | --- | --- | --- | --- | --- | --- | --- | --- | --- | --- | --- |
| Analysis | Ordinal Suicidality GWAS | | | | | | | | | | Completed Suicide PRS | | | |
| Suicidality category | 0: No Reported Suicidality | | 1: Thought life not worth living | | 2: Contemplated Self harm | | 4: Deliberately Self harmed | | 5: Attempted Suicide | | 0: No Reported Suicidality | | 6: Completed Suicide^a^ | |
|  | (n=83,557) | | (n=21,063) | | (n=13,038) | | (n=2,498) | | (n=2,666) | | (n=5,330) | | (n=137) | |
|  | n | % | n | % | n | % | n | % | n | % | n | % | n | % |
| Sex |  |  |  |  |  |  |  |  |  |  |  |  |  |  |
| Female | 44,057 | 52.7 | 12,781 | 60.7 | 8,299 | 63.7 | 1,757 | 70.3 | 1,804 | 67.7 | 2,926 | 54.9 | 37 | 27.0 |
| Male | 39,500 | 47.3 | 8,282 | 39.3 | 4,739 | 36.4 | 741 | 29.7 | 862 | 32.3 | 1,174 | 48.9 | 100 | 73.0 |
| Age range |  |  |  |  |  |  |  |  |  |  |  |  |  |  |
| 35/44 | 6,887 | 8.2 | 2,171 | 10.3 | 1,802 | 13.8 | 527 | 21.1 | 421 | 15.8 | 418 | 7.8 | 20 | 14.6 |
| 45/54 | 22,431 | 26.9 | 6,900 | 32.8 | 5,006 | 38.4 | 1,080 | 43.2 | 1,039 | 39.0 | 1,400 | 26.3 | 52 | 38.0 |
| 55/64 | 40,002 | 47.9 | 9,326 | 44.3 | 5,158 | 39.6 | 750 | 30.0 | 1,038 | 38.9 | 2,590 | 48.6 | 50 | 36.5 |
| 65/74 | 14,237 | 17.0 | 2,666 | 12.7 | 1,072 | 8.2 | 141 | 5.6 | 168 | 6.3 | 922 | 17.3 | 15 | 11.0 |
| Living arrangements | |  |  |  |  |  |  |  |  |  |  |  |  |  |
| Alone | 12,456 | 14.9 | 4,318 | 20.6 | 2,850 | 21.9 | 531 | 21.3 | 743 | 28.0 | 785 | 14.8 | 43 | 31.6 |
| With partner | 66,243 | 79.5 | 14,894 | 70.9 | 8,865 | 68.2 | 1,662 | 66.8 | 1,573 | 59.3 | 4,213 | 79.2 | 76 | 55.9 |
| Other | 4,678 | 5.6 | 1,788 | 8.5 | 1,282 | 9.9 | 297 | 11.9 | 338 | 12.7 | 321 | 6.0 | 17 | 12.5 |
| Area Deprivation |  |  |  |  |  |  |  |  |  |  |  |  |  |  |
| Least deprived | 21,116 | 25.3 | 4,667 | 22.2 | 2,701 | 20.8 | 452 | 18.1 | 392 | 14.7 | 1,293 | 24.3 | 20 | 14.6 |
| 4^th^ Quintile | 19,608 | 23.5 | 4,414 | 21.0 | 2,546 | 19.6 | 442 | 17.7 | 416 | 15.6 | 1,279 | 24.0 | 26 | 19.0 |
| 3^rd^ Quintile | 17,829 | 21.4 | 4,464 | 21.2 | 2,626 | 20.2 | 503 | 20.2 | 509 | 19.1 | 1,154 | 21.7 | 19 | 13.9 |
| 2^nd^ Quintile | 14,937 | 17.9 | 4,181 | 19.9 | 2,785 | 21.4 | 585 | 23.4 | 636 | 23.9 | 982 | 18.4 | 34 | 24.8 |
| Most deprived | 9,972 | 12.0 | 3,310 | 15.7 | 2,360 | 18.1 | 514 | 20.6 | 708 | 26.6 | 618 | 11.6 | 38 | 27.7 |
| Parental Depression | |  |  |  |  |  |  |  |  |  |  |  |  |  |
| Neither parent | 69,837 | 91.5 | 16,505 | 87.2 | 9,754 | 83.0 | 1,769 | 79.3 | 1,761 | 77.3 | 4,518 | 92.1 | 89 | 77.4 |
| At least one parent | 6,486 | 8.5 | 2,434 | 12.9 | 2,001 | 17.0 | 461 | 20.7 | 516 | 22.7 | 387 | 7.9 | 26 | 22.6 |
| Chronic Pain |  |  |  |  |  |  |  |  |  |  |  |  |  |  |
| Free of pain | 53,529 | 64.1 | 11,658 | 55.4 | 6,637 | 50.9 | 1,234 | 49.4 | 1,157 | 43.4 | 3,417 | 64.2 | 83 | 60.6 |
| One or more sites | 29,983 | 35.9 | 9,389 | 44.6 | 6,398 | 49.1 | 1,262 | 50.6 | 1,508 | 56.6 | 1,907 | 35.8 | 54 | 39.4 |
| Smoking |  |  |  |  |  |  |  |  |  |  |  |  |  |  |
| Never | 49,886 | 59.8 | 11,868 | 56.5 | 6,932 | 53.3 | 1,204 | 48.3 | 1,103 | 41.5 | 3,201 | 60.2 | 63 | 46.0 |
| Previous | 28,650 | 34.4 | 7,467 | 35.5 | 4,881 | 37.5 | 973 | 39.0 | 1,128 | 42.4 | 1,839 | 34.6 | 42 | 30.7 |
| Current | 4,861 | 5.8 | 1,686 | 8.0 | 1,203 | 9.2 | 317 | 12.7 | 429 | 16.1 | 282 | 5.3 | 32 | 23.4 |
| Alcohol use |  |  |  |  |  |  |  |  |  |  |  |  |  |  |
| Daily/almost daily | 20,319 | 24.3 | 5,002 | 23.8 | 2,874 | 22.1 | 541 | 21.7 | 562 | 21.1 | 1,271 | 23.9 | 37 | 23.9 |
| Regular | 44,393 | 53.1 | 10,536 | 50.0 | 6,330 | 48.6 | 1,196 | 47.9 | 1,133 | 42.6 | 2,847 | 53.4 | 71 | 53.4 |
| Occasional | 15,005 | 18.0 | 4,397 | 20.9 | 3,014 | 23.1 | 577 | 23.1 | 694 | 26.1 | 947 | 17.8 | 19 | 17.7 |
| Former | 1,764 | 2.1 | 633 | 3.0 | 514 | 4.0 | 133 | 5.3 | 207 | 7.8 | 110 | 2.1 | 8 | 2.2 |
| Never | 2,053 | 2.5 | 486 | 2.3 | 297 | 2.3 | 50 | 2.0 | 65 | 2.4 | 154 | 2.9 | 2 | 2.9 |
| Life-time MDD |  |  |  |  |  |  |  |  |  |  |  |  |  |  |
| No | 62,153 | 85.1 | 7,952 | 49.9 | 3,078 | 30.3 | 603 | 30.4 | 317 | 14.9 | 4,045 | 86.3 | na | na |
| Yes | 10,876 | 14.9 | 7,996 | 50.1 | 7,088 | 69.7 | 1,380 | 69.6 | 1,818 | 85.2 | 640 | 13.7 | na | na |
| Childhood trauma |  |  |  |  |  |  |  |  |  |  |  |  |  |  |
| None | 50,494 | 61.0 | 9,474 | 45.4 | 4,751 | 36.7 | 760 | 30.6 | 639 | 24.1 | 3,287 | 62.3 | na | na |
| Any | 32,258 | 39.0 | 11,403 | 54.6 | 8,186 | 63.3 | 1,722 | 69.4 | 2,009 | 75.9 | 1,992 | 37.7 | na | na |
| *MDD = major depressive disorder; n/a = not assessed* | | | | | |  |  |  |  |  |  |  |  |  |
