# Supplementary material for: Identification of novel genome-wide associations for suicidality in UK Biobank, genetic correlation with psychiatric disorders and polygenic association with completed suicide
Source: eBioMedicine. 2019 Feb 8;41:517–25. doi: 10.1016/j.ebiom.2019.02.005 (PMC6442001; doi:10.1016/j.ebiom.2019.02.005)
Supplement: Supplementary Table 2 — All SNPs associated with ordinal suicidality at GWAS significance [file mmc12.docx]

| **Supplemental Table 2: All SNPs associated with ordinal suicidality at GWAS significance** | | | | | | | | | |  |
| --- | --- | --- | --- | --- | --- | --- | --- | --- | --- | --- |
|  |  |  |  |  |  |  | basic | | adjusted for psychiatric disorders | |
| Analysis | SNP | CHR | BP | A1 | A2 | A1F^a^ | BETA | P | BETA | P |
| Ordinal suicidality | rs62535709 | 9 | 37166189 | T | C | 0.05 | 0.101 | 3.89E-08 | 0.099 | 8.67E-08 |
|  | **rs62535711** | 9 | 37174829 | T | C | 0.06 | 0.105 | 1.29E-08 | 0.103 | 3.12E-08 |
|  | rs635715 | 11 | 99495141 | G | T | 0.33 | 0.051 | 3.48E-08 | 0.052 | 1.97E-08 |
|  | rs639161 | 11 | 99495147 | G | A | 0.33 | 0.050 | 4.30E-08 | 0.051 | 2.51E-08 |
|  | rs637006 | 11 | 99495383 | A | G | 0.33 | 0.051 | 3.37E-08 | 0.052 | 1.88E-08 |
|  | rs637745 | 11 | 99495452 | C | G | 0.33 | 0.051 | 2.99E-08 | 0.052 | 1.67E-08 |
|  | rs637387 | 11 | 99495489 | T | C | 0.33 | 0.051 | 2.93E-08 | 0.052 | 1.64E-08 |
|  | rs1790263 | 11 | 99495583 | T | G | 0.33 | 0.051 | 3.10E-08 | 0.052 | 1.73E-08 |
|  | rs1790262 | 11 | 99495651 | T | A | 0.33 | 0.051 | 2.99E-08 | 0.052 | 1.67E-08 |
|  | rs636504 | 11 | 99495693 | G | A | 0.33 | 0.050 | 4.13E-08 | 0.051 | 2.32E-08 |
|  | rs636073 | 11 | 99495789 | C | T | 0.33 | 0.051 | 3.24E-08 | 0.052 | 1.80E-08 |
|  | rs651048 | 11 | 99496234 | C | T | 0.33 | 0.051 | 3.44E-08 | 0.052 | 1.92E-08 |
|  | rs651446 | 11 | 99496269 | A | G | 0.33 | 0.051 | 3.56E-08 | 0.052 | 2.00E-08 |
|  | rs651447 | 11 | 99496271 | C | T | 0.33 | 0.051 | 3.25E-08 | 0.052 | 1.87E-08 |
|  | rs651496 | 11 | 99496308 | A | G | 0.33 | 0.051 | 3.61E-08 | 0.052 | 2.02E-08 |
|  | rs1790261 | 11 | 99496359 | C | T | 0.33 | 0.051 | 3.32E-08 | 0.052 | 1.86E-08 |
|  | rs1690819 | 11 | 99496535 | A | G | 0.33 | 0.051 | 3.01E-08 | 0.052 | 1.67E-08 |
|  | rs1690818 | 11 | 99496554 | C | T | 0.33 | 0.051 | 2.78E-08 | 0.052 | 1.55E-08 |
|  | rs1690817 | 11 | 99496812 | A | G | 0.33 | 0.051 | 2.78E-08 | 0.052 | 1.55E-08 |
|  | rs1690816 | 11 | 99496849 | C | T | 0.33 | 0.051 | 2.78E-08 | 0.052 | 1.55E-08 |
|  | rs1003122 | 11 | 99496892 | A | G | 0.33 | 0.051 | 2.88E-08 | 0.052 | 1.60E-08 |
|  | rs1690814 | 11 | 99497012 | A | G | 0.33 | 0.051 | 2.91E-08 | 0.052 | 1.61E-08 |
|  | rs1790260 | 11 | 99497028 | C | A | 0.33 | 0.051 | 3.05E-08 | 0.052 | 1.69E-08 |
|  | rs609750 | 11 | 99497099 | G | T | 0.33 | 0.051 | 2.98E-08 | 0.052 | 1.66E-08 |
|  | rs646067 | 11 | 99497122 | T | C | 0.33 | 0.051 | 3.18E-08 | 0.052 | 1.76E-08 |
|  | rs646017 | 11 | 99497150 | T | G | 0.33 | 0.051 | 3.09E-08 | 0.052 | 1.72E-08 |
|  | rs634379 | 11 | 99497472 | C | T | 0.33 | 0.051 | 2.94E-08 | 0.052 | 1.64E-08 |
|  | rs634011 | 11 | 99497529 | C | T | 0.33 | 0.051 | 3.27E-08 | 0.052 | 1.81E-08 |
|  | 11:99500407_TTAC_T | 11 | 99500407 | TTAC | T | 0.33 | 0.051 | 3.89E-08 | 0.052 | 2.16E-08 |
|  | rs112677841 | 11 | 99507624 | A | G | 0.35 | 0.051 | 4.76E-08 | 0.052 | 2.81E-08 |
|  | rs111369127 | 11 | 99507634 | T | C | 0.35 | 0.051 | 4.76E-08 | 0.052 | 2.81E-08 |
|  | **rs598046** | 11 | 99516468 | T | G | 0.32 | 0.053 | 1.07E-08 | 0.054 | 5.18E-09 |
|  | rs7122777 | 11 | 99516476 | T | C | 0.32 | 0.053 | 1.13E-08 | 0.054 | 5.31E-09 |
|  | rs61910885 | 11 | 99516507 | C | G | 0.33 | 0.051 | 3.72E-08 | 0.053 | 1.65E-08 |
|  | rs61910886 | 11 | 99516510 | T | C | 0.33 | 0.051 | 3.72E-08 | 0.053 | 1.65E-08 |
|  | rs61910887 | 11 | 99516511 | G | A | 0.33 | 0.051 | 3.72E-08 | 0.053 | 1.65E-08 |
|  | **rs7989250** | 13 | 64900801 | A | C | 0.32 | -0.052 | 3.49E-08 | -0.051 | 4.31E-08 |
| SIA | **rs116955121** | 6 | 1.4E+08 | A | G | 0.02 |  |  | 0.199 | 1.66E-08 |
