# Supplementary material for: Identification of novel genome-wide associations for suicidality in UK Biobank, genetic correlation with psychiatric disorders and polygenic association with completed suicide
Source: eBioMedicine. 2019 Feb 8;41:517–25. doi: 10.1016/j.ebiom.2019.02.005 (PMC6442001; doi:10.1016/j.ebiom.2019.02.005)
Supplement: Supplementary Table 3 — Allele frequencies of lead SNPs by suicidality category [file mmc13.docx]

| **Supplemental Table 3: Allele frequencies of lead SNPs by suicidality category** | | | | | | |  |  |  |  |
| --- | --- | --- | --- | --- | --- | --- | --- | --- | --- | --- |
|  |  |  |  | ordinal GWAS | | | | | PRS | |
| SNP | CHR | POS | A1 | 0: No Reported Suicidality | 1: Thought life not worth living | 2: Contemplated Self harm | 4: Deliberately Self harmed | 5: Attempted Suicide | 0: No Reported Suicidality | 6: Completed Suicide |
| rs62535711 | 9 | 37174829 | T | 0.05 | 0.06 | 0.06 | 0.06 | 0.06 | 0.06 | 0.05 |
| rs598046 | 11 | 99516468 | T | 0.32 | 0.33 | 0.32 | 0.32 | 0.32 | 0.31 | 0.34 |
| rs7989250 | 13 | 64900801 | A | 0.33 | 0.32 | 0.31 | 0.31 | 0.32 | 0.31 | 0.29 |
