# Supplementary material for: Identification of novel genome-wide associations for suicidality in UK Biobank, genetic correlation with psychiatric disorders and polygenic association with completed suicide
Source: eBioMedicine. 2019 Feb 8;41:517–25. doi: 10.1016/j.ebiom.2019.02.005 (PMC6442001; doi:10.1016/j.ebiom.2019.02.005)
Supplement: Supplementary Table 4 — Effect of genetic loading for suicidal behaviour on psychiatric disorders and related traits. [file mmc14.docx]

| **Supplemental Table 4: Effect of genetic loading for suicidal behaviour on psychiatric traits** | | | | | | | | | | |  |  |  |
| --- | --- | --- | --- | --- | --- | --- | --- | --- | --- | --- | --- | --- | --- |
| Trait | GWAS threshold |  | *Unadjusted* | |  |  |  |  |  | *Adjusted* | |  |  |
|  |  | N | OR | 95% CI | | P | FDR adj P | N | OR | 95% CI | | P | FDR adj P |
| Self-report BD | 5.00E-08 | 72714 | 1.03 | 0.96 | 1.10 | 0.43 | 0.43 | 72610 | 1.03 | 0.96 | 1.10 | 0.41 | 0.41 |
|  | 5.00E-05 | 72714 | 1.11 | 1.03 | 1.18 | **0.0041** | **0.0049** | 72610 | 1.10 | 1.03 | 1.18 | **0.0049** | **0.0059** |
|  | 0.01 | 72714 | 1.16 | 1.08 | 1.25 | **3.90E-05** | **5.85E-05** | 72610 | 1.15 | 1.07 | 1.24 | **1.10E-04** | **1.65E-04** |
|  | 0.05 | 72714 | 1.17 | 1.09 | 1.26 | **2.10E-05** | **4.20E-05** | 72610 | 1.16 | 1.08 | 1.25 | **9.10E-05** | **1.65E-04** |
|  | 0.1 | 72714 | 1.19 | 1.10 | 1.28 | **6.50E-06** | **1.95E-05** | 72610 | 1.17 | 1.09 | 1.26 | **3.20E-05** | **9.60E-05** |
|  | 0.5 | 72714 | 1.22 | 1.12 | 1.32 | **1.40E-06** | **8.40E-06** | 72610 | 2.20 | 1.11 | 1.30 | **8.70E-06** | **5.22E-05** |
| Self-report Depression | 5.00E-08 | 82338 | 1.01 | 0.99 | 1.03 | 0.33 | 0.33 | 82217 | 1.01 | 0.99 | 1.03 | 0.38 | 0.380 |
|  | 5.00E-05 | 82338 | 1.03 | 1.01 | 1.05 | **0.009** | **0.0108** | 82217 | 1.03 | 1.01 | 1.04 | **0.012** | **0.0144** |
|  | 0.01 | 82338 | 1.09 | 1.07 | 1.11 | **8.80E-18** | **1.32E-17** | 82217 | 1.09 | 1.07 | 1.11 | **5.40E-17** | **8.10E-17** |
|  | 0.05 | 82338 | 1.11 | 1.09 | 1.14 | **1.90E-26** | **3.80E-26** | 82217 | 1.11 | 1.09 | 1.14 | **2.50E-25** | **5.00E-25** |
|  | 0.1 | 82338 | 1.12 | 1.09 | 1.14 | **4.30E-27** | **1.29E-26** | 82217 | 1.12 | 1.09 | 1.14 | **7.20E-26** | **2.16E-25** |
|  | 0.5 | 82338 | 1.13 | 1.10 | 1.15 | **3.80E-27** | **1.29E-26** | 82217 | 1.12 | 1.10 | 1.15 | **4.40E-26** | **2.16E-25** |
| Mood Instability | 5.00E-08 | 283088 | 1.01 | 1.00 | 1.02 | **0.0037** | **0.0037** | 282761 | 1.01 | 1.00 | 1.02 | **0.003** | **0.003** |
|  | 5.00E-05 | 283088 | 1.02 | 1.02 | 1.03 | **2.70E-09** | **3.24E-09** | 282761 | 1.02 | 1.02 | 1.03 | **1.70E-09** | **2.04E-09** |
|  | 0.01 | 283088 | 1.07 | 1.06 | 1.07 | **2.40E-61** | **3.60E-61** | 282761 | 1.06 | 1.06 | 1.07 | **1.90E-54** | **2.85E-54** |
|  | 0.05 | 283088 | 1.08 | 1.07 | 1.09 | **6.20E-86** | **1.24E-85** | 282761 | 1.08 | 1.07 | 1.09 | **1.60E-75** | **3.20E-75** |
|  | 0.1 | 283088 | 1.09 | 1.08 | 1.09 | **2.20E-94** | **6.60E-94** | 282761 | 1.08 | 1.07 | 1.09 | **6.20E-83** | **1.86E-82** |
|  | 0.5 | 283088 | 1.10 | 1.09 | 1.11 | **6.00E-108** | **3.60E-107** | 282761 | 1.09 | 1.09 | 1.10 | **1.40E-95** | **8.40E-95** |
| Risk taking | 5.00E-08 | 280508 | 1.00 | 0.99 | 1.01 | **0.6200** | **0.62** | 280183 | 1.00 | 0.99 | 1.01 | **0.75** | **0.75** |
|  | 5.00E-05 | 280508 | 1.01 | 1.01 | 1.02 | **0.0021** | **0.0025** | 280183 | 1.02 | 1.01 | 1.02 | **6.60E-04** | **7.92E-04** |
|  | 0.01 | 280508 | 1.03 | 1.03 | 1.04 | **1.10E-13** | **1.65E-13** | 280183 | 1.03 | 1.02 | 1.04 | **1.70E-12** | **2.55E-12** |
|  | 0.05 | 280508 | 1.04 | 1.03 | 1.05 | **2.60E-16** | **5.20E-16** | 280183 | 1.04 | 1.03 | 1.05 | **3.70E-14** | **7.40E-14** |
|  | 0.1 | 280508 | 1.04 | 1.03 | 1.05 | **5.80E-18** | **1.80E-17** | 280183 | 1.10 | 1.03 | 1.05 | **1.30E-15** | **7.80E-15** |
|  | 0.5 | 280508 | 1.04 | 1.03 | 1.05 | **6.00E-18** | **1.80E-17** | 280183 | 1.04 | 1.03 | 1.05 | **4.80E-15** | **1.44E-14** |
|  |  | N | Beta | SE |  | P | FDR adj P | N | Beta | SE |  | P | FDR adj P |
| Neuroticism (score) | 5.00E-08 | 232471 | 0.03 | 0.01 |  | **2.20E-04** | **2.20E-04** | 232205 | 0.03 | 0.01 |  | **2.20E-04** | **2.20E-04** |
|  | 5.00E-05 | 232471 | 0.05 | 0.01 |  | **3.20E-13** | **3.84E-13** | 232205 | 0.05 | 0.01 |  | **1.70E-13** | **2.04E-13** |
|  | 0.01 | 232471 | 0.15 | 0.01 |  | **3.20E-95** | **4.80E-95** | 232205 | 0.14 | 0.01 |  | **5.70E-89** | **8.55E-89** |
|  | 0.05 | 232471 | 0.18 | 0.01 |  | **2.00E-142** | **4.00E-142** | 232205 | 0.17 | 0.01 |  | **9.00E-133** | **1.80E-132** |
|  | 0.1 | 232471 | 0.19 | 0.01 |  | **4.00E-158** | **1.20E-157** | 232205 | 0.18 | 0.01 |  | **8.00E-148** | **2.40E-147** |
|  | 0.5 | 232471 | 0.22 | 0.01 |  | **6.00E-181** | **3.60E-180** | 232205 | 0.21 | 0.01 |  | **9.00E-171** | **5.40E-170** |
| Where: GWAS threshold, GWAS p value threshold for SNP inclusion in the polygenic score | | | | | | | | | | |  |  |  |
