# Supplementary material for: Identification of novel genome-wide associations for suicidality in UK Biobank, genetic correlation with psychiatric disorders and polygenic association with completed suicide
Source: eBioMedicine. 2019 Feb 8;41:517–25. doi: 10.1016/j.ebiom.2019.02.005 (PMC6442001; doi:10.1016/j.ebiom.2019.02.005)
Supplement: Supplementary Table 5 — Effect of genetic loading for suicidal behaviour on traits of relevance to psychiatric disorders [file mmc15.docx]

| **Supplemental Table 5: Functions of genes in novel suicidality loci** | | |
| --- | --- | --- |
| Gene | Name | Genecards summary |
| *ADCK3/COQ8A* | coenzyme Q8A | This gene encodes a mitochondrial protein similar to yeast ABC1, which functions in an electron-transferring membrane protein complex in the respiratory chain. It is not related to the family of ABC transporter proteins. Expression of this gene is induced by the tumor suppressor p53 and in response to DNA damage, and inhibiting its expression partially suppresses p53-induced apoptosis. Alternatively spliced transcript variants have been found; however, their full-length nature has not been determined. |
| *AP001877.1* |  | predicted gene |
| *CDKAL1* | CDK5 regulatory subunit associated protein 1 like 1 | The protein encoded by this gene is a member of the methylthiotransferase family. The function of this gene is not known. Genome-wide association studies have linked single nucleotide polymorphisms in an intron of this gene with susceptibilty to type 2 diabetes |
| *CEP57* | centrosomal protein 57 | This gene encodes a cytoplasmic protein called Translokin. This protein localizes to the centrosome and has a function in microtubular stabilization. The N-terminal half of this protein is required for its centrosome localization and for its multimerization, and the C-terminal half is required for nucleating, bundling and anchoring microtubules to the centrosomes. This protein specifically interacts with fibroblast growth factor 2 (FGF2), sorting nexin 6, Ran-binding protein M and the kinesins KIF3A and KIF3B, and thus mediates the nuclear translocation and mitogenic activity of the FGF2. It also interacts with cyclin D1 and controls nucleocytoplasmic distribution of the cyclin D1 in quiescent cells. This protein is crucial for maintaining correct chromosomal number during cell division. Mutations in this gene cause mosaic variegated aneuploidy syndrome, a rare autosomal recessive disorder. Multiple alternatively spliced transcript variants encoding different isoforms have been identified. |
| *CLTA* | Clathrin Light Chain A | Clathrin is a large, soluble protein composed of heavy and light chains. It functions as the main structural component of the lattice-type cytoplasmic face of coated pits and vesicles which entrap specific macromolecules during receptor-mediated endocytosis. This gene encodes one of two clathrin light chain proteins which are believed to function as regulatory elements. Alternative splicing results in multiple transcript variants. Related pseudogenes have been identified on chromosomes 8 and 12. Diseases associated with CLTA include Leber Congenital Amaurosis. Among its related pathways are Clathrin derived vesicle budding and EPH-Ephrin signaling. Gene Ontology (GO) annotations related to this gene include structural molecule activity and clathrin heavy chain binding. An important paralog of this gene is CLTB. Acts as component of the TACC3/ch-TOG/clathrin complex proposed to contribute to stabilization of kinetochore fibers of the mitotic spindle by acting as inter-microtubule bridge. |
| *CNTN5* | Contactin 5 | The protein encoded by this gene is a member of the immunoglobulin superfamily, and contactin family, which mediate cell surface interactions during nervous system development. This protein is a glycosylphosphatidylinositol (GPI)-anchored neuronal membrane protein that functions as a cell adhesion molecule. It may play a role in the formation of axon connections in the developing nervous system. Alternatively spliced transcript variants encoding different isoforms have been described for this gene. [provided by RefSeq, Aug 2011] CNTN5 (Contactin 5) is a Protein Coding gene. Diseases associated with CNTN5 include Actinomycosis. Among its related pathways are Metabolism of proteins and Post-translational modification- synthesis of GPI-anchored proteins. An important paralog of this gene is CNTN3. Contactins mediate cell surface interactions during nervous system development. Has some neurite outgrowth-promoting activity in the cerebral cortical neurons but not in hippocampal neurons. Probably involved in neuronal activity in the auditory system (By similarity). |
| *DCAF10* | DDB1 And CUL4 Associated Factor 10 | A protein coding gene. May function as a substrate receptor for CUL4-DDB1 E3 ubiquitin-protein ligase complex. |
| *DCC* | DCC netrin 1 receptor | This gene encodes a netrin 1 receptor. The transmembrane protein is a member of the immunoglobulin superfamily of cell adhesion molecules, and mediates axon guidance of neuronal growth cones towards sources of netrin 1 ligand. The cytoplasmic tail interacts with the tyrosine kinases Src and focal adhesion kinase (FAK, also known as PTK2) to mediate axon attraction. The protein partially localizes to lipid rafts, and induces apoptosis in the absence of ligand. The protein functions as a tumor suppressor, and is frequently mutated or downregulated in colorectal cancer and esophageal carcinoma. |
| *EBLN3* | endogenous Bornavirus-like nucleoprotein 3, pseudogene | psuedogene |
| *EIF4A1* | Eukaryotic tranlsation initiation factor 4A1 | Ubiquitous expression in many tissues |
| *EXOSC3* | Exosome Component 3 | This gene encodes a non-catalytic component of the human exosome, a complex with 3'-5' exoribonuclease activity that plays a role in numerous RNA processing and degradation activities. Related pseudogenes of this gene are found on chromosome 19 and 21. Alternatively spliced transcript variants encoding different isoforms have been described. Diseases associated with EXOSC3 include Pontocerebellar Hypoplasia, Type 1B and Exosc3-Related Pontocerebellar Hypoplasia. Among its related pathways are CDK-mediated phosphorylation and removal of Cdc6 and Gene Expression. Gene Ontology (GO) annotations related to this gene include RNA binding and exoribonuclease activity. Non-catalytic component of the RNA exosome complex which has 3->5 exoribonuclease activity and participates in a multitude of cellular RNA processing and degradation events. In the nucleus, the RNA exosome complex is involved in proper maturation of stable RNA species such as rRNA, snRNA and snoRNA, in the elimination of RNA processing by-products and non-coding pervasive transcripts, such as antisense RNA species and promoter-upstream transcripts (PROMPTs), and of mRNAs with processing defects, thereby limiting or excluding their export to the cytoplasm. The RNA exosome may be involved in Ig class switch recombination (CSR) and/or Ig variable region somatic hypermutation (SHM) by targeting AICDA deamination activity to transcribed dsDNA substrates. In the cytoplasm, the RNA exosome complex is involved in general mRNA turnover and specifically degrades inherently unstable mRNAs containing AU-rich elements (AREs) within their 3 untranslated regions, and in RNA surveillance pathways, preventing translation of aberrant mRNAs. It seems to be involved in degradation of histone mRNA. The catalytic inactive RNA exosome core complex of 9 subunits (Exo-9) is proposed to play a pivotal role in the binding and presentation of RNA for ribonucleolysis, and to serve as a scaffold for the association with catalytic subunits and accessory proteins or complexes. EXOSC3 as peripheral part of the Exo-9 complex stabilizes the hexameric ring of RNase PH-domain subunits through contacts with EXOSC9 and EXOSC5. |
| *FAM76B* | family with sequence similarity 76 member B |  |
| *FBXO10* | f-Box protein 10 | Members of the F-box protein family, such as FBXO10, are characterized by an approximately 40-amino acid F-box motif. SCF complexes, formed by SKP1 (MIM 601434), cullin (see CUL1; MIM 603134), and F-box proteins, act as protein-ubiquitin ligases. F-box proteins interact with SKP1 through the F box, and they interact with ubiquitination targets through other protein interaction domains (Jin et al., 2004 [PubMed 15520277]).[supplied by OMIM, Mar 2008] FBXO10 (F-Box Protein 10) is a Protein Coding gene. Among its related pathways are Innate Immune System and Class I MHC mediated antigen processing and presentation. Gene Ontology (GO) annotations related to this gene include ubiquitin-protein transferase activity. An important paralog of this gene is FBXO11. Substrate-recognition component of the SCF (SKP1-CUL1-F-box protein)-type E3 ubiquitin ligase complex. The SCF(FBXO10) complex mediates ubiquitination and degradation of BCL2, an antiapoptotic protein, thereby playing a role in apoptosis by controlling the stability of BCL2. |
| *FRMPD1* | FERM And PDZ Domain Containing 1 | FRMPD1 is a Protein Coding gene. An important paralog of this gene is FRMPD4. Stabilizes membrane-bound GPSM1, and thereby promotes its interaction with GNAI1. |
| *GNE* | glucosamine (UDP-N-acetyl)-2-epimerase/N-acetylmannosamine kinase | the protein encoded by this gene is a bifunctional enzyme that initiates and regulates the biosynthesis of N-acetylneuraminic acid (NeuAc), a precursor of sialic acids. It is a rate-limiting enzyme in the sialic acid biosynthetic pathway. Sialic acid modification of cell surface molecules is crucial for their function in many biologic processes, including cell adhesion and signal transduction. Differential sialylation of cell surface molecules is also implicated in the tumorigenicity and metastatic behavior of malignant cells. Mutations in this gene are associated with sialuria, autosomal recessive inclusion body myopathy, and Nonaka myopathy. Alternative splicing of this gene results in transcript variants encoding different isoforms. Diseases associated with GNE include Nonaka Myopathy and Sialuria. Among its related pathways are Metabolism of proteins and Transport to the Golgi and subsequent modification. Gene Ontology (GO) annotations related to this gene include hydrolase activity, hydrolyzing O-glycosyl compounds and UDP-N-acetylglucosamine 2-epimerase activity. Required for normal sialylation in hematopoietic cells. |
| *GRHPR* | Glyoxylate And Hydroxypyruvate Reductase | This gene encodes an enzyme with hydroxypyruvate reductase, glyoxylate reductase, and D-glycerate dehydrogenase enzymatic activities. The enzyme has widespread tissue expression and has a role in metabolism. Type II hyperoxaluria is caused by mutations in this gene. Diseases associated with GRHPR include Hyperoxaluria, Primary, Type Ii and Primary Hyperoxaluria. Among its related pathways are Glyoxylate metabolism and glycine degradation and Viral mRNA Translation. Gene Ontology (GO) annotations related to this gene include protein homodimerization activity and oxidoreductase activity, acting on the CH-OH group of donors, NAD or NADP as acceptor. Enzyme with hydroxy-pyruvate reductase, glyoxylate reductase and D-glycerate dehydrogenase enzymatic activities. Reduces hydroxypyruvate to D-glycerate, glyoxylate to glycolate oxidizes D-glycerate to hydroxypyruvate. |
| *LINC00395* | Long Intergenic Non-Protein Coding RNA 395 | long non-coding RNA |
| *MELK* | maternal embryonic leucine zipper kinase | A protein coding gene. Diseases associated with MELK include Uterine Corpus Endometrial Carcinoma. Among its related pathways are Neuroscience. Gene Ontology (GO) annotations related to this gene include *calcium ion binding* and *protein kinase activity*. An important paralog of this gene is PRKAA2. Serine/threonine-protein kinase involved in various processes such as cell cycle regulation, self-renewal of stem cells, apoptosis and splicing regulation. Has a broad substrate specificity; phosphorylates BCL2L14, CDC25B, MAP3K5/ASK1 and ZNF622. Acts as an activator of apoptosis by phosphorylating and activating MAP3K5/ASK1. Acts as a regulator of cell cycle, notably by mediating phosphorylation of CDC25B, promoting localization of CDC25B to the centrosome and the spindle poles during mitosis. Plays a key role in cell proliferation and carcinogenesis. Required for proliferation of embryonic and postnatal multipotent neural progenitors. Phosphorylates and inhibits BCL2L14, possibly leading to affect mammary carcinogenesis by mediating inhibition of the pro-apoptotic function of BCL2L14. Also involved in the inhibition of spliceosome assembly during mitosis by phosphorylating ZNF622, thereby contributing to its redirection to the nucleus. May also play a role in primitive hematopoiesis. |
| *MTMR2* | myotubularin related protein 2 | This gene is a member of the myotubularin family of phosphoinositide lipid phosphatases. The encoded protein possesses phosphatase activity towards phosphatidylinositol-3-phosphate and phosphatidylinositol-3,5-bisphosphate. Mutations in this gene are a cause of Charcot-Marie-Tooth disease type 4B, an autosomal recessive demyelinating neuropathy. Alternatively spliced transcript variants encoding multiple isoforms have been found for this gene |
| *OR7E156p* | Olfactory Receptor Family 7 Subfamily E Member 156 Pseudogene | psuedogene. Olfactory receptors interact with odorant molecules in the nose, to initiate a neuronal response that triggers the perception of a smell. The olfactory receptor proteins are members of a large family of G-protein-coupled receptors (GPCR) arising from single coding-exon genes. Olfactory receptors share a 7-transmembrane domain structure with many neurotransmitter and hormone receptors and are responsible for the recognition and G protein-mediated transduction of odorant signals. The olfactory receptor gene family is the largest in the genome. The nomenclature assigned to the olfactory receptor genes and proteins for this organism is independent of other organisms. |
| *PAX5* | paired box 5 | A transcription factors, whose central feature is a highly conserved DNA-binding motif (the paired box). Paired box transcription factors are important regulators in early development, and alterations in the expression of their genes are thought to contribute to neoplastic transformation. This gene encodes the B-cell lineage specific activator protein that is expressed at early, but not late stages of B-cell differentiation. Its expression has also been detected in developing CNS and testis and so the encoded protein may also play a role in neural development and spermatogenesis. This gene is located at 9p13, which is involved in t(9;14)(p13;q32) translocations recurring in small lymphocytic lymphomas of the plasmacytoid subtype, and in derived large-cell lymphomas. This translocation brings the potent E-mu enhancer of the IgH gene into close proximity of the PAX5 promoter, suggesting that the deregulation of transcription of this gene contributes to the pathogenesis of these lymphomas. Alternative splicing results in multiple transcript variants encoding different isoforms. T |
| *POLR1E* | RNA Polymerase I Subunit E | POLR1E (RNA Polymerase I Subunit E) is a Protein Coding gene. Among its related pathways are Pyrimidine metabolism (KEGG) and Gene Expression. Gene Ontology (GO) annotations related to this gene include DNA-directed 5-3 RNA polymerase activity and RNA polymerase I transcription factor binding. DNA-dependent RNA polymerase catalyzes the transcription of DNA into RNA using the four ribonucleoside triphosphates as substrates. Component of RNA polymerase I which synthesizes ribosomal RNA precursors. Appears to be involved in the formation of the initiation complex at the promoter by mediating the interaction between Pol I and UBTF/UBF (By similarity). |
| *RNF38* | ring finger protein 38 | This gene encodes a protein with a coiled-coil motif and a RING-H2 motif (C3H2C2) at its carboxy-terminus. The RING motif is a zinc-binding domain found in a large set of proteins playing roles in diverse cellular processes including oncogenesis, development, signal transduction, and apoptosis. Multiple transcript variants encoding different isoforms have been found for this gene. Acts as an E3 ubiquitin-protein ligase able to ubiquitinate p53/TP53 which promotes its relocalization to discrete foci associated with PML nuclear bodies. Exhibits preference for UBE2D2 as a E2 enzyme. Multiple transcript variants encoding different isoforms have been found for this gene. [provided by RefSeq, Dec 2008] |
| *SENP3* | SUMO specific peptidase 3 | The reversible posttranslational modification of proteins by the addition of small ubiquitin-like SUMO proteins (see SUMO1; MIM 601912) is required for numerous biologic processes. SUMO-specific proteases, such as SENP3, are responsible for the initial processing of SUMO precursors to generate a C-terminal diglycine motif required for the conjugation reaction. They also have isopeptidase activity for the removal of SUMO from high molecular mass SUMO conjugates |
| *SHB* | SH2 Domain Containing Adaptor Protein B | A protein coding gene. Diseases associated with SHB include Sulfhemoglobinemia. Among its related pathways are EPH-Ephrin signaling and Developmental Biology. Gene Ontology (GO) annotations related to this gene include SH3/SH2 adaptor activity. An important paralog of this gene is ENSG00000255872. Adapter protein which regulates several signal transduction cascades by linking activated receptors to downstream signaling components. May play a role in angiogenesis by regulating FGFR1, VEGFR2 and PDGFR signaling. May also play a role in T-cell antigen receptor/TCR signaling, interleukin-2 signaling, apoptosis and neuronal cells differentiation by mediating basic-FGF and NGF-induced signaling cascades. May also regulate IRS1 and IRS2 signaling in insulin-producing cells. |
| *SLC25A51* | Solute Carrier Family 25 Member 51 | A protein coding gene. An important paralog of this gene is SLC25A52. |
| *TOMM5* | Translocase Of Outer Mitochondrial Membrane 5 | TOMM5 (Translocase Of Outer Mitochondrial Membrane 5) is a Protein Coding gene. Among its related pathways are Pink/Parkin Mediated Mitophagy and Metabolism of proteins. Gene Ontology (GO) annotations related to this gene include *protein transporter activity*. An important paralog of this gene is ENSG00000256966. |
| *TRMT10B* | TRNA Methyltransferase 10B | TRMT10B is a Protein Coding gene. Gene Ontology (GO) annotations related to this gene include methyltransferase activity. An important paralog of this gene is TRMT10A. S-adenosyl-L-methionine-dependent guanine N(1)-methyltransferase that catalyzes the formation of N(1)-methylguanine at position 9 (m1G9) in tRNAs (PubMed:23042678). Probably not able to catalyze formation of N(1)-methyladenine at position 9 (m1A9) in tRNAs (PubMed:23042678). |
| *ZBTB5* | Zinc Finger And BTB Domain Containing 5 | ZBTB5 (Zinc Finger And BTB Domain Containing 5) is a Protein Coding gene. An important paralog of this gene is ZBTB3. May be involved in transcriptional regulation |
| *ZCCHC7* | Zinc Finger, CCHC Domain Containing 7 | A protein coding gene. Related pathways include Deadenylation-dependent mRNA decay. Gene Ontology (GO) annotations related to this gene include nucleic acid binding. |
