# Supplementary material for: Identification of novel genome-wide associations for suicidality in UK Biobank, genetic correlation with psychiatric disorders and polygenic association with completed suicide
Source: eBioMedicine. 2019 Feb 8;41:517–25. doi: 10.1016/j.ebiom.2019.02.005 (PMC6442001; doi:10.1016/j.ebiom.2019.02.005)
Supplement: Supplementary Table 6 — Functions of genes in novel suicidality loci eQTLs in brain of predicted functional SNPs in the CEP57-FAM76B locus [file mmc16.docx]

| **Supplemental Table 6: eQTLs in brain of predicted functional SNPs in the *CEP57-FAM76B* locus** | | | | |
| --- | --- | --- | --- | --- |
| SNP | bp | Gene Symbol | NES | Tissue |
| rs1150360 | 95512060 | *AP001877.1* | 0.45 | Brain - Caudate (basal ganglia) |
|  |  | *AP001877.1* | 0.6 | Brain - Cerebellar Hemisphere |
|  |  | *AP001877.1* | 0.56 | Brain - Cerebellum |
|  |  | *AP001877.1* | 0.59 | Brain - Frontal Cortex (BA9) |
|  |  | *AP001877.1* | 0.5 | Brain - Hippocampus |
|  |  | *AP001877.1* | 0.53 | Brain - Nucleus accumbens (basal ganglia) |
|  |  | *AP001877.1* | 0.48 | Brain - Putamen (basal ganglia) |
|  |  | *AP001877.1* | 0.58 | Brain - Spinal cord (cervical c-1) |
|  |  | *CEP57* | 0.21 | Brain - Cerebellum |
| rs3824874 | 95657111 | *AP001877.1* | 0.7 | Brain - Anterior cingulate cortex (BA24) |
|  |  | *AP001877.1* | 0.62 | Brain - Caudate (basal ganglia) |
|  |  | *AP001877.1* | 0.72 | Brain - Cerebellar Hemisphere |
|  |  | *AP001877.1* | 0.8 | Brain - Cerebellum |
|  |  | *AP001877.1* | 0.67 | Brain - Cortex |
|  |  | *AP001877.1* | 0.64 | Brain - Frontal Cortex (BA9) |
|  |  | *AP001877.1* | 0.64 | Brain - Hippocampus |
|  |  | *AP001877.1* | 0.69 | Brain - Hypothalamus |
|  |  | *AP001877.1* | 0.69 | Brain - Nucleus accumbens (basal ganglia) |
|  |  | *AP001877.1* | 0.47 | Brain - Putamen (basal ganglia) |
|  |  | *AP001877.1* | 0.79 | Brain - Spinal cord (cervical c-1) |
| rs644799 | 95564259 | *AP001877.1* | 0.71 | Brain - Anterior cingulate cortex (BA24) |
|  |  | *AP001877.1* | 0.63 | Brain - Caudate (basal ganglia) |
|  |  | *AP001877.1* | 0.77 | Brain - Cerebellar Hemisphere |
|  |  | *AP001877.1* | 0.83 | Brain - Cerebellum |
|  |  | *AP001877.1* | 0.7 | Brain - Cortex |
|  |  | *AP001877.1* | 0.73 | Brain - Frontal Cortex (BA9) |
|  |  | *AP001877.1* | 0.64 | Brain - Hippocampus |
|  |  | *AP001877.1* | 0.68 | Brain - Hypothalamus |
|  |  | *AP001877.1* | 0.68 | Brain - Nucleus accumbens (basal ganglia) |
|  |  | *AP001877.1* | 0.55 | Brain - Putamen (basal ganglia) |
|  |  | *AP001877.1* | 0.88 | Brain - Spinal cord (cervical c-1) |
|  |  | *AP001877.1* | 0.58 | Brain - Substantia nigra |
