# Supplementary material for: Identification of novel genome-wide associations for suicidality in UK Biobank, genetic correlation with psychiatric disorders and polygenic association with completed suicide
Source: eBioMedicine. 2019 Feb 8;41:517–25. doi: 10.1016/j.ebiom.2019.02.005 (PMC6442001; doi:10.1016/j.ebiom.2019.02.005)
Supplement: Supplementary Table 7 — Novel Suicidal behaviour loci and previous associations in the GWAS catalogue [file mmc17.docx]

| **Supplementary Table 7: Previous GWAS catalogue associations of novel suicidal behaviour loci** | | | | |  |  |  |
| --- | --- | --- | --- | --- | --- | --- | --- |
| Search Term | PUBMEDID | FIRST AUTHOR | DISEASE/TRAIT | STRONGEST SNP-RISK ALLELE | RAF | P-VALUE | OR or BETA |
| chr9 locus | 22449649 | Loo SK | **Intelligence** | rs1329573-?; rs7020413-?; rs3824344-?; rs3758171-? | NR | 4.00E-08 |  |
|  | 22491018 | Wang J | Response to tocilizumab in rheumatoid arthritis | rs1329568-? | 0.03 | 8.00E-07 | 19.640 |
|  | 26198764 | Goes FS | **Schizophrenia** | rs7020830-T | NR | 8.00E-06 | 1.053 |
|  | 25786224 | Johnson EO | HIV-1 susceptibility | rs4878712-A; rs1329568-T |  | 7.00E-07 | 1.640 |
|  | 25786224 | Johnson EO | HIV-1 susceptibility | rs4878712-A; rs1329568-T |  | 5.00E-08 |  |
| *TOMM5* | 28753643 | Yeo A | Lipoprotein phospholipase A2 activity in cardiovascular disease | rs57578064-A | 0.01 | 1.00E-08 | 32.490 |
| *DCAF10* | 27629369 | Gao J | **Loneliness (linear analysis)** | rs78173384-? |  | 2.00E-06 | 0.374 |
|  | 28928442 | Tian C | Cold sores | rs776014-? | NR | 7.00E-06 | 0.163 |
| *FRMPD1* | 29299148 | Liu W | Cancer | rs2182318-?; rs7856656-? | NR | 1.00E-06 | 1.390 |
|  | 25786224 | Johnson EO | HIV-1 susceptibility | rs4878712-? |  | 4.00E-07 |  |
|  | 25786224 | Johnson EO | HIV-1 susceptibility | rs4878712-A | 0.69 | 5.00E-07 | 1.320 |
|  | 25786224 | Johnson EO | HIV-1 susceptibility | rs4878712-A; rs1329568-T |  | 7.00E-07 | 1.640 |
|  | 25786224 | Johnson EO | HIV-1 susceptibility | rs4878712-A; rs1329568-T |  | 5.00E-08 |  |
| *SHB* | 23934736 | Yu B | Metabolite levels (Dihydroxy docosatrienoic acid) | rs3747547-C |  | 5.00E-06 | 0.090 |
|  | 27629369 | Gao J | **Loneliness (linear analysis)** | rs149411702-? |  | 2.00E-06 | 0.376 |
|  | 20171287 | Stein JL | **Brain structure** | rs7873102-? | 0.38 | 6.00E-07 |  |
|  | 28060188 | Hamet P | Type 2 diabetes (age of onset) | rs10973627-? | NR | 4.00E-06 |  |
| *PAX5* | 22449649 | Loo SK | Intelligence | rs1329573-?; rs7020413-?; rs3824344-?; rs3758171-? | NR | 4.00E-08 |  |
|  | 22013104 | Melka MG | Obesity-related traits | rs16933812-? |  | 5.00E-06 |  |
|  | 22013104 | Melka MG | Obesity-related traits | rs16933812-? |  | 9.00E-09 |  |
|  | 25786224 | Johnson EO | HIV-1 susceptibility | rs4878712-? |  | 4.00E-07 |  |
|  | 25786224 | Johnson EO | HIV-1 susceptibility | rs4878712-A | 0.69 | 5.00E-07 | 1.320 |
|  | 22491018 | Wang J | Response to tocilizumab in rheumatoid arthritis | rs1329568-? | 0.03 | 8.00E-07 | 19.640 |
|  | 25786224 | Johnson EO | HIV-1 susceptibility | rs4878712-A; rs1329568-T |  | 7.00E-07 | 1.640 |
|  | 25786224 | Johnson EO | HIV-1 susceptibility | rs4878712-A; rs1329568-T |  | 5.00E-08 |  |
| *ELBN3* | 26198764 | Goes FS | **Schizophrenia** | rs7020830-T | NR | 8.00E-06 | 1.053 |
| *ZCCHC7* | 25786224 | Johnson EO | HIV-1 susceptibility | rs4878712-? |  | 4.00E-07 |  |
|  | 25786224 | Johnson EO | HIV-1 susceptibility | rs4878712-A | 0.69 | 5.00E-07 | 1.320 |
| *GRHPR* | 29299148 | Liu W | Cancer | rs2182318-?; rs7856656-? | NR | 1.00E-06 | 1.390 |
| *FBXO10* | 27989323 | Ahola-Olli AV | Macrophage inflammatory protein 1b levels | rs76582507-G | NR | 3.00E-06 | 0.318 |
|  | 25786224 | Johnson EO | HIV-1 susceptibility | rs4878712-? |  | 4.00E-07 |  |
|  | 25786224 | Johnson EO | HIV-1 susceptibility | rs4878712-A | 0.69 | 5.00E-07 | 1.320 |
| chr11 locus | 26503763 | Song J | **Objective response to lithium treatment in bipolar disorder** | rs113262272-A | 0.71 | 4.00E-06 | 1.930 |
|  | 26911590 | Mullin BH | Bone mineral density (femoral neck) | rs10893396-C | 0.17 | 4.00E-07 | 0.150 |
|  | 27629369 | Gao J | **Loneliness (multivariate analysis)** | rs10893420-? | NR | 7.00E-06 |  |
| *CNTN5* | 23049088 | Meng W | Myopia (pathological) | rs12803066-? | 0.52 | 4.00E-06 |  |
|  | 23251661 | Comuzzie AG | Obesity-related traits | rs11217223-G | 0.09 | 1.00E-06 | 0.040 |
|  | 23251661 | Comuzzie AG | Obesity-related traits | rs11217223-G | 0.09 | 6.00E-06 | 0.030 |
|  | 27629369 | Gao J | **Loneliness (multivariate analysis)** | rs10893420-? | NR | 7.00E-06 |  |
|  | 26365420 | Mbarek H | **Alcohol dependence** | rs117557854-A | 0.02 | 2.00E-06 | 2.083 |
|  | 17903304 | Larson MG | Atrial fibrillation | rs10501920-? | NR | 9.00E-06 |  |
|  | 17903297 | Seshadri S | **Volumetric brain MRI** | rs952700-? | NR | 6.00E-06 |  |
|  | 22610502 | Kennedy RB | Immune reponse to smallpox (secreted IL-2) | rs11223581-G |  | 9.00E-09 |  |
|  | 26911590 | Mullin BH | Bone mineral density (femoral neck) | rs10893396-C | 0.17 | 4.00E-07 | 0.150 |
|  | 23667675 | Tanikawa C | Menarche (age at onset) | rs12800752-T | 0.8 | 3.00E-06 | 0.095 |
|  | 28240269 | Suhre K | Blood protein levels | rs1461672-T | 0.06 | 2.00E-18 | 0.734 |
|  | 29317604 | Liu D | Plasma kynurenine levels in major depressive disorder | rs61475845-A | 0.02 | 1.00E-06 | 2.200 |
|  | 20889312 | Wang KS | **Bipolar disorder and schizophrenia** | rs2509843-? | NR | 2.00E-07 | 1.268 |
|  | 24529757 | Xie T | Amyotrophic lateral sclerosis (sporadic) | kgp11394149-? |  | 2.00E-07 |  |
|  | 27989323 | Ahola-Olli AV | Interleukin-2 receptor antagonist levels | rs73001149-C |  | 9.00E-06 | 0.549 |
|  | 29292387 | Turley P | **Neuroticism** | rs1690816-C | 0.31 | 3.00E-06 | 0.017 |
|  | 29292387 | Turley P | **Depressive symptoms (multi-trait analysis)** | rs586533-G | 0.32 | 2.00E-09 | 0.014 |
|  | 29292387 | Turley P | **Neuroticism (multi-trait analysis)** | rs1690816-C | 0.31 | 3.00E-09 | 0.018 |
|  | 26503763 | Song J | **Objective response to lithium treatment in bipolar disorder** | rs113262272-A | 0.71 | 4.00E-06 | 1.930 |
|  | 25646338 | Mozaffarian D | Trans fatty acid levels | rs7952067-C | 0.07 | 1.00E-06 | 0.005 |
|  | 20708005 | Chalasani N | Non-alcoholic fatty liver disease histology (lobular) | rs4237591-G | 0.35 | 2.00E-06 | 0.330 |
|  | 26634245 | Lutz SM | Post bronchodilator FEV1/FVC ratio in COPD | rs1942108-A | 0.49 | 5.00E-06 | 0.011 |
|  | 27863252 | Astle WJ | Lymphocyte counts | rs7939778-A | 0.26 | 2.00E-10 | 0.026 |
|  | 27863252 | Astle WJ | Reticulocyte fraction of red cells | rs72996113-T | 0.1 | 2.00E-17 | 0.051 |
|  | 27863252 | Astle WJ | Reticulocyte count | rs72996113-T | 0.1 | 1.00E-17 | 0.052 |
|  | 27863252 | Astle WJ | Plateletcrit | rs1111890-G | 0.37 | 2.00E-13 | 0.028 |
|  | 27863252 | Astle WJ | Red cell distribution width | rs717662-T | 0.11 | 4.00E-12 | 0.040 |
|  | 28898252 | Wheeler E | Glycated hemoglobin levels | rs11224302-C | 0.87 | 5.00E-07 | 0.015 |
|  | 25673412 | Shungin D | Waist-hip ratio | rs1394461-C | 0.25 | 4.00E-08 | 0.035 |
|  | 27089181 | Okbay A | **Neuroticism** | rs2458167-A | 0.3 | 2.00E-07 | 0.019 |
|  | 29273806 | Demenais F | Asthma | rs3758697-A | 0.3 | 2.00E-06 | 1.330 |
|  | 27089181 | Okbay A | **Depression** | rs1690818-T | NR | 6.00E-08 | 5.405 |
| chr13 locus | 25935106 | Kim KW | Recalcitrant atopic dermatitis | rs9540294-G | 0.08 | 1.00E-08 | 2.655 |
|  | 25760438 | Anderson D | Type 2 diabetes | rs11454281-? | NR | 9.00E-06 | 0.600 |
| *DCC* | 22318345 | Cha PC | Gallbladder cancer | rs7504990-A | 0.21 | 7.00E-08 | 6.950 |
|  | 23251661 | Comuzzie AG | Obesity-related traits | rs4940203-A | 0.29 | 5.00E-07 | 0.050 |
|  | 27149984 | Degenhardt F | Coenzyme Q10 levels | rs74681568-G |  | 2.00E-07 | 0.116 |
|  | 24009623 | Jiang J | Response to mTOR inhibitor (everolimus) | rs1460196-? | NR | 9.00E-06 | 0.270 |
|  | 23192594 | Velez Edwards DR | Body mass index (ever vs never smoking interaction) | rs11876941-A | NR | 5.00E-06 | 0.003 |
|  | 28199695 | Jones AV | Mosquito bite size | rs141670172-T | NR | 7.00E-06 | 0.257 |
|  | 29326435 | Hill WD | **Intelligence (multi-trait analysis)** | rs7245004-A | NR | 7.00E-14 | 0.022 |
|  | 29326435 | Hill WD | **Intelligence (multi-trait analysis)** | rs8083850-A | NR | 6.00E-13 | 0.021 |
|  | 29326435 | Hill WD | **Intelligence (multi-trait analysis)** | rs11663156-T | NR | 1.00E-08 | 0.019 |
|  | 29326435 | Hill WD | **Intelligence (multi-trait analysis)** | rs11665242-A | NR | 9.00E-16 | 0.024 |
|  | 29326435 | Hill WD | **Intelligence (multi-trait analysis)** | rs12607356-A | NR | 2.00E-09 | 0.018 |
|  | 29326435 | Hill WD | **Intelligence (multi-trait analysis)** | rs12960505-T | NR | 2.00E-15 | 0.024 |
|  | 29326435 | Hill WD | **Intelligence (multi-trait analysis)** | rs10502966-A | NR | 1.00E-11 | 0.020 |
|  | 29326435 | Hill WD | **Intelligence (multi-trait analysis)** | rs1972044-A | NR | 7.00E-14 | 0.023 |
|  | 29326435 | Hill WD | **Intelligence (multi-trait analysis)** | rs6508220-A | NR | 5.00E-15 | 0.023 |
|  | 29326435 | Hill WD | **Intelligence (multi-trait analysis)** | rs7506451-A | NR | 3.00E-10 | 0.019 |
|  | 29326435 | Hill WD | **Intelligence (multi-trait analysis)** | rs10221412-T | NR | 4.00E-11 | 0.020 |
|  | 29326435 | Hill WD | **Intelligence (multi-trait analysis)** | rs1078459-T | NR | 3.00E-11 | 0.020 |
|  | 29326435 | Hill WD | **Intelligence (multi-trait analysis)** | rs1367633-A | NR | 2.00E-08 | 0.017 |
|  | 29326435 | Hill WD | **Intelligence (multi-trait analysis)** | rs17417046-T | NR | 3.00E-13 | 0.021 |
|  | 29326435 | Hill WD | **Intelligence (multi-trait analysis)** | rs4277413-A | NR | 5.00E-13 | 0.021 |
|  | 29147026 | Chen CH | **Putamen volume** | rs11660938-? | NR | 4.00E-12 | 41.504 |
|  | 29187730 | Ward J | **Mood instability** | rs8084280-T | 0.51 | 3.00E-09 | 0.050 |
|  | 29187730 | Ward J | **Mood instability** | rs8084280-T | 0.51 | 1.00E-07 | 0.047 |
|  | 26025379 | Ng E | Nickel levels | chr18:51115162-? |  | 3.00E-06 | 1.510 |
|  | 22318345 | Cha PC | Gallbladder cancer | rs975334-C | 0.16 | 9.00E-07 | 8.300 |
|  | 22318345 | Cha PC | Gallbladder cancer | rs13294589-G | 0.11 | 2.00E-06 | 12.780 |
|  | 22318345 | Cha PC | Gallbladder cancer | rs6869388-C | 0.04 | 7.00E-06 | 72.700 |
|  | 22318345 | Cha PC | Gallbladder cancer | rs10953615-C | 0.11 | 9.00E-06 | 7.510 |
|  | 21529783 | Heath AC | **Alcoholism (alcohol use disorder factor score)** | rs768048-C | 0.12 | 8.00E-06 | 0.110 |
|  | 28317148 | Campo C | Bortezomib-induced peripheral neuropathy in multiple myeloma | rs17748074-A | NR | 9.00E-06 | 1.960 |
|  | 27393504 | Zanetti KA | **Smoking behaviour (cigarettes smoked per day)** | rs1372626-? | NR | 3.00E-06 |  |
|  | 28892062 | Akiyama M | Body mass index | rs4129322-A | 0.1 | 4.00E-08 | 0.018 |
|  | 25524916 | Palmer ND | Glucose homeostasis traits | rs2339345-? |  | 8.00E-06 | 0.250 |
|  | 29186694 | Lam M | **Cognitive ability** | rs1431196-? | NR | 4.00E-07 | 5.073 |
|  | 29186694 | Lam M | **Cognitive ability (multi-trait analysis)** | rs1431196-? | NR | 6.00E-11 | 6.546 |
|  | 26491034 | Sarzynski MA | Response to exercise (triglyceride level interaction) | rs3906453-A | 0.76 | 3.00E-06 | 0.390 |
|  | 27015805 | Pilling LC | Parental longevity (combined parental age at death) | rs67163261-? | NR | 1.00E-06 | 0.039 |
|  | 29292387 | Turley P | **Depressive symptoms** | rs11663393-G | 0.55 | 6.00E-09 | 0.014 |
|  | 29292387 | Turley P | **Depressive symptoms (multi-trait analysis)** | rs8084351-G | 0.49 | 2.00E-12 | 0.015 |
|  | 29292387 | Turley P | **Subjective well-being (multi-trait analysis)** | rs8084351-G | 0.48 | 4.00E-12 | 0.013 |
|  | 29292387 | Turley P | **Neuroticism (multi-trait analysis)** | rs8084351-G | 0.49 | 1.00E-13 | 0.021 |
|  | 25017104 | Kottyan LC | Eosinophilic esophagitis | rs9956738-? | 0.01 | 4.00E-07 | 2.472 |
|  | 27002377 | Irvin MR | Response to fenofibrate (total cholesterol levels) | chr18:50464126-? | NR | 2.00E-06 | 0.017 |
|  | 26198764 | Goes FS | **Schizophrenia** | rs4632195-T | NR | 4.00E-06 | 1.050 |
|  | 26586795 | Phipps AI | Survival in colorectal cancer (distant metastatic) | rs1372474-G | 0.1 | 2.00E-06 | 1.530 |
|  | 26586795 | Phipps AI | Survival in colorectal cancer (distant metastatic) | rs1442089-C | 0.09 | 2.00E-06 | 1.560 |
|  | 28991256 | Li Z | **Schizophrenia** | rs4632195-T | NR | 2.00E-06 | 1.051 |
|  | 27089181 | Okbay A | **Depression** | rs62100776-A | NR | 1.00E-08 | 5.664 |
|  | 25607358 | Hibar DP | **Subcortical brain region volumes** | rs62097986-A | 0.44 | 1.00E-13 | 30.280 |
|  | 27089181 | Okbay A | **Neuroticism** | rs4632195-T | 0.52 | 5.00E-07 | 0.018 |
|  | 27225129 | Okbay A | **Educational attainment (years of education)** | rs62100765-T | 0.42 | 1.00E-10 | 0.015 |
| *PSMD14* | 26989097 | Coleman JR | **Response to cognitive-behavioural therapy in anxiety disorder** | rs13432654-? | 0.09 | 8.00E-06 |  |
|  | 25644384 | Davies G | **Cognitive ability** | rs2303319-? | NR | 9.00E-06 | 0.045 |
|  | 25201988 | Rietveld CA | **Educational attainment** | rs7309-A | 0.49 | 2.00E-07 | 0.022 |
| *ACK3/ COQ8A* | 27863252 | Astle WJ | Sum neutrophil eosinophil counts | rs17592479-A | 0.51 | 7.00E-13 | 0.026 |
|  | 27863252 | Astle WJ | Sum basophil neutrophil counts | rs17592479-A | 0.51 | 4.00E-13 | 0.026 |
|  | 27863252 | Astle WJ | Granulocyte count | rs17592479-A | 0.51 | 1.00E-12 | 0.025 |
|  | 27863252 | Astle WJ | Granulocyte percentage of myeloid white cells | rs17592479-A | 0.51 | 4.00E-12 | 0.025 |
|  | 27863252 | Astle WJ | Lymphocyte percentage of white cells | rs2297412-G | 0.48 | 3.00E-11 | 0.024 |
|  | 27863252 | Astle WJ | Neutrophil count | rs17592479-A | 0.51 | 3.00E-13 | 0.026 |
|  | 27863252 | Astle WJ | Neutrophil percentage of white cells | rs6426558-T | 0.51 | 3.00E-14 | 0.027 |
| *CEP57* | 29326435 | Hill WD | **Intelligence (multi-trait analysis)** | rs644799-A | NR | 1.00E-09 | 0.018 |
|  | 29186694 | Lam M | **Cognitive ability (multi-trait analysis)** | rs644799-? | NR | 2.00E-09 | 6.011 |
|  | 24390342 | Okada Y | Rheumatoid arthritis | rs4409785-C | 0.15 | 1.00E-11 | 1.120 |
|  | 24390342 | Okada Y | Rheumatoid arthritis | rs4409785-C | 0.17 | 4.00E-09 | 1.120 |
| *FAM76B* | 29083406 | Ferreira MA | Allergic disease (asthma, hay fever or eczema) | rs59593577-C | 0.87 | 2.00E-11 | 1.053 |
| Where: RAF, risk-allele frequency; chr 9 locus, 9:36999369-37360767; chr11 locus, 11:99392678-99588751; chr13 locus, 13:64900801-65036538 | | | | | | | |
