# Supplementary material for: Identification of novel genome-wide associations for suicidality in UK Biobank, genetic correlation with psychiatric disorders and polygenic association with completed suicide
Source: eBioMedicine. 2019 Feb 8;41:517–25. doi: 10.1016/j.ebiom.2019.02.005 (PMC6442001; doi:10.1016/j.ebiom.2019.02.005)
Supplement: Supplementary Table 8 — Previously reported suicidal behaviour-associated SNPs [file mmc18.docx]

| **Supplementary Table 8: Previously reported suicidal behaviour-associated SNPs** | | | | | |  |  |  |  |  |  |  |  |  |
| --- | --- | --- | --- | --- | --- | --- | --- | --- | --- | --- | --- | --- | --- | --- |
| Previously reported associations | | | | | | | | | Association with suicidality in this GWAS | | | | | |
| PMID | FIRST AUTHOR | DISEASE/TRAIT | CHR | BP | STRONGEST SNP-RISK ALLELE | RAF | P-VALUE | OR or BETA | EA | OA | BETA | SE | P | RAF |
| 20877300 | Perroud N | Suicidal ideation | 4 | 21,475,367 | rs358592-? | 0.70 | 3.00E-06 | 2.56 |  |  |  |  |  |  |
| 20877300 | Perroud N |  | 8 | 28,065,871 | rs4732812-? | 0.73 | 3.00E-06 | 2.56 |  |  |  |  |  |  |
| 20877300 | Perroud N |  | 9 | 72,272,787 | rs11143230-C | 0.35 | 8.00E-07 | 1.88 | A | C | 0.02 | 0.01 | **0.0475** | 0.33 |
| 20877300 | Perroud N |  | 9 | 72,272,787 | rs11143230-C | 0.35 | 7.00E-06 | 2.22 |  |  |  |  |  |  |
| 21041247 | Perlis RH | Suicide risk in mood disorders | 2 | 46,093,955 | rs12373805-A | NR | 9.00E-06 | 1.22 | A | G | 0.00 | 0.01 | 0.7678 | 0.20 |
| 21041247 | Perlis RH |  | 10 | 30,201,775 | rs2462021-C | NR | 8.00E-06 | 1.18 | T | C | 0.01 | 0.01 | 0.1232 | 0.36 |
| 21041247 | Perlis RH |  | 10 | 95,362,484 | rs4918918-T | NR | 3.00E-06 | 1.18 |  |  |  |  |  |  |
| 21041247 | Perlis RH |  | 21 | 39,649,825 | rs10854398-C | NR | 6.00E-06 | 1.18 |  |  |  |  |  |  |
| 21423239 | Willour VL | Suicide attempts in bipolar disorder | 2 | 112,496 | rs300774-A | 0.18 | **5.00E-08** | 1.22 |  |  |  |  |  |  |
| 21423239 | Willour VL |  | 11 | 33,566,664 | rs10437629-? | NR | 4.00E-06 | 1.34 |  |  |  |  |  |  |
| 21423239 | Willour VL |  | 12 | 128,610,527 | rs7296262-T | 0.51 | 1.00E-06 | 1.22 | T | C | 0.00 | 0.01 | 0.9861 | 0.45 |
| 24964207 | Mullins N | Suicide attempts in depression or bipolar disorder | 4 | 15,993,502 | rs17387100-G | 0.08 | 8.00E-07 | 1.76 |  |  |  |  |  |  |
| 24964207 | Mullins N |  | 7 | 150,339,575 | rs17173608-G | 0.06 | 2.00E-07 | 1.93 |  |  |  |  |  |  |
| 24964207 | Mullins N |  | 11 | 113,249,490 | rs3781878-? | 0.73 | 2.00E-06 | 1.54 |  |  |  |  |  |  |
| 24964207 | Mullins N |  | 12 | 66,422,359 | rs10748045-G | 0.35 | 1.00E-06 | 1.78 |  |  |  |  |  |  |
| 25917933 | Zai CC | Suicide in bipolar disorder | 8 | 56,592,754 | rs2610025-A |  | 5.00E-06 | 0.02 | C | A | 0.00 | 0.01 | 0.6429 | 0.45 |
| 25917933 | Zai CC |  | 8 | 79,191,197 | rs10448044-C |  | 3.00E-06 | 4.69 |  |  |  |  |  |  |
| 25917933 | Zai CC |  | 10 | 32,704,340 | rs7079041-A |  | 2.00E-06 | 0.02 |  |  |  |  |  |  |
| 25917933 | Zai CC |  | 18 | 68,547,459 | rs7244261-T |  | 4.00E-06 | 4.61 |  |  |  |  |  |  |
| 26079190 | Galfalvy H | Suicide | 1 | 213,424,498 | rs320461-A | 0.23 | 4.00E-06 | 1.70 | C | T | 0.01 | 0.01 | 0.6049 | 0.22 |
| 26079190 | Galfalvy H |  | 7 | 35,254,361 | rs336284-A | 0.45 | 2.00E-07 | 1.66 | G | A | 0.00 | 0.01 | 0.6061 | 0.47 |
| 26079190 | Galfalvy H |  | 8 | 10,677,867 | rs7011192-A | 0.11 | 4.00E-06 | 2.45 | G | A | 0.02 | 0.02 | 0.3185 | 0.09 |
| 26079190 | Galfalvy H | Suicide attempts in major depressive disorder | 2 | 115,733,670 | rs4308128-A | 0.43 | 4.00E-06 | 1.80 | A | C | 0.01 | 0.01 | 0.2785 | 0.5 |
| 26079190 | Galfalvy H | Suicide behavior | 10 | 70,758,081 | rs6480463-G | 0.38 | 2.00E-06 | 1.42 | T | C | 0.01 | 0.01 | 0.4580 | 0.42 |
| 26079190 | Galfalvy H |  | 14 | 24,146,226 | rs4575-G | 0.27 | 8.00E-06 | 1.49 | T | C | 0.02 | 0.01 | **0.0099** | 0.28 |
| 26079190 | Galfalvy H |  | 15 | 34,813,456 | rs11852984-C | 0.20 | 2.00E-06 | 1.80 | A | C | 0.00 | 0.01 | 0.9746 | 0.18 |
| 26079190 | Galfalvy H |  | 8 | 98,883,177 | rs3019286-A | 0.36 | 8.00E-06 | 1.40 | G | A | 0.00 | 0.01 | 0.6161 | 0.41 |
| 26079190 | Galfalvy H | Suicide ideation score in major depressive disorder | 1 | 190,088,550 | rs2419374-A | 0.19 | 1.00E-06 | 3.46 | C | T | 0.02 | 0.01 | 0.1313 | 0.17 |
| 26079190 | Galfalvy H |  | 20 | 8,233,139 | rs6055685-A | 0.18 | 8.00E-07 | 3.57 | G | A | 0.01 | 0.01 | 0.5618 | 0.18 |
| 26079190 | Galfalvy H |  | 5 | 112,267,016 | rs13358904-G | 0.21 | 5.00E-06 | 3.05 | A | G | 0.01 | 0.01 | 0.2754 | 0.24 |
| 30116032 | Erlangsen A | Suicide attempts, with and without psychiatric diagnoses | 1 | 81,428,767 | rs72940689-A | 0.06 | 2.78E−07 | 0.79 | G | A | 0.05 | 0.02 | **0.0208** | 0.05 |
| 30116032 | Erlangsen A |  | 5 | 153,290,253 | rs2085865-A | 0.31 | 1.06E−07 | 0.89 | G | A | 0.00 | 0.01 | 0.9052 | 0.31 |
| 30116032 | Erlangsen A |  | 9 | 18,290,857 | rs7862648-G | 0.22 | 9.80E−07 | 1.12 | A | G | 0.02 | 0.01 | **0.0348** | 0.22 |
| 30116032 | Erlangsen A |  | 12 | 32,640,591 | rs112595860-G | 0.22 | 4.55E−07 | 0.88 | C | G | -0.01 | 0.01 | 0.6247 | 0.22 |
| 30116032 | Erlangsen A |  | 20 | 47,193,719 | rs4809706-G | 0.63 | 2.19E−07 | 0.9 | G | A | -0.01 | 0.01 | 0.1023 | 0.37 |
| 30116032 | Erlangsen A |  | 22 | 36,255,928 | rs150801052-A | 0.02 | 6.78E−07 | 1.51 |  |  |  |  |  |  |
| 28902444 | Stein MB | Suicide attempt in USA soldiers | 6 | 84,770,179 | rs2497117-A |  | 1.58E‐08 | 0.44 | A | G | -0.01 | 0.021 | 0.5387 | 0.05 |
| 28902444 | Stein MB |  | 6 | 84,771,964 | rs2497118-A |  | 1.70E‐08 | 0.44 | A | G | -0.01 | 0.021 | 0.5018 | 0.05 |
| 28902444 | Stein MB |  | 6 | 84,772,469 | rs2480192-T |  | 1.32E‐08 | 0.44 |  |  |  |  |  |  |
| 28902444 | Stein MB |  | 6 | 84,772,961 | rs2497119-A |  | 1.18E‐08 | 0.43 | A | G | -0.01 | 0.021 | 0.5190 | 0.05 |
| 28902444 | Stein MB |  | 6 | 84,794,805 | rs142060512-T |  | 3.55E‐08 | 2.84 | C | T | -0.04 | 0.03 | 0.1801 | 0.02 |
| 28902444 | Stein MB |  | 6 | 84,803,043 | rs116923768-A |  | 2.02E‐09 | 0.3 | A | T | -0.06 | 0.033 | 0.0651 | 0.02 |
| 28902444 | Stein MB |  | 6 | 84,809,043 | chr6_84809043_D-I2 |  | 4.68E‐09 | 0.32 |  |  |  |  |  |  |
| 28902444 | Stein MB |  | 6 | 84,820,786 | rs116878613-T |  | 4.12E‐09 | 0.3 | T | C | -0.07 | 0.036 | **0.0472** | 0.01 |
| 28902444 | Stein MB |  | 6 | 84,898,516 | rs117975834-C |  | 2.12E‐08 | 2.88 | G | C | -0.04 | 0.028 | 0.1220 | 0.02 |
| 28902444 | Stein MB |  | 6 | 84,914,920 | rs78022606-A |  | 4.14E‐08 | 2.41 |  |  |  |  |  |  |
| 28902444 | Stein MB |  | 6 | 84,935,294 | chr6_84935294_D-I5 |  | 4.96E‐10 | 0.35 |  |  |  |  |  |  |
| 28902444 | Stein MB |  | 6 | 84,935,441 | rs12524136-T |  | 5.24E‐10 | 2.88 | C | T | -0.03 | 0.023 | 0.2768 | 0.04 |
| 30655502 | Levey DF | suicide attempt severity | 15 |  | rs72740088-T |  | 7.49E-08 | -5.38 | T |  | 0.001 | 0.017 | 0.9690 | 0.07 |
| 30655502 | Levey DF |  | 1 |  | rs61520094-T |  | 8.14E-07 | 4.93 | C |  | 0.021 | 0.018 | 0.2560 | 0.07 |
| 30655502 | Levey DF |  | 9 |  | rs10867557-A |  | 4.73E-07 | -5.04 |  |  |  |  |  |  |
| 30655502 | Levey DF |  | 12 |  | rs1677091-A |  | 1.07E-08 | 5.72 | C |  | 0.005 | 0.01 | 0.614 | 0.28 |
| 30655502 | Levey DF |  | 12 |  | rs860447-T |  | 6.10E-07 | 4.99 | G | A | 0.003 | 0.01 | 0.7290 | 0.26 |
| Where: RAF, risk-allele frequency; EA, risk allele; OA, other allele. | | | | |  |  |  |  |  |  |  |  |  |  |
