# Supplementary material for: Identification of novel genome-wide associations for suicidality in UK Biobank, genetic correlation with psychiatric disorders and polygenic association with completed suicide
Source: eBioMedicine. 2019 Feb 8;41:517–25. doi: 10.1016/j.ebiom.2019.02.005 (PMC6442001; doi:10.1016/j.ebiom.2019.02.005)
Supplement: Supplementary file 19 — Supplementary material [file mmc19.docx]

**Supplementary Methods**

**Sample description**

In 2016-2017, 157,397 participants completed an online *‘Thoughts and Feelings’* questionnaire which included structured assessments of mental health, including suicidal thoughts and behaviours (1). Our study makes use of the full release of UK Biobank genetic data (2018). In order to maximise homogeneity, we have included only participants of self-reported white British ancestry. Participants’ date and cause of death were obtained from death certificates held within the National Health Service Information Centre (England and Wales), and National Health Service Central Register Scotland. At the time of analysis, mortality data were available up to the middle of February 2016. The final analytic sample was n=122 935 (Figure 1).

**Suicidality categories**

The self-harm and suicidality categories were based on four questions from the harm behaviours section of the online ‘Thoughts and Feelings’ questionnaire (Figure 1). In ascending severity these questions were: H1 “Many people have thoughts that life is not worth living. Have you felt that way?”; H2 “Have you contemplated harming yourself (for example by cutting, biting, hitting yourself or taking an overdose)?”; H3 “Have you deliberately harmed yourself, whether or not you meant to end your life?”; and H5 “Have you harmed yourself with the intention to end your life?”. Potential answers to H1 and H2 were “Yes, once.”, “Yes, more than once”, “No” and “Prefer not to answer”. Potential answers to H3 and H5 were “Yes”, “No” and “Prefer not to answer”. H5 was only asked if H3 was answered “Yes”. Participants were grouped into mutually exclusive categories of increasing severity of suicidality (Figure 1): category 0, ‘no suicidality’; SIA1, ‘thoughts that life was not worth living’; DSH1, ‘contemplated self-harm or suicide’; DSH2, ‘acts of deliberate self-harm not including attempted suicide’ and SIA2, ‘attempted suicide’. If participants met criteria for more than one category they were put into the most severe category. The comparator group was required to have answered “no” to all the questions. Linkage to death certification (until February 2016) identified a sub-group of participants classified as ‘completed suicide’=S3, (N=137).

**Sociodemographic and clinical characteristics**

The sociodemographic and clinical variables assessed included: sex (male, female); age (grouped into 35 to 44; 45 to 54; 55 to 64; and over 65), living arrangements (alone, husband, wife or partner, or other); social deprivation assessed using the Townsend deprivation (2); parental depression (neither, or at least one); chronic pain (free of pain, or pain at one or more sites); smoking status (never, previous or current); alcohol use (regular, occasional, former, or never); and childhood trauma (assessed using five items from the childhood trauma questionnaire (Bernstein, Stein et al. 2003). Participants were considered to have experienced childhood trauma if they reported emotional, physical or sexual abuse, physical neglect, or emotional neglect more than “rarely”. Major Depressive Disorder (MDD) was defined as endorsing either of two core symptoms: depressed mood (“Have you ever had a time in your life when you felt sad, blue, or depressed for two weeks or more in a row?”); or anhedonia (“Have you ever had a time in your life lasting two weeks or more when you lost interest in most things like hobbies, work, or activities that usually give you pleasure?”). These symptoms had to be present for most of the day, almost every day and cause impaired functioning. MDD diagnosis also required a total of five symptoms from: depressed mood; anhedonia; fatigue/low energy (“Did you feel more tired out or low on energy than is usual for you?”); sleep change (“Did your sleep change?”); poor concentration (“Did you have a lot more trouble concentrating than usual?”); worthlessness (“People sometimes feel down on themselves, no good, worthless - did you feel this way?”); weight change (“Did you gain or lose weight without trying, or did you stay about the same weight?”; and thoughts of death/dying (“Did you think a lot about death – either your own, someone else’s or death in general?”). Demographics of the participants included in this study are presented in eTable1.

**Participant selection for GWAS**

From the derived phenotypes we additionally excluded those who were not of self-reported white British ethnicity, those whose self-reported sex did not match their genetically determined sex, those with putative sex chromosome aneuploidy and those considered as outliers due to missing heterozygosity or had more than 10% missing genetic data. We then further excluded at random one person from each related pair of individuals with a kinship coefficient > 0·042 (second cousins) that have valid phenotypes.

**SNP exclusions**

SNPs were filtered by imputation score < 0·8, MAF < 0·01, HWE < 1x10^-6^ or missing more than 10% of the data leaving 9 481 578 SNPs for testing.

**Conditional analyses**

To test for multiple signals, analysis of the locus was repeated, using the same model as the primary analysis, with the further inclusion of the index SNP for the locus.

**PRS construction**

After QC and exclusions, SNPs were clumped by the r2 and LD window size. LD pruning was performed via PLINK on a random sample of 10 000 individuals using an r^2^>0·05 in a 250kb window. The SNP with the lowest *p* value for a given threshold was selected from each appropriate clump and used in the creation of the score, in PLINK (3), to produce a per-allele weighted score (without mean imputation) Where 2 or more SNPs from a set had the same p-value, the SNP with the larger beta coefficient was used. This pipeline uses all the SNPs in the dataset thus provides accurate LD block estimations (in contrast to software such as PRSice (4) which only clumps SNPs found in both the summary stats for the score and data set).

The SNP with the lowest p-value was selected from each of the LD-clumped SNP sets. The scores were calculated in PLINK to produce a per-allele weighted score (without mean imputation).

**PRS analyses of mood disorders and related traits**

For analysis of PRS effects on mood disorders and related traits, individuals who completed the online follow-up mental health questionnaire (known as the ‘Thoughts and Feelings’ questionnaire) were excluded. BD and MDD were self-reported at baseline. Analysis was conducted using logistic regression, adjusting for age, sex, chip and GPCs 1-8.

Related traits, namely mood instability (5), neuroticism (6), risk-taking behaviour (7, 8), circadian disruption (9) and chronotype (morning or evening preferences) were also analysed. Mood instability cases were defined as those who responded yes to the question “does your mood often go up or down?”, with those who responded no being considered as controls (5). Risk-takers were those who responded yes to the question “Would you consider yourself a risk taker?”, with those who responded no being considered controls (7, 8). Relative amplitude was calculated from accelerometer data collected over a 7-day period. (9, 10). Briefly, relative amplitude is the relative difference between the continuous 10 hour period with most activity and the continuous 5 hour period with least activity (9, 10). A mean value of relative amplitude was calculated for the 7-day period of measurement for each individual. Chronotype was based on self-report at baseline. In the analysis of Morning chronotype, those responding as being “definitely a morning person” being compared to all other responses and the analysis of evening chronotype comparing those responding as being “Definitely an evening person” to all other responses.

Mood instability, risk-taking behaviour and chronotype were analysed using logistic regression, adjusting for age, sex, genotyping chip, GPCs 1-8 and Townsend deprivation index. Relative amplitude and neuroticism score were analysed as linear traits, adjusting for age, sex, genotyping chip, GPCs 1-8 and Townsend deprivation index.

**Psychiatric disorders and related traits for genetic correlations**

Traits of interest included major depressive disorder (MDD), schizophrenia, bipolar disorder, anxiety disorder, attention deficit hyperactivity disorder (ADHD), post-traumatic stress disorder (PTSD), smoking behaviour, cannabis use (all downloaded from the Psychiatric Genetic Consortium, https://www.med.unc.edu/pgc/), suicide attempt(11), neuroticism (6), mood instability (12) and risk-taking (7) and relative amplitude(10)

**Gene-based analysis**

The ordinal GWAS results were also considered under a gene-based approach, using MAGMA(13), as implemented in FUMA(14). Briefly, SNPs were assigned to genes when they were located between the transcription start site and transcription end site. Composite *p* were computed for all protein-coding genes across the genome.

**Data mining**

# All GWAS significant SNPs were explored in the GTEx (15) and BRAINEAC datasets (http://braineac.org/). The GTEx data has previously been described (15). The BRAINEAC data set (http://braineac.org/) consists of consists of 134 individuals without neurological disorders. DNA was extracted from brain tissue and genotyping was conducted using the Illumina Infinium Omni1-Quad BeadChip and the Illumina Immuno Chip. After standard quality control procedures, imputation to the 1000 genomes reference panel was conducted. Brain tissues were dissected and RNA was extracted from each region of interest: cerebellar cortex (CRBL), frontal cortex (FCTX), hippocampus (HIPP), medulla (specifically inferior olivary nucleus, MEDU), occipital cortex (specifically primary visual cortex, OCTX), putamen (PUTM), substantia nigra (SNIG), thalamus (THAL), temporal cortex (TCTX) and intralobular white matter (WHMT). RNA was hybridised to Affymetrix Exon 1.0 ST Arrays and gene expression analysis conducted, adjusting for brain bank, gender and batch effects.

All genetic variants demonstrating suggestive evidence of association (*P*≤5x10^-5^) with suicidality were assessed for predicted effects using the Variant Effect Predictor (16). SNPs predicted to have more severe consequences than “non-coding-transcript-exon-variant” (<http://www.ensembl.org/info/genome/variation/predicted_data.html>) where taken forward to the GTEx and BRAINEAC database, to identify any genotype-specific gene expression patterns.

The GWAS catalogue was (<https://www.ebi.ac.uk/gwas/>) was queried to identify previous genome-wide genetic studies of deliberate self-harm (search terms: self-harm, self-injury) or suicidal behaviour (search terms: suicide, suicidal ideation) and for the loci (Chromosome 9 locus, 9:36999369-37360767; Chromosome 11 locus, 11:99392678-99588751; Chromosome 13 locus, 13:64900801-65036538) and genes within the loci (*CNTN5, ZCCHC7, PAX5, MELK, EBLN3, GRHPR, ZBTB5, POLR1E, FBXO10, TOMM5, FRMPD1, TRMT10B, EXOSC3, DCAF10, SHB, SCL25A51, OR7E156P, LINC00395*) identified here.

**References**

1. Davis KAS, Coleman JRI, Adams M, Allen N, Breen G, Cullen B, et al. Mental health in UK Biobank: development, implementation and results from an online questionnaire completed by 157 366 participants. BJPsych Open. 2018;4(3):83-90.

2. Townsend P, Phillimore P, Beattie A. Health and Deprivation: Inequality and the North. Croom Helm; 1988.

3. Purcell S, Neale B, Todd-Brown K, Thomas L, Ferreira MA, Bender D, et al. PLINK: a tool set for whole-genome association and population-based linkage analyses. Am J Hum Genet. 2007;81(3):559-75.

4. Euesden J, Lewis CM, O'Reilly PF. PRSice: Polygenic Risk Score software. Bioinformatics. 2015;31(9):1466-8.

5. Ward J, Strawbridge RJ, Bailey MES, Graham N, Ferguson A, Lyall DM, et al. Genome-wide analysis in UK Biobank identifies four loci associated with mood instability and genetic correlation with major depressive disorder, anxiety disorder and schizophrenia. Transl Psychiatry. 2017;7(11):1264.

6. Smith DJ, Escott-Price V, Davies G, Bailey ME, Colodro-Conde L, Ward J, et al. Genome-wide analysis of over 106 000 individuals identifies 9 neuroticism-associated loci. Mol Psychiatry. 2016;21(11):1644.

7. Strawbridge RJ, Ward J, Cullen B, Tunbridge EM, Hartz S, Bierut L, et al. Genome-wide analysis of self-reported risk-taking behaviour and cross-disorder genetic correlations in the UK Biobank cohort. Transl Psychiatry. 2018;8(1):39.

8. Strawbridge RJ, Ward J, Lyall LM, Tunbridge EM, Cullen B, Graham N, et al. Genetics of self-reported risk-taking behaviour, trans-ethnic consistency and relevance to brain gene expression Submitted.

9. Lyall LM, Wyse CA, Graham N, Ferguson A, Lyall DM, Cullen B, et al. Association of disrupted circadian rhythmicity with mood disorders, subjective wellbeing, and cognitive function: a cross-sectional study of 91 105 participants from the UK Biobank. Lancet Psychiatry. 2018;5(6):507-14.

10. Ferguson A, Lyall LM, Ward J, Strawbridge RJ, Cullen B, Graham N, et al. Genome-wide association study of circadian rhythmicity in 71,500 UK Biobank participants and polygenic association with mood instability. Submitted.

11. Erlangsen A, Appadurai V, Wang Y, Turecki G, Mors O, Werge T, et al. Genetics of suicide attempts in individuals with and without mental disorders: a population-based genome-wide association study. Mol Psychiatry. 2018.

12. Bulik-Sullivan B, Finucane HK, Anttila V, Gusev A, Day FR, Loh PR, et al. An atlas of genetic correlations across human diseases and traits. Nat Genet. 2015;47(11):1236-41.

13. de Leeuw CA, Mooij JM, Heskes T, Posthuma D. MAGMA: generalized gene-set analysis of GWAS data. PLoS Comput Biol. 2015;11(4):e1004219.

14. Watanabe K, Taskesen E, van Bochoven A, Posthuma D. Functional mapping and annotation of genetic associations with FUMA. Nat Commun. 2017;8(1):1826.

15. Consortium GT. The Genotype-Tissue Expression (GTEx) project. Nat Genet. 2013;45(6):580-5.

16. McLaren W, Gil L, Hunt SE, Riat HS, Ritchie GR, Thormann A, et al. The Ensembl Variant Effect Predictor. Genome Biol. 2016;17(1):122.
